# Effects of dietary protein level on small intestinal morphology, occludin protein, and bacterial diversity in weaned piglets

**DOI:** 10.1002/fsn3.2828

**Published:** 2022-03-21

**Authors:** Zhihua Ren, Haoyue Fan, Huidan Deng, Shuhua Yao, Guilin Jia, Zhicai Zuo, Yanchun Hu, Liuhong Shen, Xiaoping Ma, Zhijun Zhong, Youtian Deng, Renjie Yao, Junliang Deng

**Affiliations:** ^1^ 12529 College of Veterinary Medicine Sichuan Agricultural University Ya’an China; ^2^ Sichuan Province Key Laboratory of Animal Disease & Human Health Ya’an China; ^3^ Key Laboratory of Environmental Hazard and Human Health of Sichuan Province Ya’an China

**Keywords:** crude protein levels, diversity of intestinal bacteria, morphology and structure of small intestine, occludin protein

## Abstract

Due to the physiological characteristics of piglets, the morphological structure and function of the small intestinal mucosa change after weaning, which easily leads to diarrhea in piglets. The aim of this study was to investigate effects of crude protein (CP) levels on small intestinal morphology, occludin protein expression, and intestinal bacteria diversity in weaned piglets. Ninety‐six weaned piglets (25 days of age) were randomly divided into four groups and fed diets containing 18%, 20%, 22%, and 24% protein. At 6, 24, 48, 72, and 96 h, changes in mucosal morphological structure, occludin mRNA, and protein expression and in the localization of occludin in jejunal and ileal tissues were evaluated. At 6, 24, and 72 h, changes in bacterial diversity and number of the ileal and colonic contents were analyzed. Results showed that structures of the jejunum and the ileum of piglets in the 20% CP group were intact. The expression of occludin mRNA and protein in the small intestine of piglets in the 20% CP group were significantly higher than those in the other groups. As the CP level increased, the number of pathogens, such as *Clostridium difficile* and *Escherichia coli*, in the intestine increased, while the number of beneficial bacteria, such as *Lactobacillus, Bifidobacterium*, and *Roseburia*, decreased. It is concluded that maintaining the CP level at 20% is beneficial to maintaining the small intestinal mucosal barrier and its absorption function, reducing the occurrence of diarrhea, and facilitating the growth and development of piglets.

## Graphical abstract

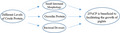

## 1 INTRODUCTION

The development of the piglet breeding industry plays an important role in the development of China’s agricultural economy. Due to the physiological characteristics of piglets, the morphological structure and function of the small intestinal mucosa change after weaning, which easily leads to diarrhea in piglets (Kwon et al., 2014; Wijtten et al., 2011). Therefore, antibacterial growth promoters are commonly used to improve the growth and development of weaned piglets and to inhibit the reproduction of pathogenic bacteria. However, with the continuous increase in drug resistance risk, it is urgently important to identify another method of piglet husbandry. Protein is an indispensable nutrient for the growth of piglets, but piglets are very sensitive to crude protein (CP) levels. Lower CP levels reduce villus height and crypt depth in the small intestine, affect the balance of microbiota, decrease the digestion and utilization of proteins in the intestine, and decrease the growth performance of piglets (Luo et al., 2015; Peng et al., 2016, 2017), whereas excessive CP levels result in a large amount of undigested and utilized proteins entering the intestine and fermenting spoilage and increase the pH in the stomach and small intestine, thereby providing suitable conditions for colonization of the intestine by pathogens and disrupting the balance of intestinal bacteria, leading to diarrhea (Gao et al., 2020; Zhang et al., 2020).

As one of the organs connected to the outside world, the small intestine is continuously exposed to protein antigens and to bacteria and their degradation products, and it prevents these harmful substances from entering other parts of the body through its barrier function. The small intestinal mucosal barrier includes a mechanical barrier, a biological barrier, an immune barrier, and a chemical barrier. The mechanical barrier is primarily composed of small intestinal mucosal epithelial cells and intercellular tight junctions and is important to the small intestinal mucosal barrier.

Tight junctions, the primary components of the intestinal cell barrier, are composed of the tight junction proteins occludin, claudin, and ZO‐1; these proteins function to strengthen intercellular junctions, avoid cell damage, and resist invasion by harmful substances and pathogenic microorganisms (Zihni et al., 2016). Occludin proteins play an important role in maintaining the integrity of tight junctions, as well as in maintaining small intestinal permeability (Buckley & Turner, 2018; Shil et al., 2020; Teng et al., 2020). Lochhead et al. found that (Lochhead et al., 2010) the outer loop of occludin protein is directly inserted into tight junctions and that the outer loop and the transmembrane portion interact with tight junctions; in this way, the membrane permeability at the junction site is reduced, and free access of macromolecules is blocked, to achieve barrier protection. Under pathological conditions, occludin protein produces a contraction phenomenon and moves into the cytoplasm, resulting in the expansion of intercellular pores and destruction of the integrity between cells and increasing the translocation of macromolecules, toxins, and bacteria, which can easily lead to diarrhea (Khounlotham et al., 2012). Biological barriers to intestinal bacteria, which are the first barriers through which animals defend themselves against foreign pathogens, not only resist invasion by pathogens, participate in the metabolic synthesis of nutrients, and provide nutrition for the body but also regulate the host’s intestinal immune system, interact with each other, and jointly maintain the homeostasis of the small intestinal microbial environment (Turkez et al., 2012). Weaning of piglets causes changes in intestinal microflora, reduced bacterial diversity, loss of appetite, diarrhea, and other phenomena; therefore, nutrients should be reasonably supplemented to improve the animals’ performance during this period.

At present, diarrheal disease has been the cause of high mortality in children (Liu et al., 2019; Taborda et al., 2018). How to control infant diarrhea by regulating the level of protein has been a focus of recent research (Gao et al., 2020). The piglet model has become the best model for human nutrition. The growth of piglets is related to a variety of factors, such as a daily three‐meal pattern and CP levels (Xie et al., 2020). In previous experiments, we found that 20%–24% CP would cause diarrhea in weaned piglets in the short term (Dong et al., 2019). In order to find a more appropriate CP level for piglet development in the short term, we not only investigated how changes in CP levels affect the morphological structure and the expression and distribution of occludin protein in the intestine of weaned piglets, but also explored the relationship between intestinal bacterial and diarrhea. In addition, we hope that provides a reference for the level of protein intake in infants.

## 2 MATERIALS AND METHODS

### 2.1 Animals and experimental design

Ninety‐six weaned Du × Long × Large ternary crossbred piglets (25 days of age, and initial weight of 5.99 ± 1.07 kg) were purchased from Zhiping Farm in Qionglai City, Sichuan Province and randomly divided into four groups with four replicates of six pigs each. After 7 days of adaptation to feeding, they were fed diets with CP levels of 18%, 20%, 22%, and 24%. Each piglet was individually housed, and the piglets in each group had free access to food and water. The piglets were housed in fully enclosed enclosures with leaky floors, teat‐type drinkers, and adjustable stainless‐steel tanks. The room temperature was controlled at 28–30℃, the relative humidity was controlled at 60%–70%, and the pig house was regularly ventilated. The environment and the appliances within the pig house were cleaned and disinfected before the test. Disinfection, deworming, and immunization were performed regularly according to the procedures of the pig farm during the entire test period. After feeding for 6, 24, 48, 72, and 96 h, four piglets were randomly selected from each group (one replicate/group). After collection of blood from the anterior vena cava, piglets in good condition were anesthetized with an intramuscular injection of ketamine (20 mg) and tranquilizers (0.2 mg). Approximately 5–15 min after injecting ketamine, the piglet is positioned on its side to facilitate breathing and intravenous cannulation of an ear vein can be performed. Then the abdominal cavity was opened, and the outer wall and the abdominal contents were washed with PBS solution in an ice bath. Two segments of jejunum and two segments of ileum, each measuring 50 mm in length and no more than 0.5 cm in width, were taken; one segment from each tissue was placed in 4% paraformaldehyde for fixation (the volume of fixative was 10–15 times the tissue volume) for pathological observation of the small intestine and occludin protein localization observation, and the other was numbered, wrapped in aluminum foil, and immediately cryopreserved in liquid nitrogen for use in the detection of the expression of occludin protein and the relative content of occludin mRNA. In addition, 0.5‐cm ileal intestinal segments were taken, placed in 3% glutaraldehyde fixative, and stored in a refrigerator at 4℃ for observation of ileal epithelial tissue ultrastructure. The ileal and colonic contents of piglets fed for 6, 24, and 72 h were aseptically removed, loaded into 2 ml cryogenic vials, fully mixed and placed in liquid nitrogen (snap‐frozen samples, numbered as shown in Table 1‐1), and stored in a −80℃ freezer. These samples were used for extraction of total bacterial DNA and determination of the copy numbers of *Lactobacillus*, *Bifidobacterium*, *Clostridium difficile*, *Escherichia coli* (*E. coli*), and *Roseburia* in each sample.

**TABLE 1‐1:** Sample collection number

Sampling time (h)	Ileum				Colon			
	18% CP	20% CP	22% CP	24% CP	18% CP	20% CP	22% CP	24% CP
6	A1	B1	C1	D1	E1	F1	H1	G1
24	A2	B2	C2	D2	E2	F2	H2	G2
72	A3	B3	C3	D3	E3	F3	H3	G3

### 2.2 Diets

Table 1‐2 shows the nutrient composition of the experimental diets. A corn–soybean meal‐based diet suitable for the piglet stage (5–10 kg) was prepared according to the NRC criteria (2012). Soybean meal and corn were used to adjust the protein levels of the diets, and soybean oil was used to adjust the dietary energy level while balancing the levels of lysine, methionine, threonine, and tryptophan.

**TABLE 1‐2:** Composition and nutrient levels of the basal diet

Items	Content %			
	18% CP	20% CP	22% CP	24% CP
Corn	56	49.2	44.2	37.5
Soybean meal	24	31	37.5	43.5
Wheat bran	2.7	2.8	3	3
Soybean oil	2	2	1.35	1.26
Whey powder	6	6	6	6
Fish meal	5.5	5.5	5.5	5.5
L‐lys	0.46	0.29	0.12	0.12
*DL*‐Met	0.09	0.06	0.04	0.04
*L*‐Thr	0.13	0.05	0.01	0.01
Choline chloride	0.1	0.1	0.1	0.1
Limestone	0.66	0.28	0.74	0.74
CaHPO_4_	1.33	1.5	0.58	0.58
NaCl	0.25	0.25	0.25	0.25
Premix^a^	0.25	0.25	0.25	0.25
Rice chaff	0.53	0.72	0.36	1
Total	100.00	100.00	100.00	100.00
Nutrient levels^b^				
ME (MJ/kg)	16.37	16.29	16.31	16.28
CP	18.54	20.19	21.77	23.84
Lys	0.69	0.66	1.00	0.91
Met + Cys	0.41	0.37	0.54	0.51
Thr	0.84	0.72	1.00	0.90
Try	0.49	0.54	0.82	0.79
Arg	0.87	0.91	1.57	1.56
His	0.33	0.35	0.55	0.52
Ile	0.52	0.58	0.92	0.86
Leu	1.09	1.19	1.91	1.69
Phe	0.66	0.75	1.19	1.09
Val	0.63	0.70	1.06	0.98
EAA	6.53	6.77	10.56	9.81
NEAA	6.76	7.49	11.76	10.62
EAA/TAA	0.49	0.47	0.47	0.48

^a^ Premix provides the following per kg of diets: VA 7000 IU, VD_3_ 2000 IU, VE 15 IU, VK_3_ 2 mg, VB_1_ 2 mg, VB_2_ 5 mg, biotin 0.08 mg, VB_6_ 3 mg, VB_12_ 0.02 mg, niacin 20 mg, *D*‐pantothenic acid 10 mg, folic acid 10 mg, ethoxyquin 0.1 mg, Cu (CuSO_4_.5H_2_O) 6 mg, Fe (FeSO_4_·H_2_O) 100 mg, I (KI) 0.14 mg, Mn (MnSO_4_·H_2_O) 4 mg, Zn (ZnSO_4_·H_2_O) 100 mg, Se (Na_2_Se_3_O_3_) 0.3 mg.

^b^ Calculated values.

### 2.3 Histopathological observation of the small intestine

Preparation of tissue sections: After the jejunum and ileum were fixed for 24 h, the tissues were trimmed and embedded, dehydrated by incubation in a graded alcohol series (70% 1 h, 85% 1 h, 95% 1 h, 100% I 30 min, 100% II 1 h, 100% III 30 min), becoming successively transparent, soaked in wax, embedded, sectioned (5–8 µm thick), examined, and baked (60℃ for 1 h).

The sections were rehydrated by incubation in a graded alcohol series (100% alcohol for 5 min, 95% alcohol for 2 min, 85% alcohol for 2 min, 75% alcohol for 2 min, and distilled water for 2 min) and subsequently incubated in hematoxylin staining solution for 10 min, in hydrochloric acid/alcohol for 3–5 s, in tap water for 10 min, and in eosin staining solution for 8 min. The sections were finally dehydrated and made transparent by incubation in 95% alcohol II for 1 min, 100% alcohol II for 15 min, xylene I for 15 min, and xylene II for 15 min. After the above steps were completed, the sections were dried and mounted with neutral resin; pathological tissue changes were observed under a light microscope, and photographs were taken and recorded.

### 2.4 Ultrastructural observation of ileal epithelial tissue

The ileal tissues were fixed in 3% glutaraldehyde, dehydrated stepwise with propionaldehyde (30%, 50%, 70%, 80%, 90%, 95%, and 100%), permeabilized (dehydrating agent and epoxy resin permeabilization solution in proportions of 3:1, 1:1, and 1:3, respectively, for 60 min each time), embedded, ultrathin sectioned (50 nm), stained with uranyl acetate and lead citrate for 15–20 min, dried, observed under a transmission electron microscope, and photographed.

### 2.5 Immunohistochemical observation of small intestinal tissue

For immunohistochemistry, the embedded wax blocks of jejunal and ileal tissues were sliced, baked, deparaffinized, and rehydrated. After the sections were treated with 3% H_2_O_2_ for 15 min, they were immersed in a beaker filled with 0.01 mol/L citric acid buffer and placed in a 96℃ water bath to slowly reach a temperature of 95℃. After this temperature had been maintained for 20 min, the samples were removed from the water bath and allowed to cool naturally to room temperature. The sections were subsequently incubated successively in serum blocking solution (37℃ for 30 min), primary antibody (1:100, overnight at 4℃), secondary antibody (1:100, 1 h at 37℃), and SABC (1:100, 20 min at 37℃), DAB chromogenic solution was added, and the stained samples were observed under a microscope. The optimal staining time was selected followed by hematoxylin restaining for 1 min. The differentiation solution was applied for 3–5 s. After each step, the samples were washed with PBS and dried. Finally, the samples were dehydrated (75% ethanol for 3 min, 85% ethanol for 3 min, 95% ethanol for 3 min, and 100% ethanol for 3 min), made transparent, gum mounted, and microscopically examined, and photographed. The stained images were analyzed using Image pro plus 6.0 software; the average optical density value for five different fields was calculated, and the average value was used to represent the expression level of occludin protein.

### 2.6 Detection of occludin mRNA in the small intestine

Table 1‐3 shows the gene (serial number: NM001163647.1) and the internal reference gene sequence of porcine occludin. Primers were designed with Primer 5.0 and synthesized by Shenggong Biotech (Shanghai) Engineering Co., Ltd. Before capping, the synthesized primers were centrifuged at 1776 *g* for 30–60 s and subsequently diluted with sterilized ultrapure water to the storage concentration (100 μmol/L). The mixed solution was shaken well and stored at −20℃. For use, the primers were diluted with sterile ultrapure water to the working concentration (10 μmol/L). The relative expression levels of occludin mRNA in the jejunum and ileum were measured by RT‐qPCR.

**TABLE 1‐3:** The primers used for RT‐qPCR

Name	Primer sequence (5′–3′)	Target fragment (bp)
Occludin	F: CAGCCTCATTACAGCAGCAGTGG R: ATCCAGTCTTCCTCCAGCTCGTC	158
β‐actin	F: GCATCCACGAGACCACCTTCAAC R: GACAGCACCGTGTTGGCGTAG	82

### 2.7 Detection of occludin protein expression in the small intestine

According to the instructions provided with the animal whole protein extraction kit, total protein was extracted from jejunum and ileum tissues, and the protein concentration of the extract was determined according to the instructions provided with the BCA protein concentration determination kit. Subsequently, 50 μg of total protein was subjected to SDS‐PAGE electrophoresis (S1: 90 V, 15 min; S2: 120 V, 30 min), membrane transfer (ice bath, 200 mA), blocking (5% milk for 1 h), incubation with primary antibody (1:1000, overnight at 4℃ on a shaker), washing of the membrane, incubation with secondary antibody (1:5000, 1 h at room temperature), color development (room temperature for 5 min), exposure, development, fixation, and air drying at room temperature, and the film was scanned or photographed.

### 2.8 Determination of bacterial diversity and population structure

Total bacterial DNA was extracted from 200 mg of each sample using a fecal DNA extraction kit. The integrity of the genomic DNA was assessed by 2% agarose gel electrophoresis (30 min at 100 V), and the DNA concentration and purity were determined using a micro‐UV spectrophotometer (Nanodrop 2000). The extracted total genomic DNA was stored at −20℃ until use.

The extracted DNA samples were diluted to 1 ng/μl in sterile water and sent to Beijing Nuohe Zhiyuan Technology Co., Ltd. The diluted genomic DNA was used as a template for PCR using primers specific for bacterial 16S rDNA 4 regions and Barcode, Phusion^®^ High‐Fidelity PCR Master Mix with GC Buffer, and efficient high‐fidelity enzymes to ensure the efficiency and accuracy of the amplification.

The primers (Santiago et al., 2016) used were 515F (5′‐GTG CCAGCMGCCGCGGTAA‐3′) and 806R (5′‐GGACTACHVGGGTW TCTAAT‐3′).

Electrophoresis on 2% agarose gels was used to detect the PCR products; equal amounts of the samples were combined according to the concentration of PCR products and mixed well; electrophoresis was then used to detect the PCR products, and the target bands were recovered. A library was constructed using the TruSeq^®^ DNAPCR‐Free Sample Preparation Kit library construction kit, and the constructed library was quantified by Qubit and Q‐PCR. After assessment of the quality of the library, double‐end sequencing was performed using HiSeq2500PE250.

### 2.9 Determination of the copy number of *Lactobacillus*, *Bifidobacterium*, *Clostridium difficile*, *E. coli*, and *Roseburia*

The 16S rDNA sequences of various strains were searched on GenBank, primer design was performed using Primer Express 5.0 (Table 1‐4), and primer specificity was detected by BLAST in GenBank. Primers were synthesized by Shanghai Shenggong Biotech Co., Ltd.

**TABLE 1‐4:** Primers for major bacteria in the intestine

Species	Primer sequences 5′–3′	Annealing temp (℃)	Product size (bp)	Accession
*Lactobacillus*	F: CGCACTGTATATGAGGAGCTGACG R: CCACTCACGACGACCACAATCAC	61.0	195	GenBank: CP032464.1
*Bifidobacterium*	F: CTTGGTGGTGAGAGTGGCGAAC R: TCAACTGGAACATCCGGCATTACC	61.0	104	GenBank: AP010889.1
*Clostridium difficile*	F:AGCAGTTGAATATAGTGGTTTAGTTAGAGTTG R: CATGCTTTTTTAGTTTCTGGATTGAA	61.0	144	GenBank: CP019857.1
*Escherichia coli*	F: CGCGCCAGTGAACGGTATCG R: TGTCGTCCGCTGGATCCTGAC	63.0	128	GenBank: MG904991.1
*Roseburia*	F: GCGGTACGGCAAGTCTGATGTG R: CGCCTTCGCCACTGGTGTTC	63.0	149	GenBank: NR_117758.1

Note

F, upstream primer; R, downstream primer.

Total DNA from intestinal digesta was used as a template for PCR amplification using the above five pairs of specific primers. The recovered target fragment was ligated into the pMD19‐T vector and transformed into competent *E*. *coli* DH5α. The colonies were determined by PCR. Bacteria identified as positive colonies were sent to Shanghai Sangon Biotech Co., Ltd. for automatic sequencing. The sequencing results were compared for homology on BLAST at NCBI. The identity of the bacterium was confirmed. Each plasmid was extracted with a small amount of plasmid extraction kit and used as the standard. The extracted plasmids were identified by common PCR according to their respective primers and reaction procedures to check the size and integrity of the bands.

The concentration of the standard (plasmid) was measured on a Nanodrop 2000. The copy number of the positive plasmid was calculated according to the following formula: plasmid copy number (copies/μl) = DNA concentration (ng/μl) × 6.02 × 10^23^ (copies/mol) × 10^−9^/[plasmid length (dp) × 660 (g/mol·dp)], and the known pMD19‐T vector was 2692 bp in length.

Absolute RT‐qPCR quantification was performed on a CFX96 Real‐Time PCR System using the various primers, and dissolution curves were automatically generated after the reaction ended.

### 2.10 Statistical analysis

The data for each sample were split from the offline data according to the Barcode sequence and PCR amplification primer sequence, and the reads of each sample were spliced using FLASH after truncating the Barcode and primer sequences. The resulting spliced sequences were the original Tags data (Raw Tags); the spliced Raw Tags required strict filtering (Bokulich et al., 2013) to obtain high‐quality Tags data (Clean Tags). The Tags obtained through the Tags quality control process of Qiime (Caporaso et al., 2010) need to be processed to remove chimeric sequences; these are detected by alignment with the species annotation database, and, finally, the chimeric sequences are removed to obtain the final effective data (Effective Tags).

Using Uparse software, the Effective Tags of all samples were clustered for Operational Taxonomic Units (OTUs) with 97% identity; the most abundant sequence in each OTU was then selected as the representative sequence of that OTU. Species annotation was performed on OTUs’ representative sequences, and species annotation analysis was performed with the Mothur (Edgar, 2013) method with the SSU rRNA database of SILVA at a threshold of 0.8–1 to obtain the taxonomic information corresponding to each OTU.

QIIME software was used to construct a dilution curve for the number of sequences and the corresponding number of OTUs. The dilution curve was prepared by randomly selecting a certain amount of sequencing data from the sample, counting the number of species the data represented (i.e., the number of OTUs), and constructing a curve based on the amount of sequencing data drawn and the corresponding number of species. The alpha diversity index (Chao1, Shannon, Simpson, ACE) was calculated using IIQME software (Version 1.9.1).

PCA analysis of community composition structure at the genus level was performed using R software.

The data were collated using Excel, and the results were analyzed by one‐way analysis of variance using SPSS Statistics 22 statistical software with *p* < .05 as the criterion for discriminating significant differences. The results are expressed as the mean ± standard deviation.

## 3 RESULTS

### 3.1 Effect of CP levels on the histopathology of the small intestine in weaned piglets

#### 3.1.1 Histopathological changes in the jejunum

In Figures 1‐1, 1‐2, 1‐3, 1‐4, 1‐5, it can be seen that after feeding for 6, 24, and 48 h, most of the jejunal villous epithelium in the 20% and 22% CP groups had edges that were smooth and uniform in length in the visual fields shown in the figure, and the epithelial cells were arranged neatly, whereas in the jejunum of the 18% and 24% CP groups, large numbers of mucosal cells were necrotic and detached, the intestinal villous epithelial edges were damaged, and the epithelial cells were unevenly arranged. At 72 h, the 18% and 20% CP groups had more damage to the edges of the jejunal villi, and a small proportion of shed villous epithelial cells had infiltrated the intestinal lumen. At 96 h, the jejunal villi of the 18% and 20% CP groups had returned to normal tissue structure, the intestinal villous epithelium was intact, the length of the villi was consistent, the epithelial cells were arranged neatly, and the crypts were clearly visible, while the jejunal villi of the animals in the 22% CP group displayed severe injury; in the animals of the 22% CP group, large numbers of epithelial cells were detached and had infiltrated the intestinal lumen, and the apical epithelial cells of jejunal villi of the 24% CP group had completely detached.

**FIGURE 1‐1:**
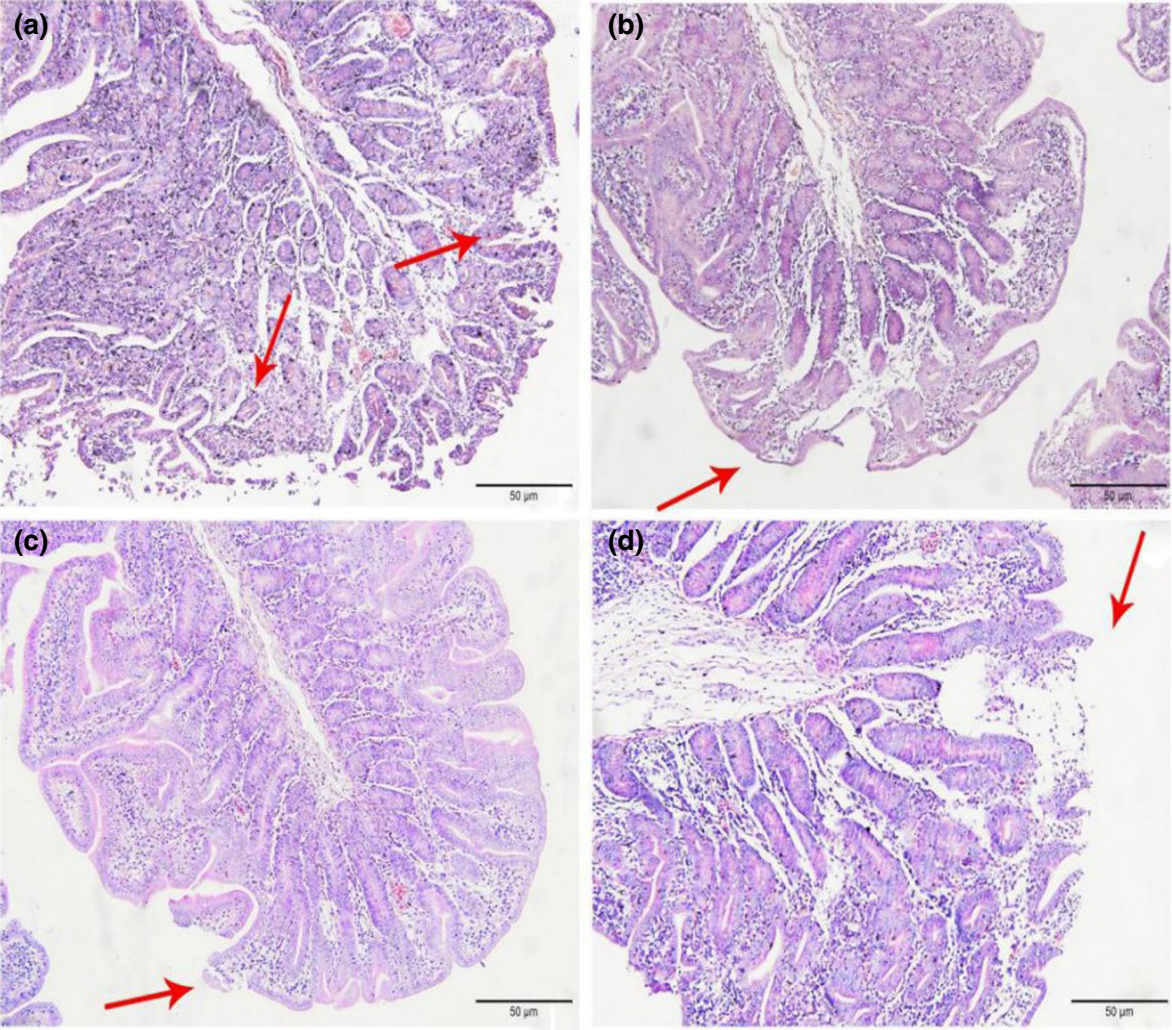
The pathological injury of jejunum in each group on 6 h after feeding. (×200). (a–d represents the groups of different CP level 18%, 20%, 22%, 24%, respectively. The arrow points to the villus injury site.)

**FIGURE 1‐2:**
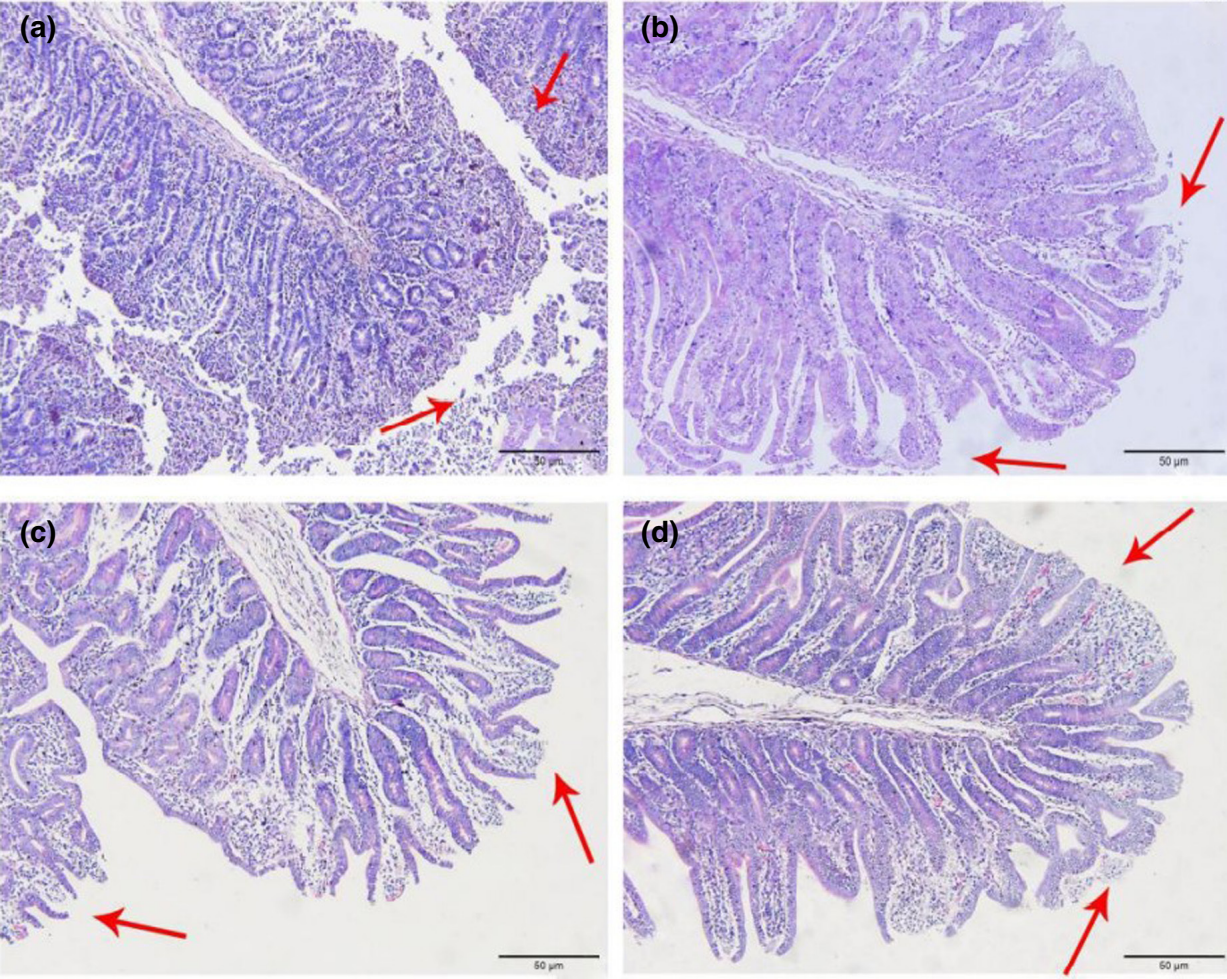
The pathological injury of jejunum in each group on 24 h after feeding. (×200). (a–d represents the groups of different CP level 18%, 20%, 22%, 24%, respectively. The arrow points to the villus injury site.)

**FIGURE 1‐3:**
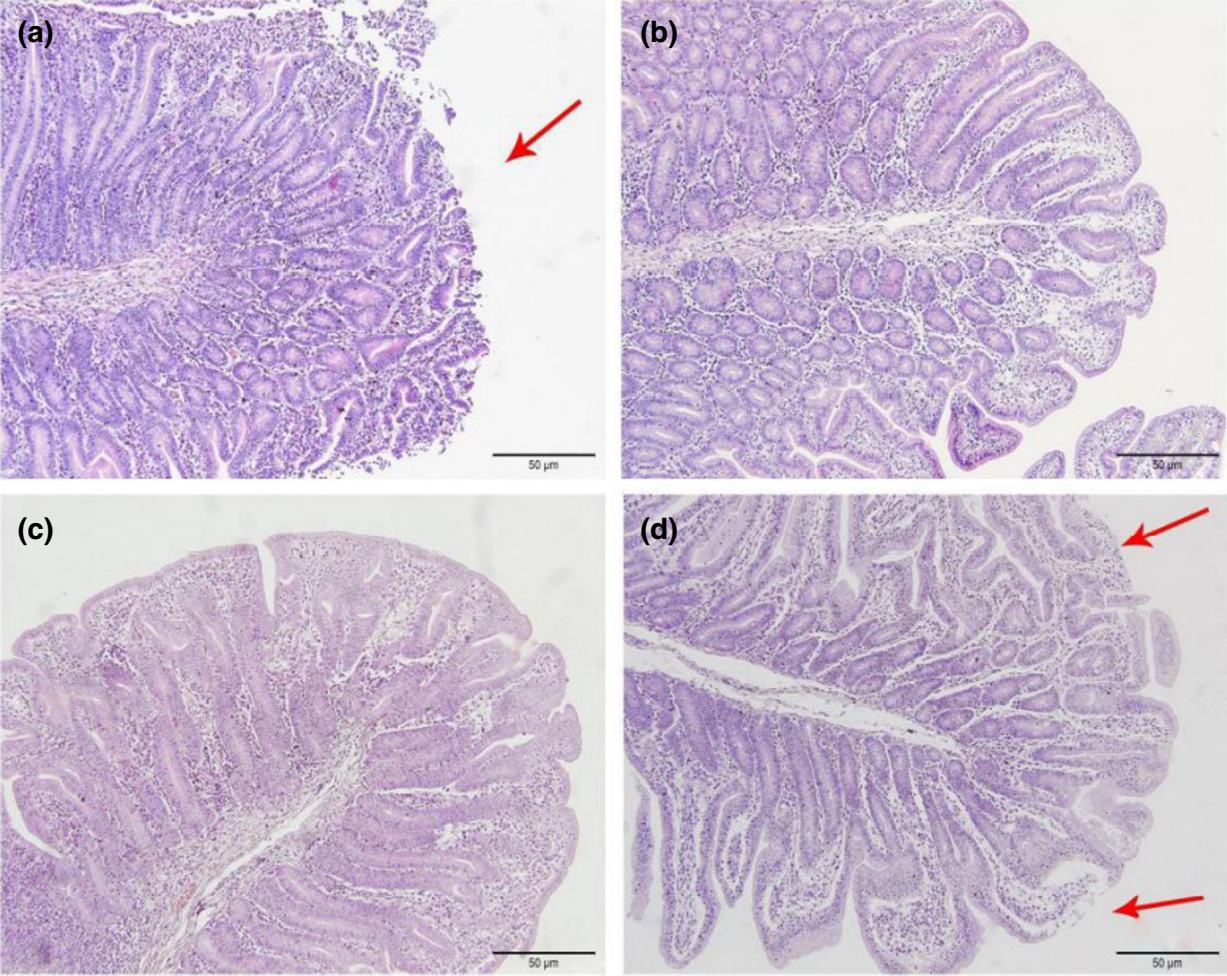
The pathological injury of jejunum in each group on 48 h after feeding. (×200). (a–d represents the groups of different CP level 18%, 20%, 22%, 24%, respectively. The arrow points to the villus injury site.)

**FIGURE 1‐4:**
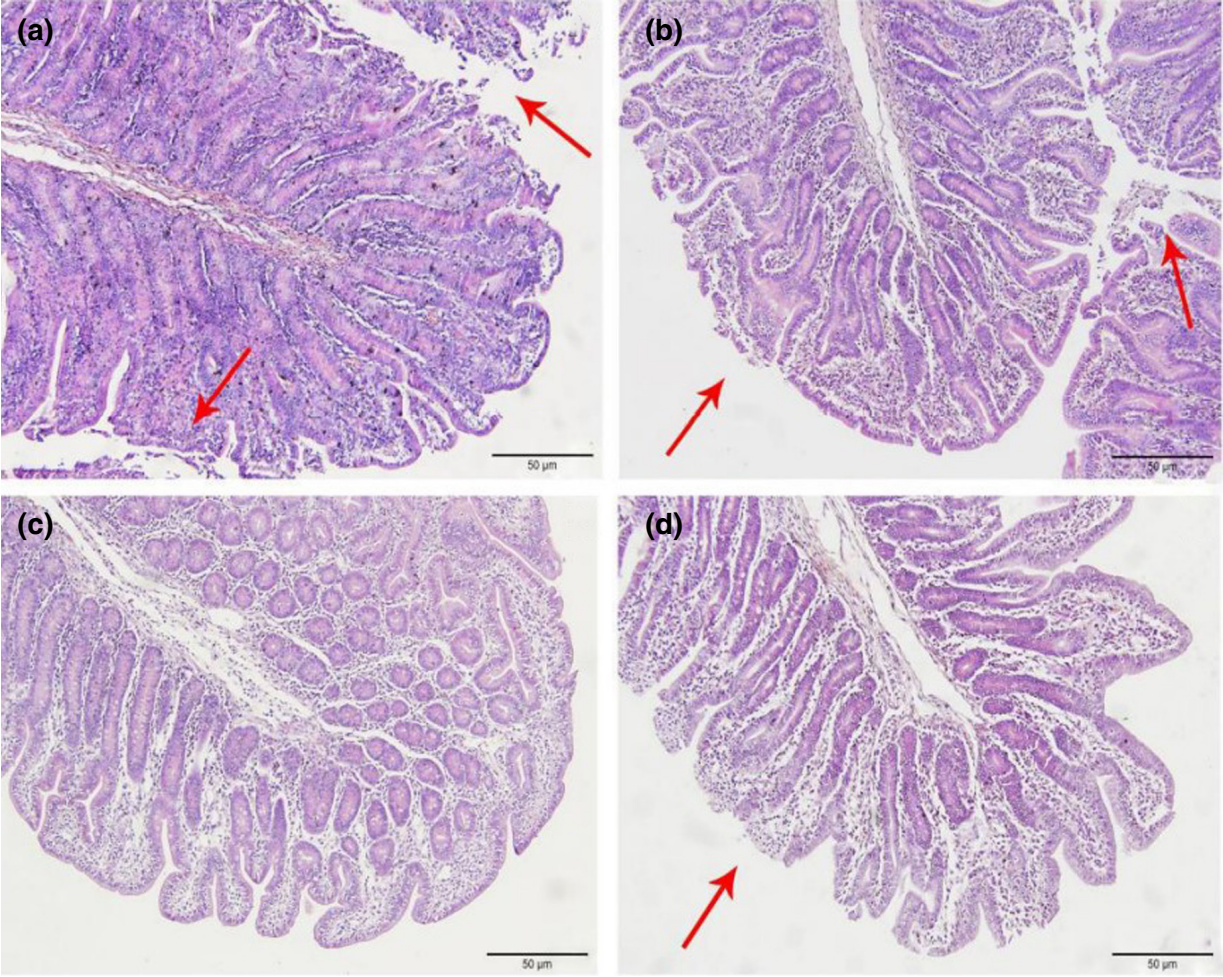
The pathological injury of jejunum in each group on 72 h after feeding. (×200). (a–d represents the groups of different CP level 18%, 20%, 22%, 24%, respectively. The arrow points to the villus injury site.)

**FIGURE 1‐5:**
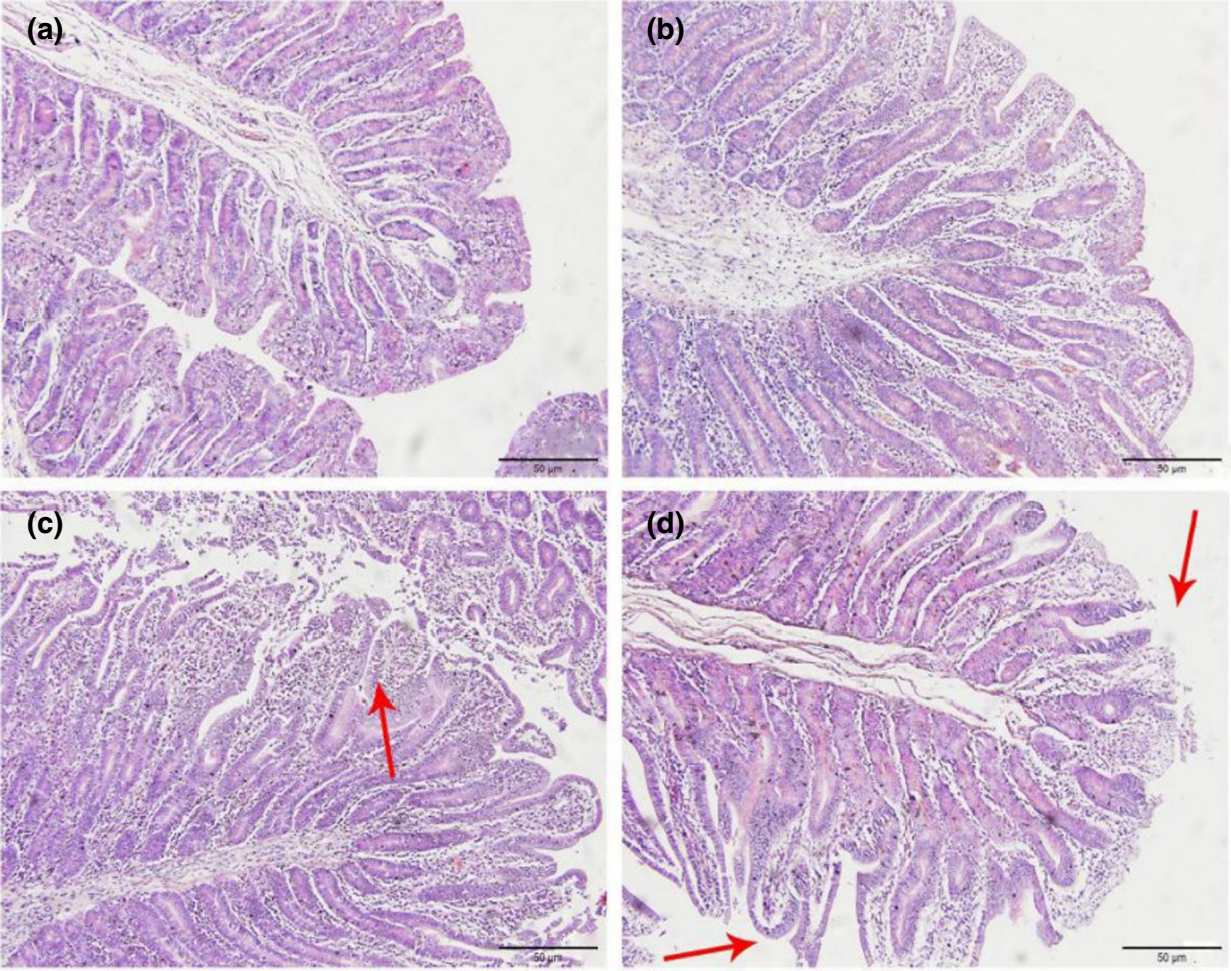
The pathological injury of jejunum in each group on 96 h after feeding. (×200). (a–d represents the groups of different CP level 18%, 20%, 22%, 24%, respectively. The arrow points to the villus injury site.)

#### 3.1.2 Histopathological changes in the ileum

As shown in Figures 1‐6, 1‐7, 1‐8, 1‐9, 1‐10, in the 20% CP group, the edge of the ileal villous epithelium was smooth (except at 48 h), and the epithelial cells were arranged neatly at 6, 24, and 48 h, while in the other three groups, necrosis and shedding of some ileal villous epithelial cells were observed, and the edge of the intestinal villous epithelium was damaged with uneven and uneven arrangement of epithelial cells. At 72 h, the damage to the ileal villi in the four groups gradually recovered; the ileal villi in the 18%, 20%, and 22% CP groups were neatly arranged and uniform in length, but their edges were not smooth and they tended to appear damaged; more seriously, necrosis and shedding occurred in the apical epithelial cells of the ileal villi in the 24% CP group, showing partial crypt hyperplasia. At 96 h, the edges of the ileal villi were flat and neatly arranged, and the villi were of consistent length in the 18% and 20% CP groups; in the 22% CP group, some intestinal villi in the ileum were still ruptured, but no exfoliated intestinal epithelial cells were observed in the intestinal lumen.

**FIGURE 1‐6:**
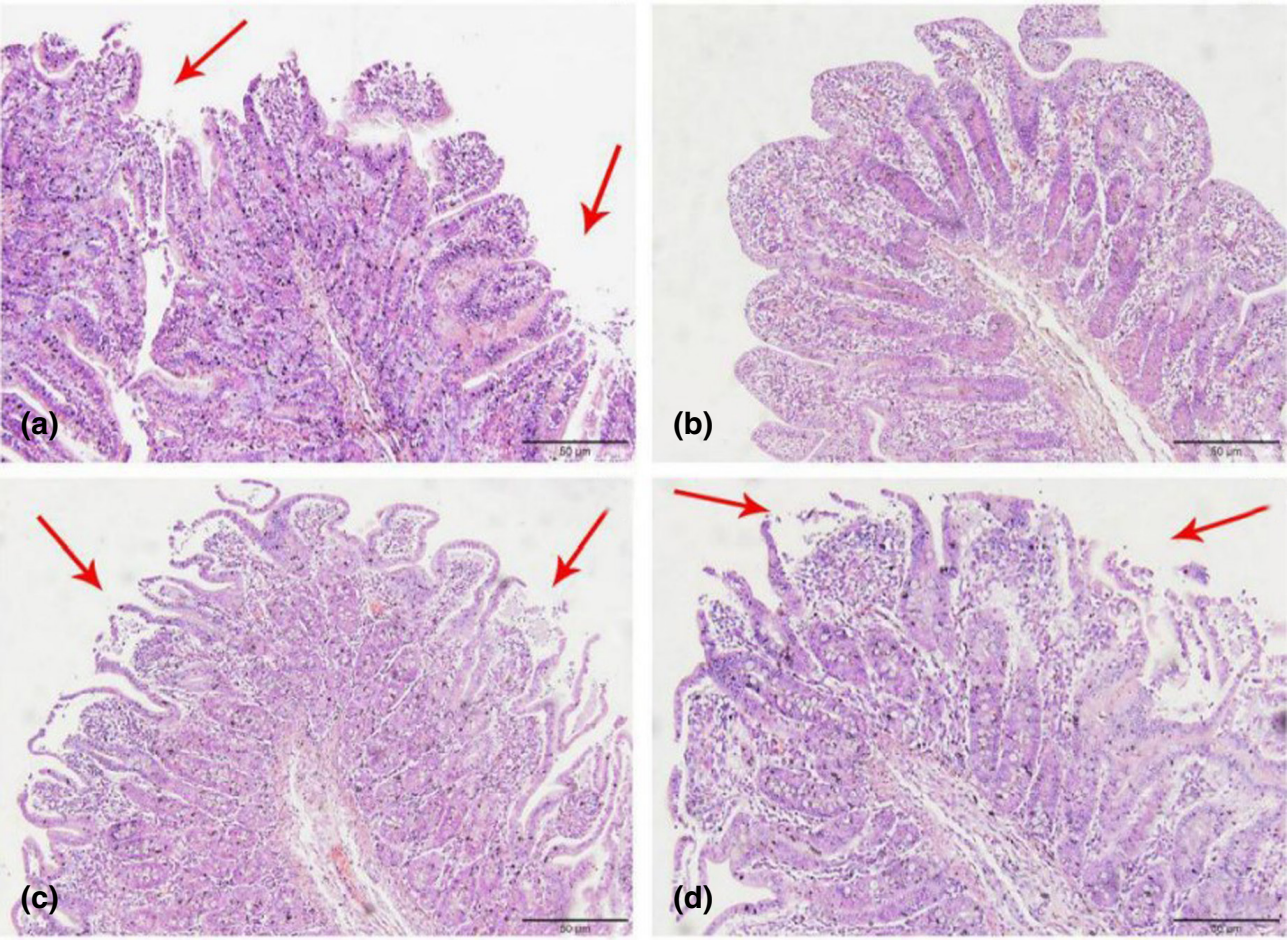
The pathological injury of ileum in each group on 6 h after feeding. (×200). (a–d represents the groups of different CP level 18%, 20%, 22%, 24%, respectively. The arrow points to the villus injury site.)

**FIGURE 1‐7:**
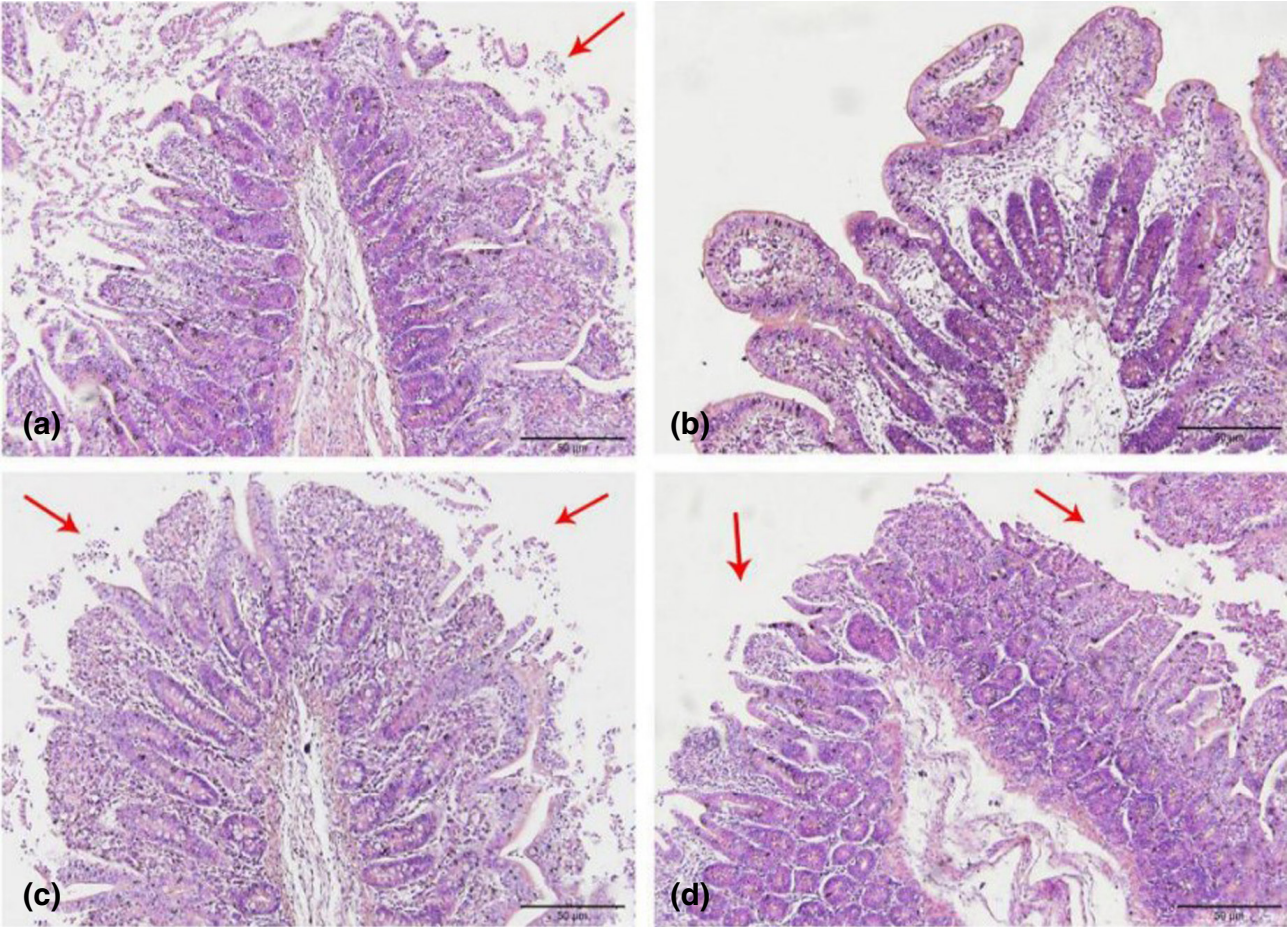
The pathological injury of ileum in each group on 24 h after feeding. (×200). (a–d represents the groups of different CP level 18%, 20%, 22%, 24%, respectively. The arrow points to the villus injury site.)

**FIGURE 1‐8:**
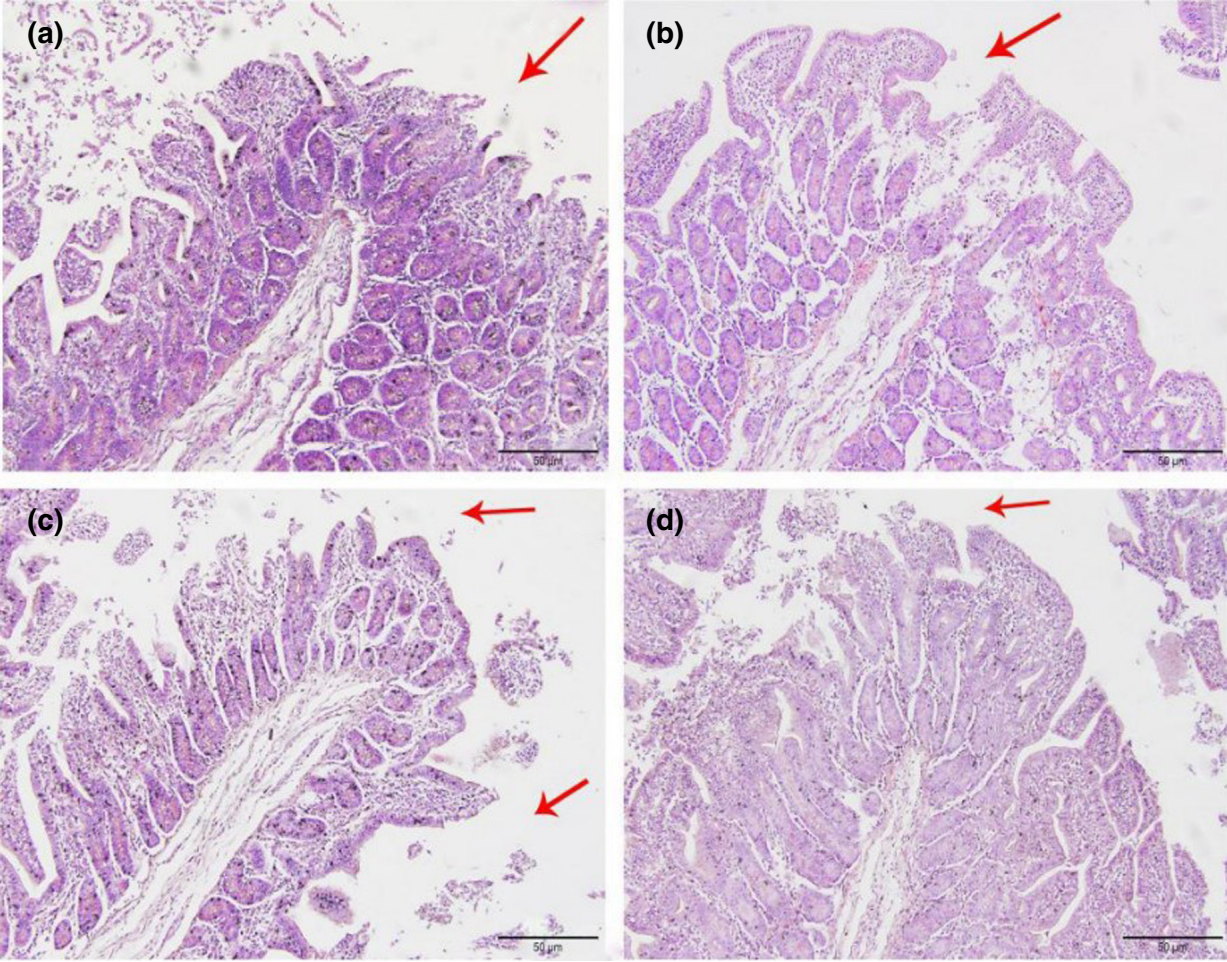
The pathological injury of ileum in each group on 48 h after feeding. (×200). (a–d represents the groups of different CP level 18%, 20%, 22%, 24%, respectively. The arrow points to the villus injury site.)

**FIGURE 1‐9:**
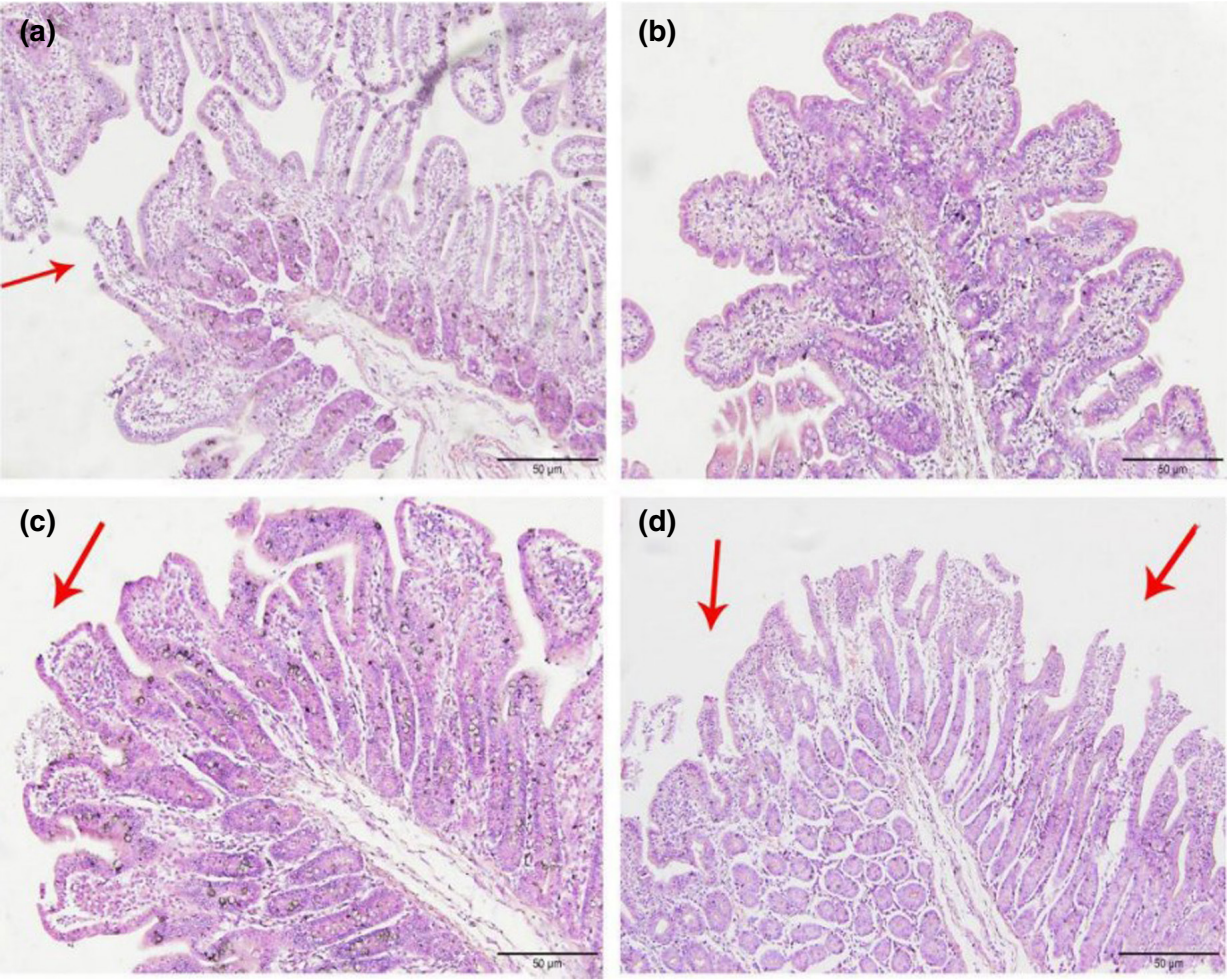
The pathological injury of ileum in each group on 72 h after feeding. (×200). (a–d represents the groups of different CP level 18%, 20%, 22%, 24%, respectively. The arrow points to the villus injury site.)

**FIGURE 1‐10:**
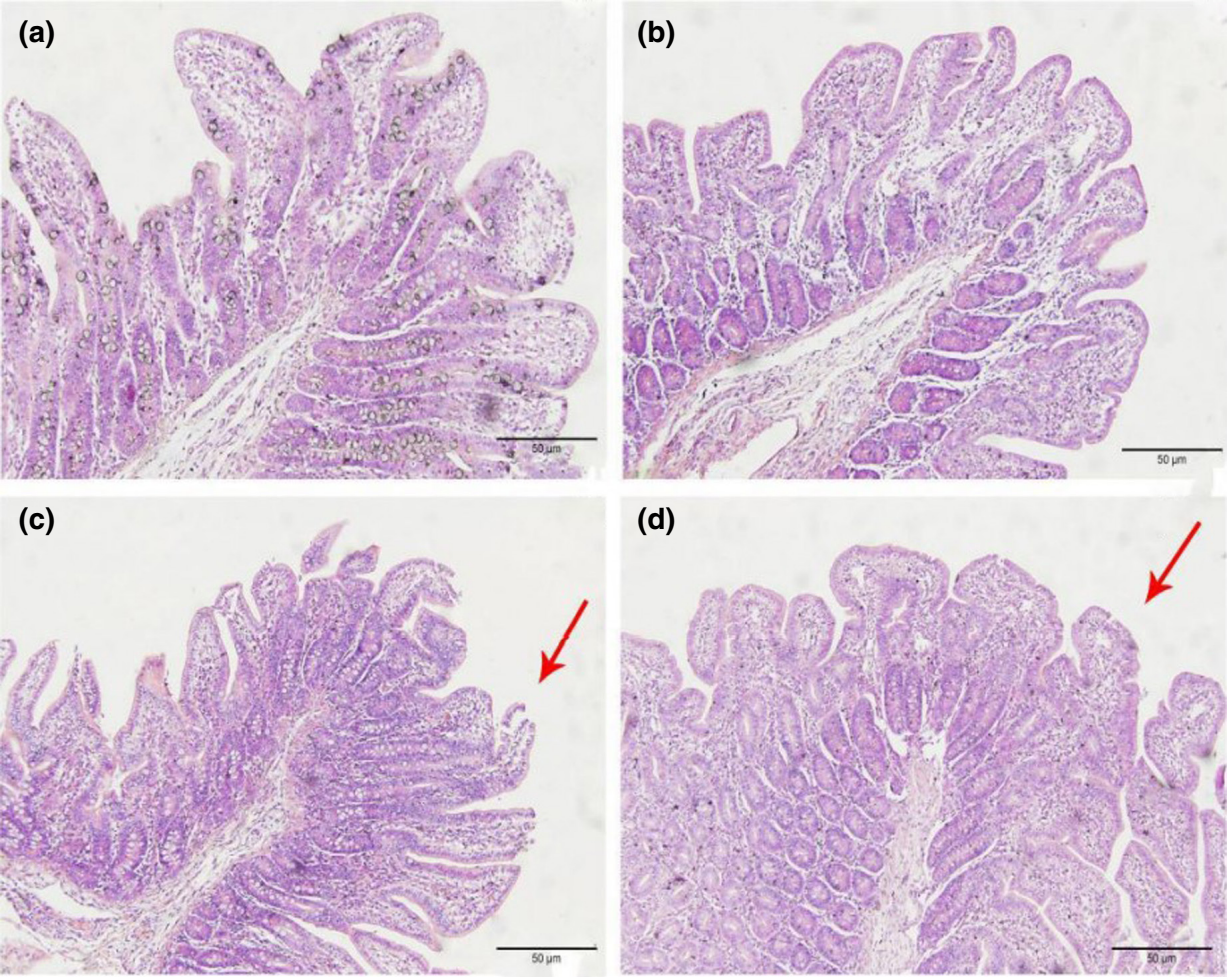
The pathological injury of ileum in each group on 96 h after feeding. (×200). (a–d represents the groups of different CP level 18%, 20%, 22%, 24%, respectively. The arrow points to the villus injury site.)

In summary, CP at the 20% level caused less morphological damage to the jejunum and ileum of piglets. The edge of the villous epithelium was smooth, the length was uniform, and the epithelial cells were neatly arranged. In addition, crypts were also clearly visible.

### 3.2 Effect of CP level on the ultrastructure of the ileal epithelium in weaned piglets

It can be seen in Figure 2‐1 that at 6 and 24 h after feeding, the ileal microvilli of the animals in the 20% CP group were densely and very neatly arranged and were perpendicular to the top of the cells; the cells contained abundant organelles and showed no abnormalities in structure, and there were close connections between the cells. In the other three groups, uneven ileal microvilli, partial dissolution and shedding of microvilli, swelling of the inner and outer mitochondrial membrane structure, widening of intercellular tight junctions, blurred and shortened tight junction structure, vacuolated dissolution of some contents, and ruptures between cells could be observed; this was most severe in the 24% CP group, in which chromatin aggregation was even observed. At 48–96 h, the microstructure of the ileum was unremarkable in the 20% CP group; the microvilli were neatly arranged, organelles were abundant, and the tight junction structure was clearly visible, while in the 22% and 24% CP groups, the tight junction structure was blurred, there were many ruptures between the cells, some organelles were dissolved, and the mitochondria appeared slightly swollen (Figures 2‐2, 2‐3, 2‐4, 2‐5).

**FIGURE 2‐1:**
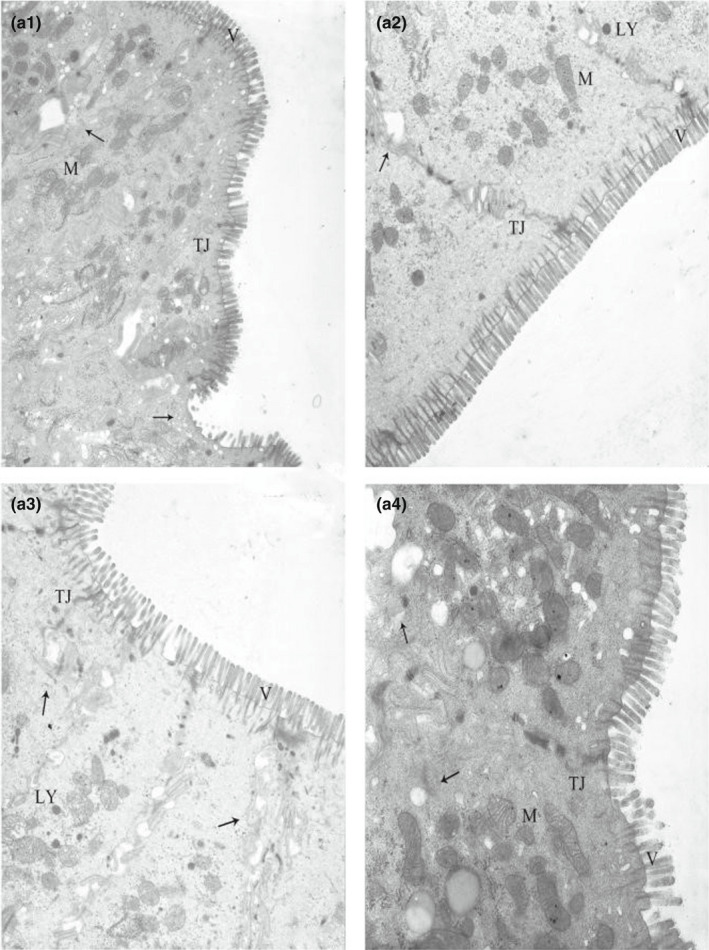
Effect of dietary protein level on the ultrastructure of ileum in weaned piglets fed for 6 h. (×6000). (a1–a4 represents the groups of different CP level 18%, 20%, 22%, 24%, respectively. M: Mitochondrion, V: Microvilli, G: Goblet cell, LY: Lysosome, N: Nucleus, TJ: Tight junction.)

**FIGURE 2‐2:**
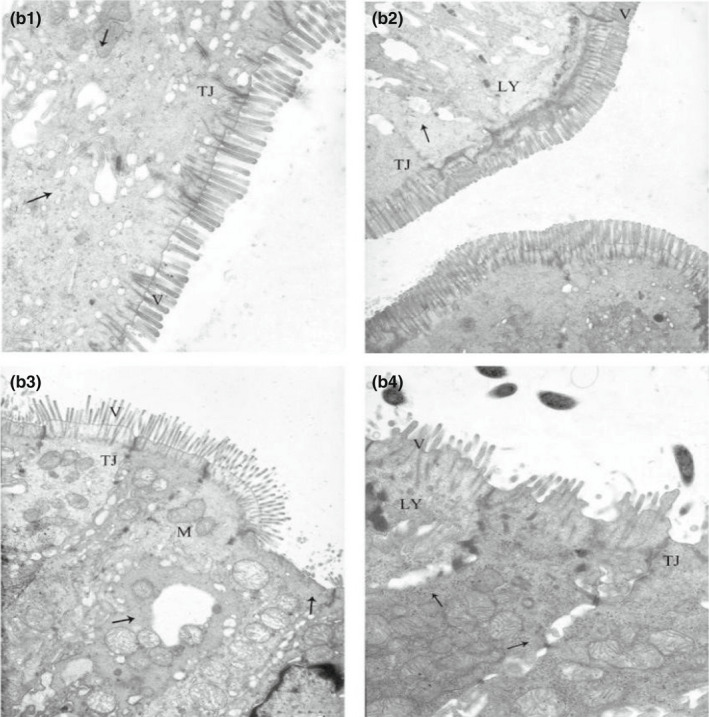
Effect of dietary protein level on the ultrastructure of ileum in weaned piglets fed for 24 h. (×6000). (b1–b4 represents the groups of different CP level 18%, 20%, 22%, 24%, respectively. M: Mitochondrion, V: Microvilli, G: Goblet cell, LY: Lysosome, N: Nucleus, TJ: Tight junction.)

**FIGURE 2‐3:**
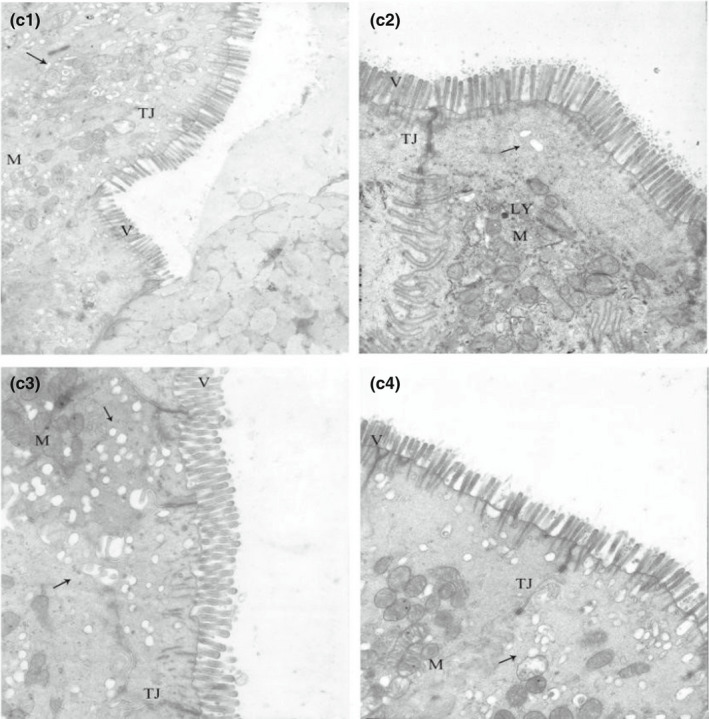
Effect of dietary protein level on the ultrastructure of ileum in weaned piglets fed for 48 h. (×6000). (c1–c4 represents the groups of different CP level 18%, 20%, 22%, 24%, respectively. M: Mitochondrion, V: Microvilli, G: Goblet cell, LY: Lysosome, N: Nucleus, TJ: Tight junction.)

**FIGURE 2‐4:**
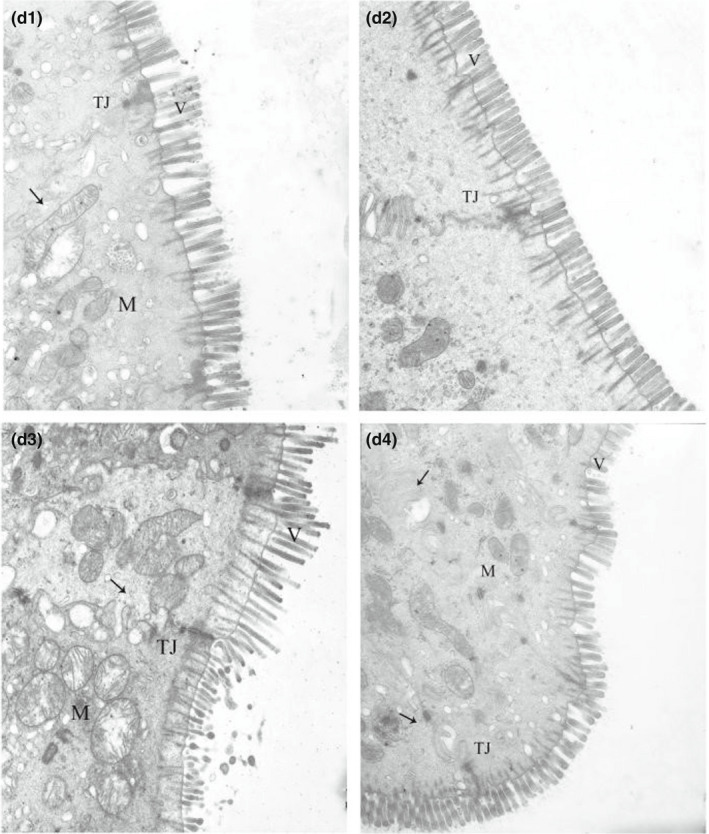
Effect of dietary protein level on the ultrastructure of ileum in weaned piglets fed for 72 h. (×6000). (d1–d4 represents the groups of different CP level 18%, 20%, 22%, 24%, respectively. M: Mitochondrion, V: Microvilli, G: Goblet cell, LY: Lysosome, N: Nucleus, TJ: Tight junction.)

**FIGURE 2‐5:**
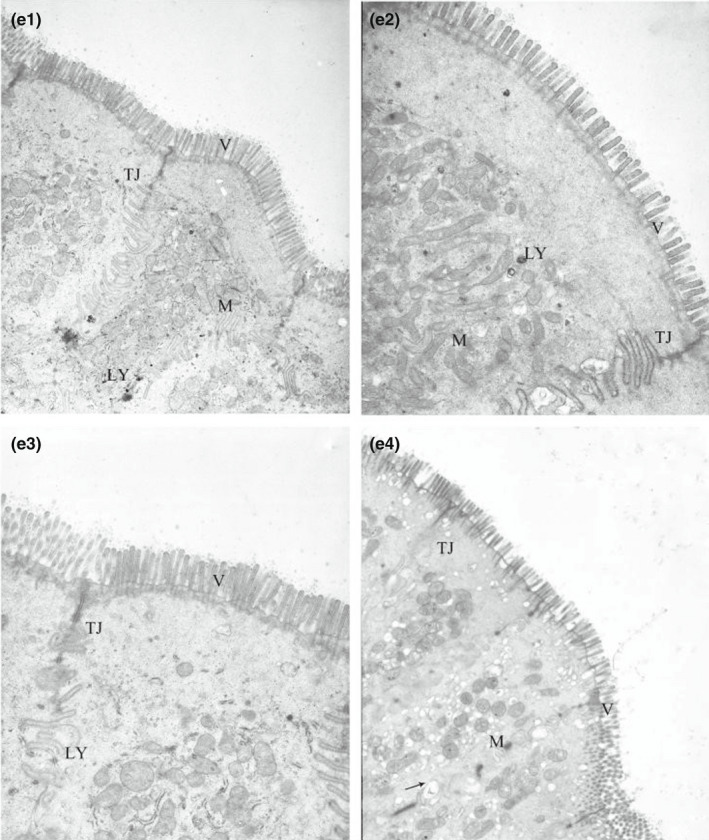
Effect of dietary protein level on the ultrastructure of ileum in weaned piglets fed for 96 h. (×6000). (e1–e4 represents the groups of different CP level 18%, 20%, 22%, 24%, respectively. M: Mitochondrion, V: Microvilli, G: Goblet cell, LY: Lysosome, N: Nucleus, TJ: Tight junction.)

In conclusion, we can see that all but 20% of the CP levels exhibited dissolution and shedding of ileal microvilli, swelling of inner and outer mitochondrial membrane structures, and blurring of tight junctions.

### 3.3 Effect of CP levels on occludin immunohistochemistry in weaned piglets

#### 3.3.1 Immunohistochemical results in the jejunum

From Table 2‐1 and Figures 3‐1, 3‐2, 3‐3, 3‐4, 3‐5, it can be seen that at 6 and 48 h after feeding, the 20% and 22% CP groups displayed strong brown signals at the tops of the jejunal epithelial cells, while the 18% and 24% CP groups showed weak expression and uneven distribution of occludin protein. In addition, the mean optical density of occludin protein in the jejunal epithelial tissues of the 20% CP group was very significantly higher than that of the other three groups (*p* < .01). At 72 h after feeding, strong but heterogeneous expression of occludin protein was observed in the 22% CP group, and the mean optical density of occludin in that group was very significantly higher than that in the 18% and 24% CP groups (*p* < .01); however, there was no significant difference in the mean optical density of occludin between the 22% CP group and the 20% CP group (*p* > .05). At 96 h after feeding, occludin protein was evenly expressed at the tops of jejunal epithelial cells in the 18% and 20% groups, and the mean optical density of occludin in the 18% CP group was very significantly higher than that in the other three groups.

**TABLE 2‐1:** Effect of dietary protein level on average optical density of occludin in jejunum of weaned piglets

Time (h)	Groups			
	18%	20%	22%	24%
6	0.1483 ± 0.0010_C_^C^	0.2353 ± 0.007_A_^A^	0.2007 ± 0.0028_C_^B^	0.1455 ± 0.0012_D_^C^
24	0.1225 ± 0.0005_D_^D^	0.2099 ± 0.0029_B_^A^	0.1641 ± 0.0026_D_^B^	0.1479 ± 0.0019_D_^C^
48	0.1131 ± 0.0021_E_^D^	0.2330 ± 0.0014_A_^A^	0.2240 ± 0.0002_A_^B^	0.1726 ± 0.0026_B_^C^
72	0.1545 ± 0.0033_B_^C^	0.2044 ± 0.0039_B_^Ab^	0.2121 ± 0.0016_B_^A^	0.1952 ± 0.0043_A_^B^
96	0.2605 ± 0.0022_A_^A^	0.2094 ± 0.0014_B_^B^	0.1383 ± 0.0008_E_^D^	0.1629 ± 0.0036_C_^C^

Note

The same letter of shoulder in peer data indicates no significant difference (*p* > .05), the same letter but different case indicates significant difference (*p* < .05), different letters indicate extremely significant difference (*p* < .01), and the same data footmark is consistent with the shoulder marking method.

**FIGURE 3‐1:**
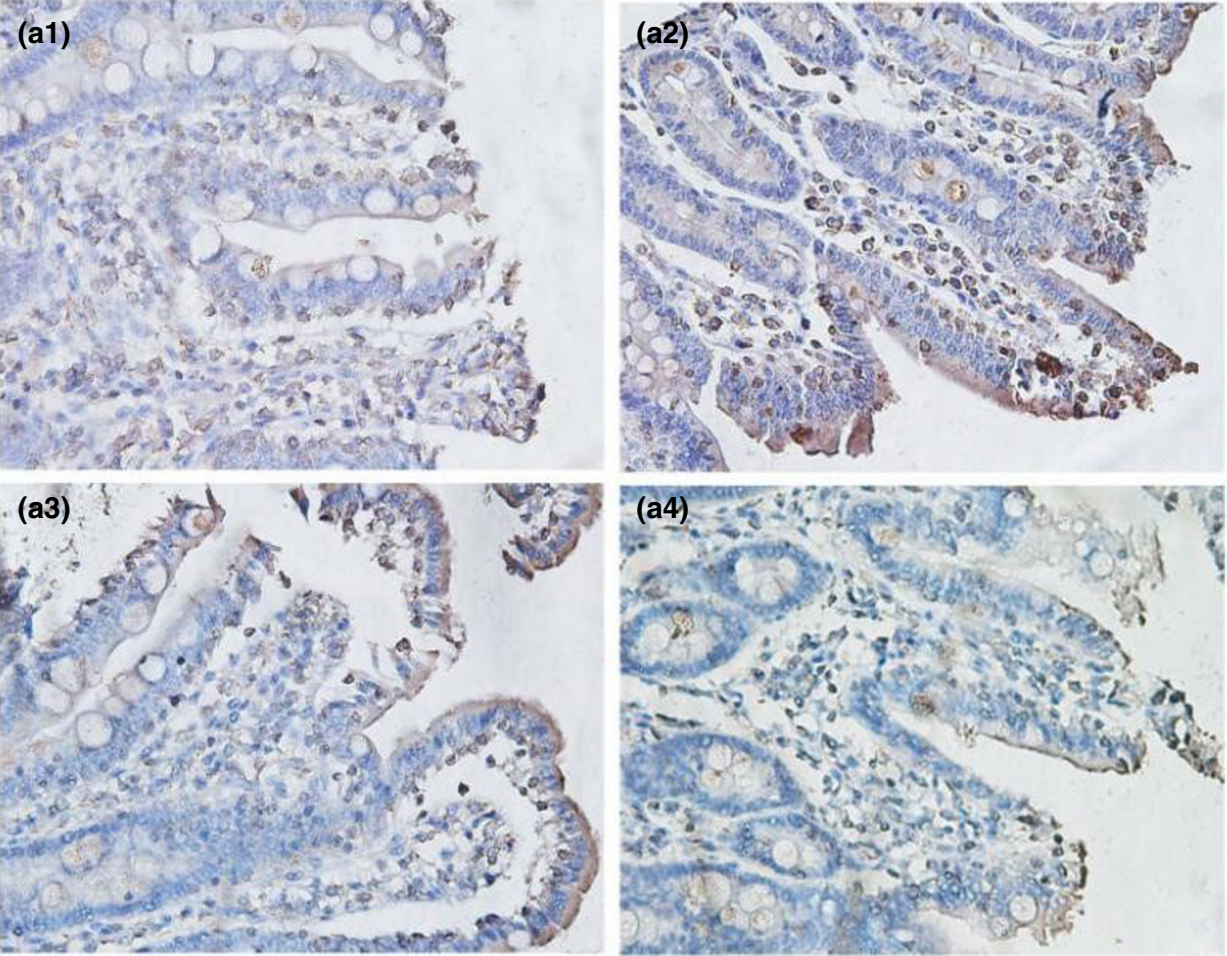
The immunohistochemistry of jejunum in each group on 6 h after feeding. (×400). (a1–a4 represents the groups of different dietary protein level 18%, 20%, 22%, 24%, respectively.)

**FIGURE 3‐2:**
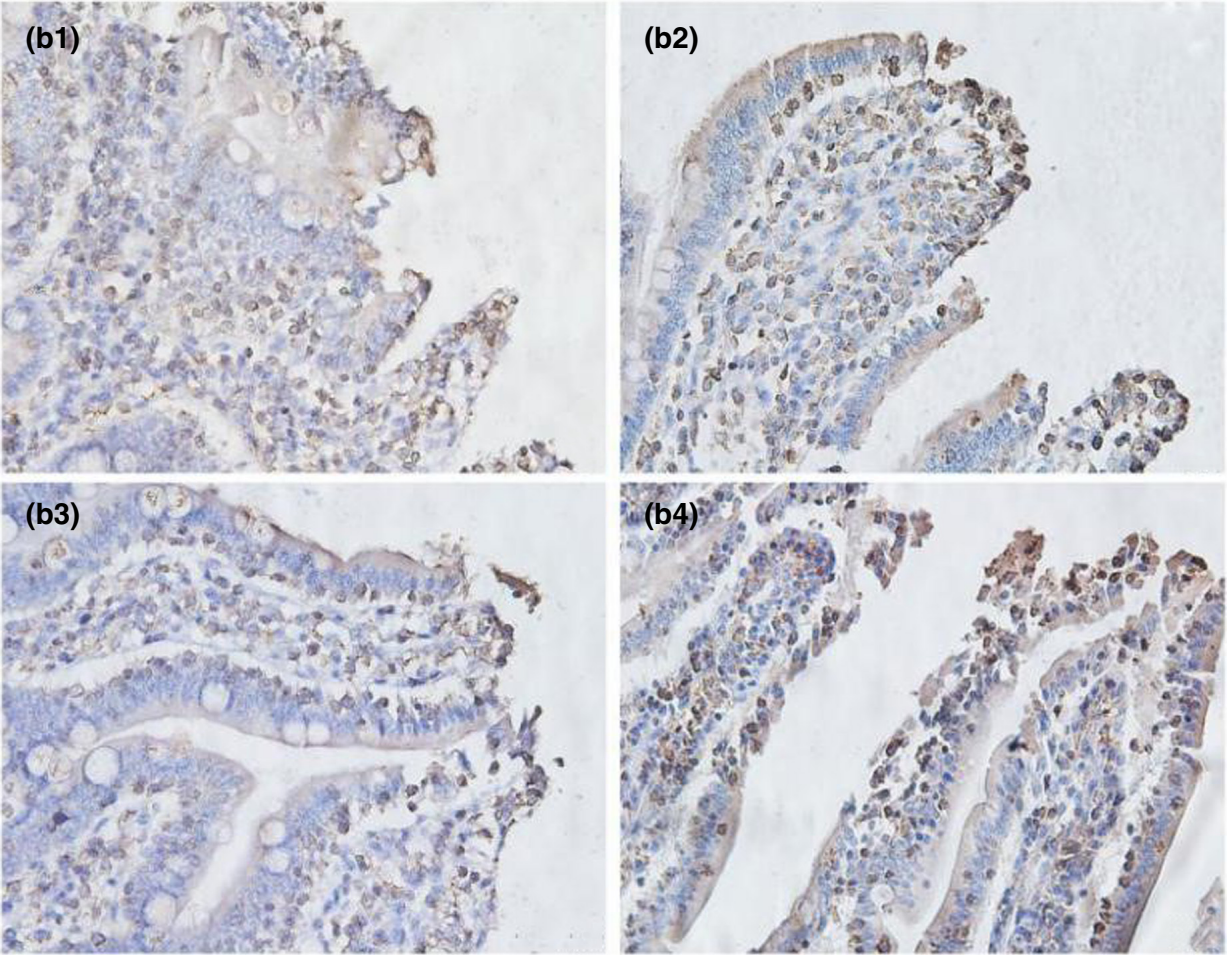
The immunohistochemistry of jejunum in each group on 24 h after feeding. (×400). (b1–b4 represents the groups of different dietary protein level 18%, 20%, 22%, 24%, respectively.)

**FIGURE 3‐3:**
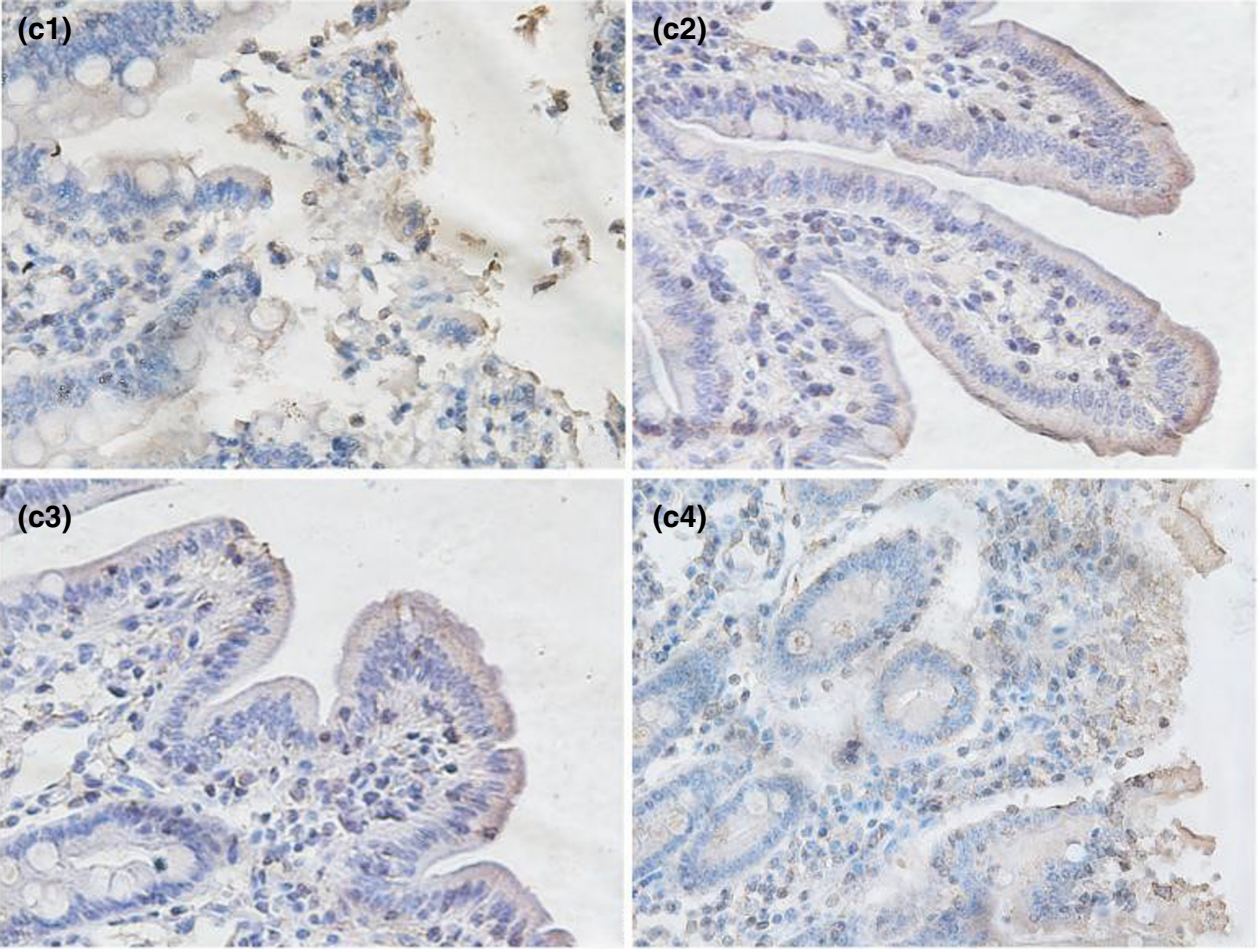
The immunohistochemistry of jejunum in each group on 48 h after feeding. (×400). (c1–c4 represents the groups of different dietary protein level 18%, 20%, 22%, 24%, respectively.)

**FIGURE 3‐4:**
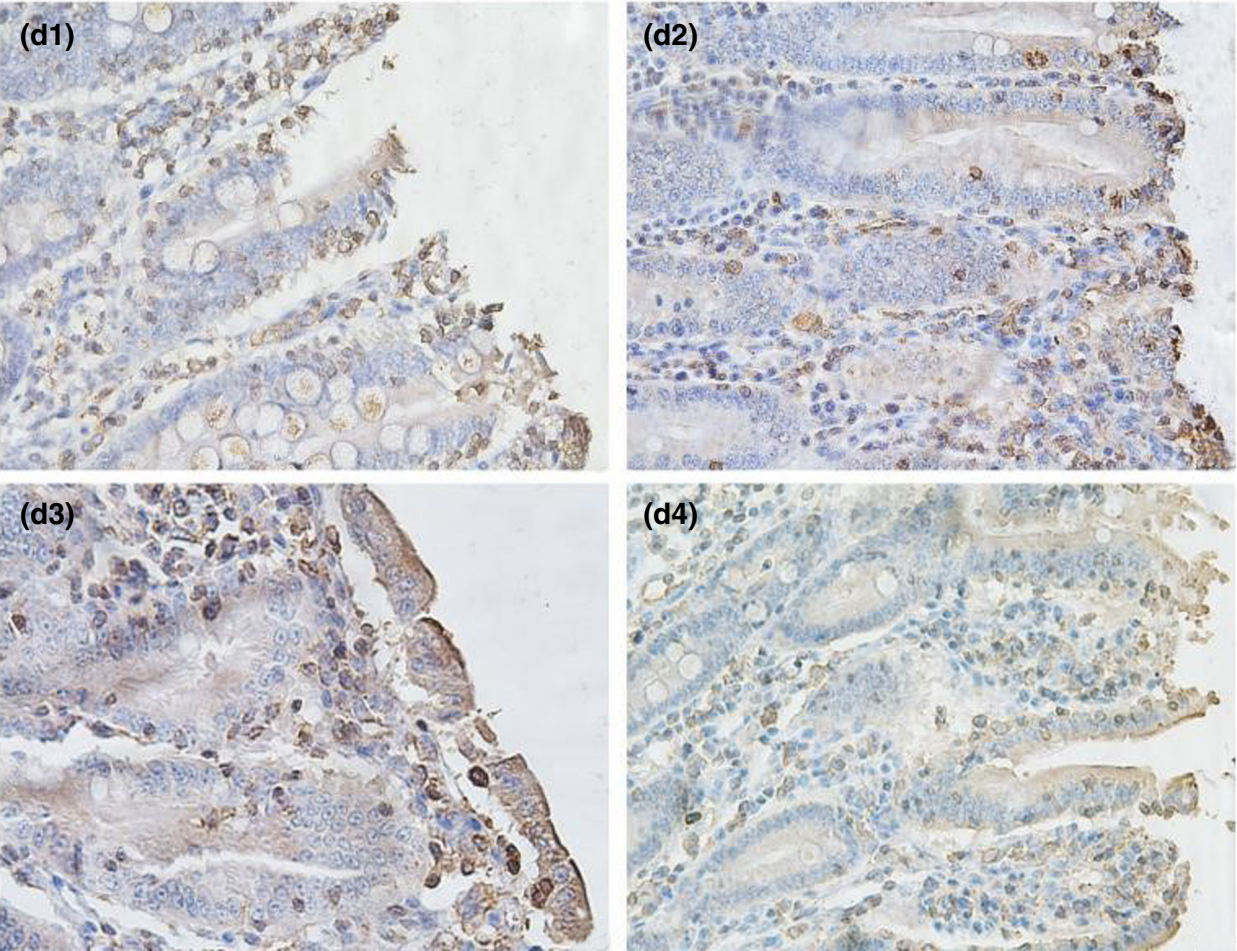
The immunohistochemistry of jejunum in each group on 72 h after feeding. (×400). (d1–d4 represents the groups of different dietary protein level 18%, 20%, 22%, 24%, respectively.)

**FIGURE 3‐5:**
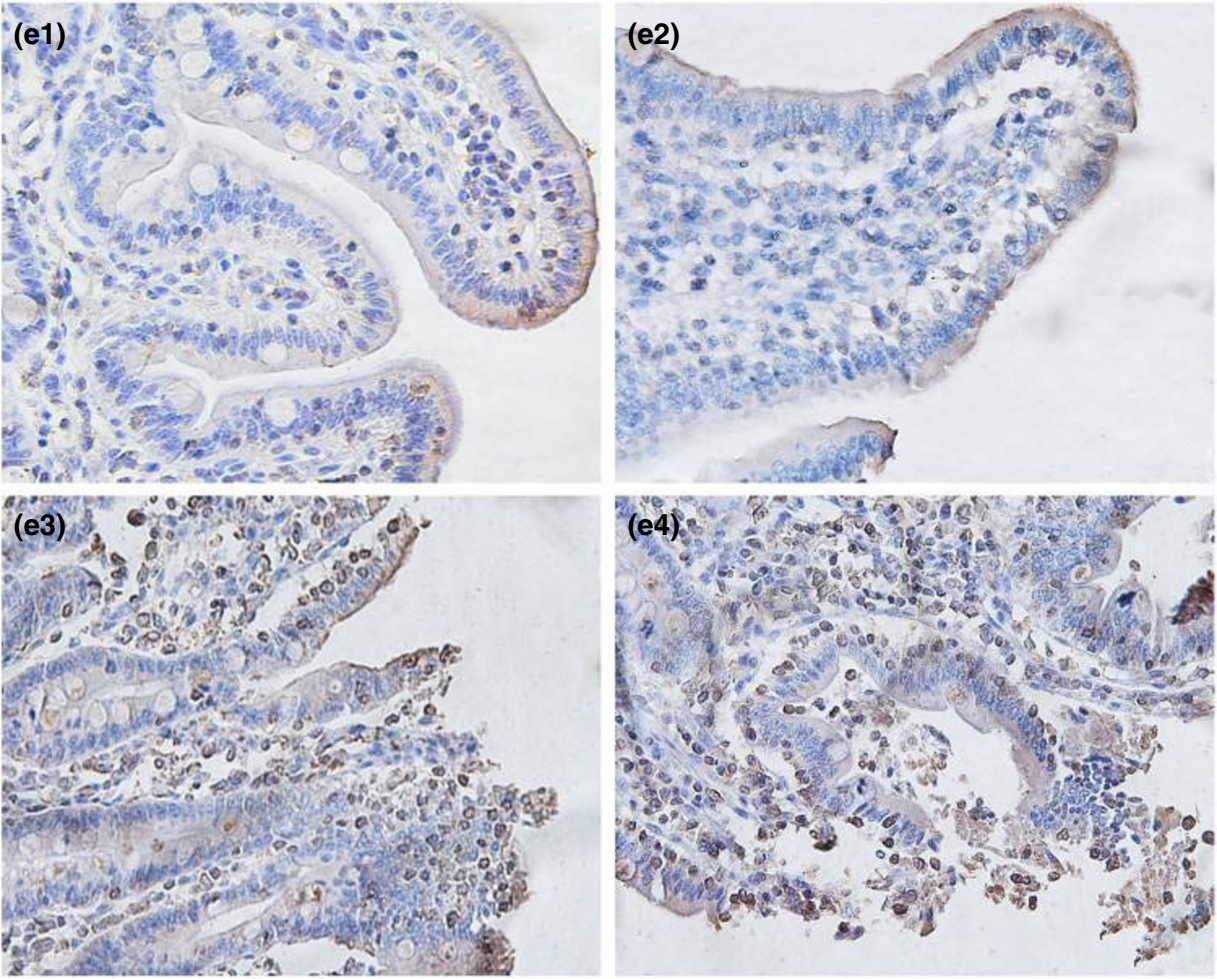
The immunohistochemistry of jejunum in each group on 96 h after feeding. (×400). (e1–e4 represents the groups of different dietary protein level 18%, 20%, 22%, 24%, respectively.)

#### 3.3.2 Immunohistochemical results in the ileum

As shown in Table 2‐2 and Figures 3‐6, 3‐7, 3‐8, 3‐9, 3‐10, strong occludin protein expression was observed at the tops of ileal epithelial cells in the 20% CP group at 6, 24, and 96 h after feeding, and the mean optical density of occludin protein in ileal epithelial tissues in the 20% CP group was very significantly higher than that in the other three groups (*p* < .01). At 48 and 72 h after feeding, occludin protein showed strong expression at the tips of ileal epithelial cells in the 18% and 20% CP groups.

**TABLE 2‐2:** Effect of dietary protein level on average optical density of occludin in ileum of weaned piglets

Time (h)	Groups			
	18%	20%	22%	24%
6	0.1487 ± 0.0012_C_^B^	0.2057 ± 0.0053_b_^A^	0.1440 ± 0.0019_D_^BC^	0.1355 ± 0.0008_D_^c^
24	0.1457 ± 0.0007_C_^B^	0.2188 ± 0.0069_B_^A^	0.1423 ± 0.0021_D_^B^	0.1498 ± 0.0018_C_^B^
48	0.1961 ± 0.0015_B_^A^	0.1703 ± 0.0009_C_^B^	0.1519 ± 0.0025_C_^C^	0.1718 ± 0.0012_B_^B^
72	0.2021 ± 0.0069_B_^A^	0.2091 ± 0.0021_Bb_^A^	0.1641 ± 0.0028_B_^B^	0.1528 ± 0.0019_C_^b^
96	0.2146 ± 0.0016_A_^B^	0.2367 ± 0.0010_A_^A^	0.1786 ± 0.0029_A_^D^	0.1870 ± 0.0027_A_^C^

Note

The same letter of shoulder in peer data indicates no significant difference (*p* > .05), the same letter but different case indicates significant difference (*p* < .05), different letters indicate extremely significant difference (*p* < .01), and the same data footmark is consistent with the shoulder marking method.

**FIGURE 3‐6:**
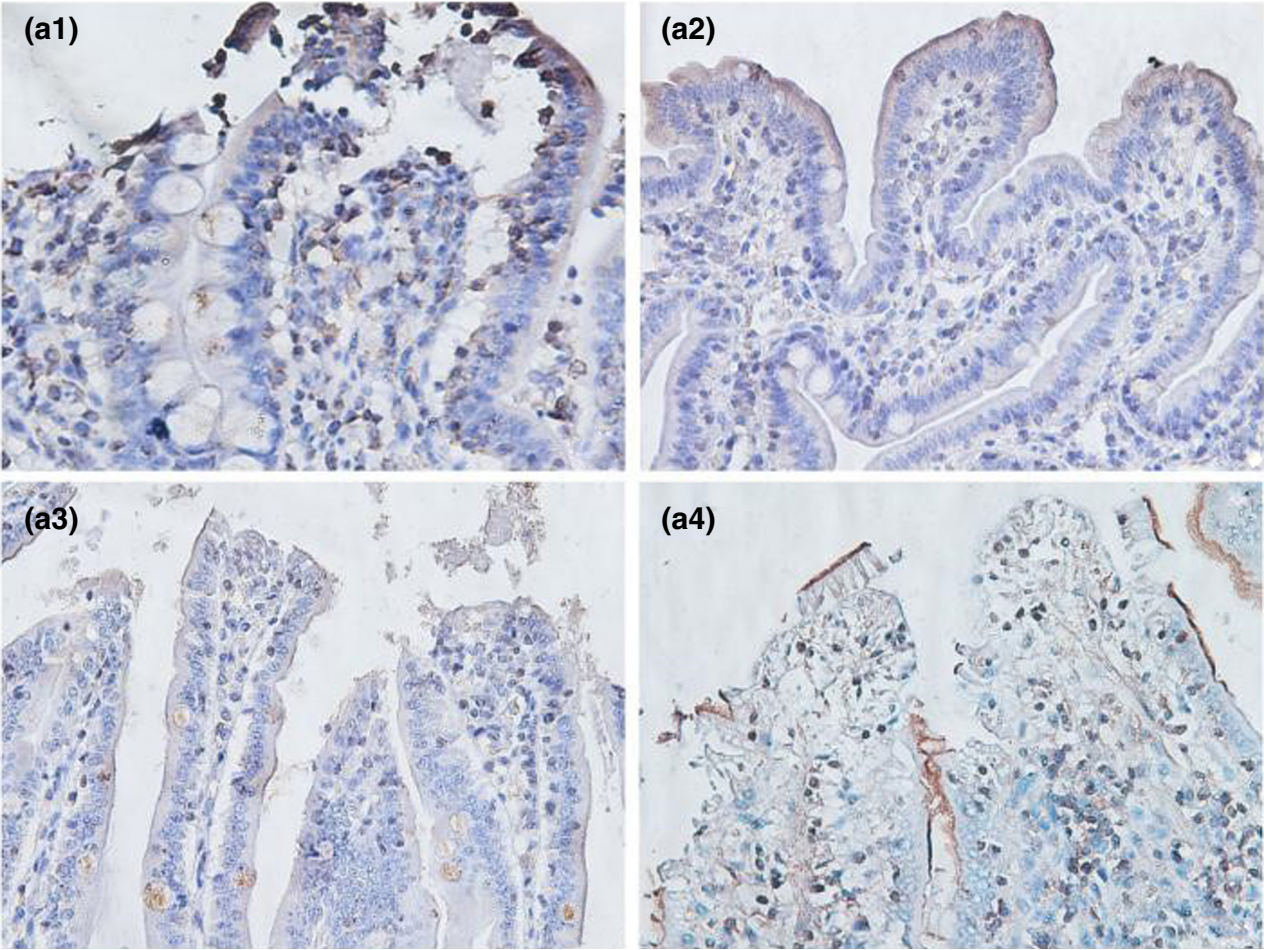
The immunohistochemistry of ileum in each group on 6 h after feeding. (×400). (a1–a4 represents the groups of different dietary protein level 18%, 20%, 22%, 24%, respectively.)

**FIGURE 3‐7:**
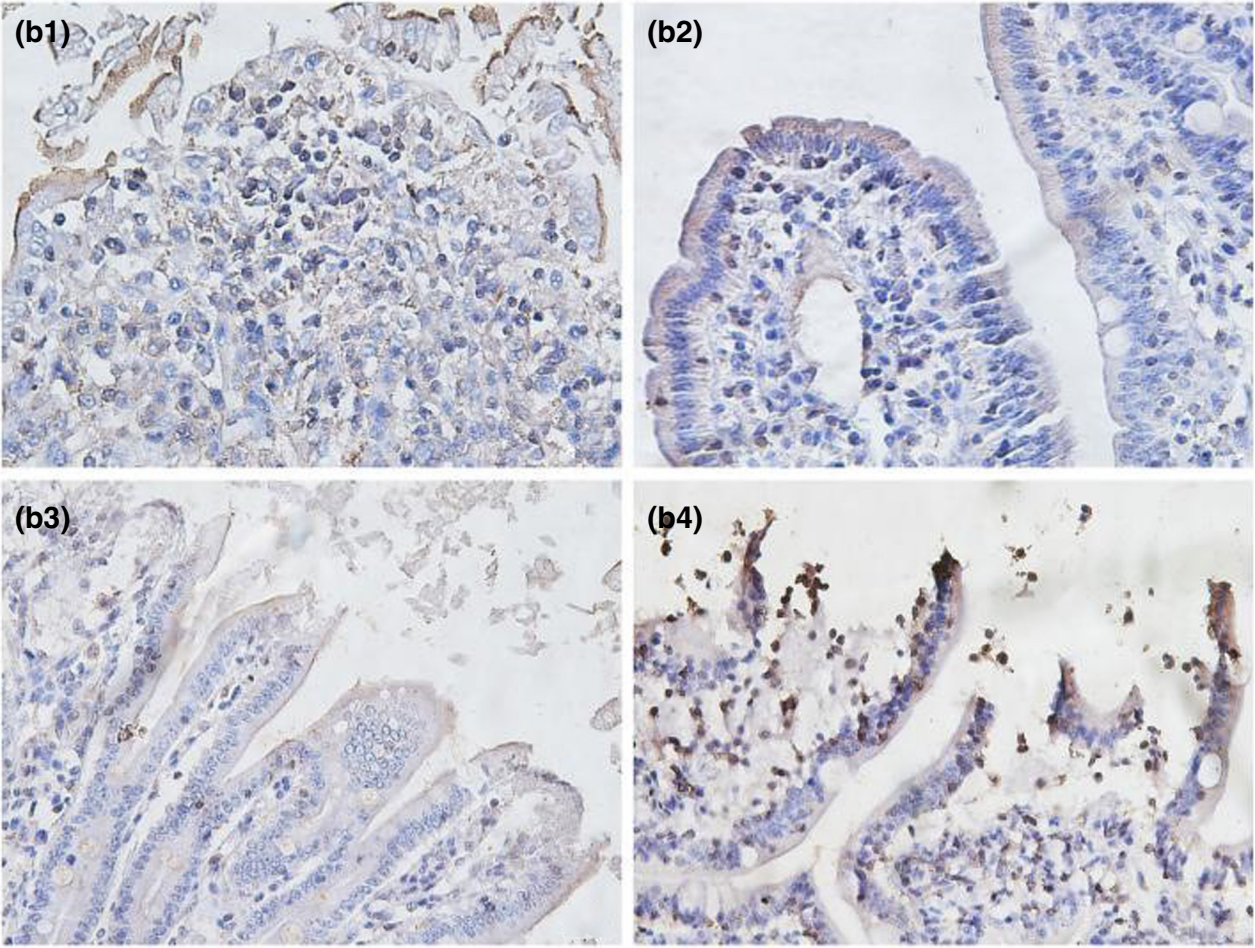
The immunohistochemistry of ileum in each group on 24 h after feeding. (×400). (b1–b4 represents the groups of different dietary protein level 18%, 20%, 22%, 24%, respectively.)

**FIGURE 3‐8:**
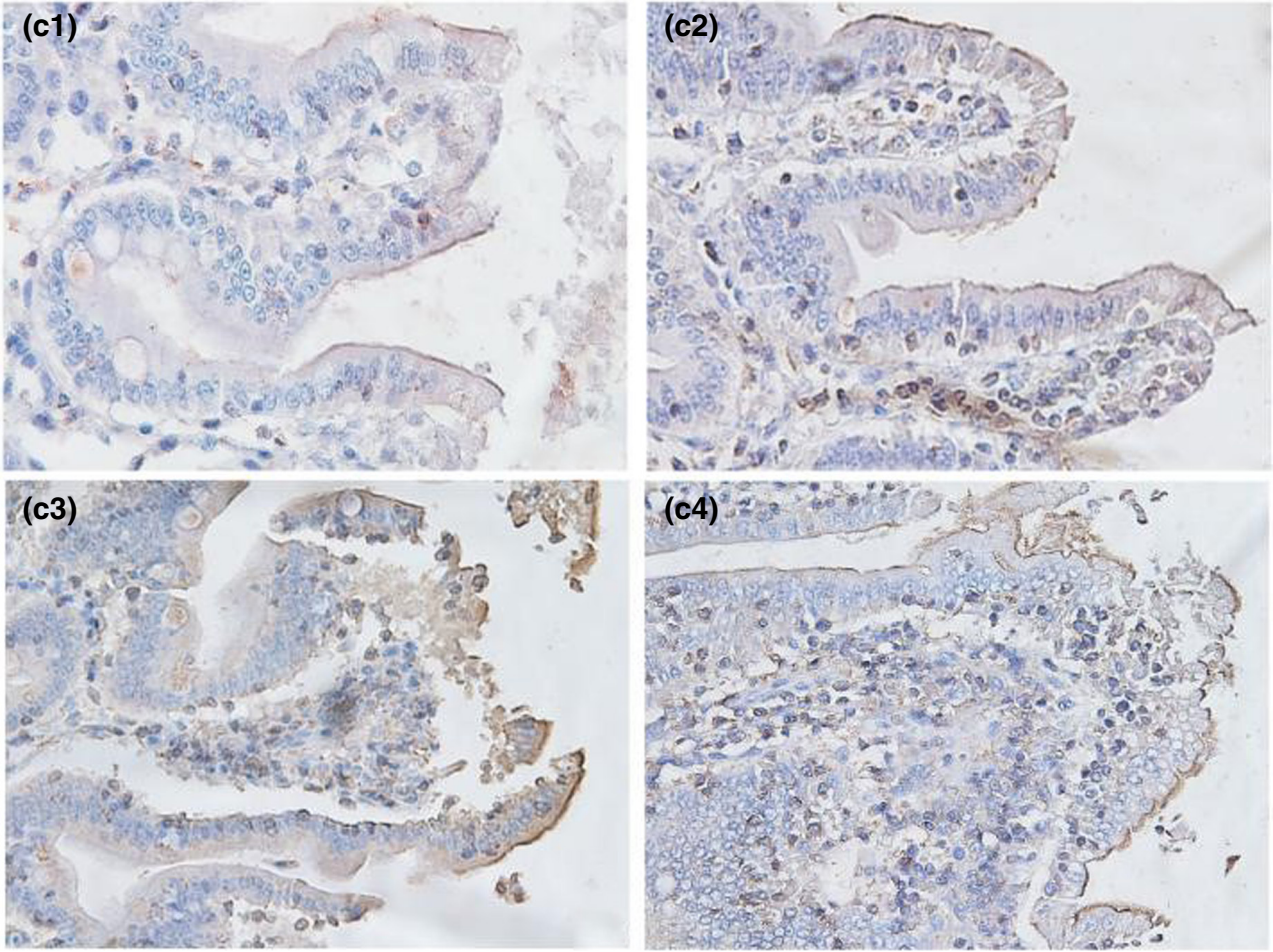
The immunohistochemistry of ileum in each group on 48 h after feeding. (×400). (c1–c4 represents the groups of different dietary protein level 18%, 20%, 22%, 24%, respectively.)

**FIGURE 3‐9:**
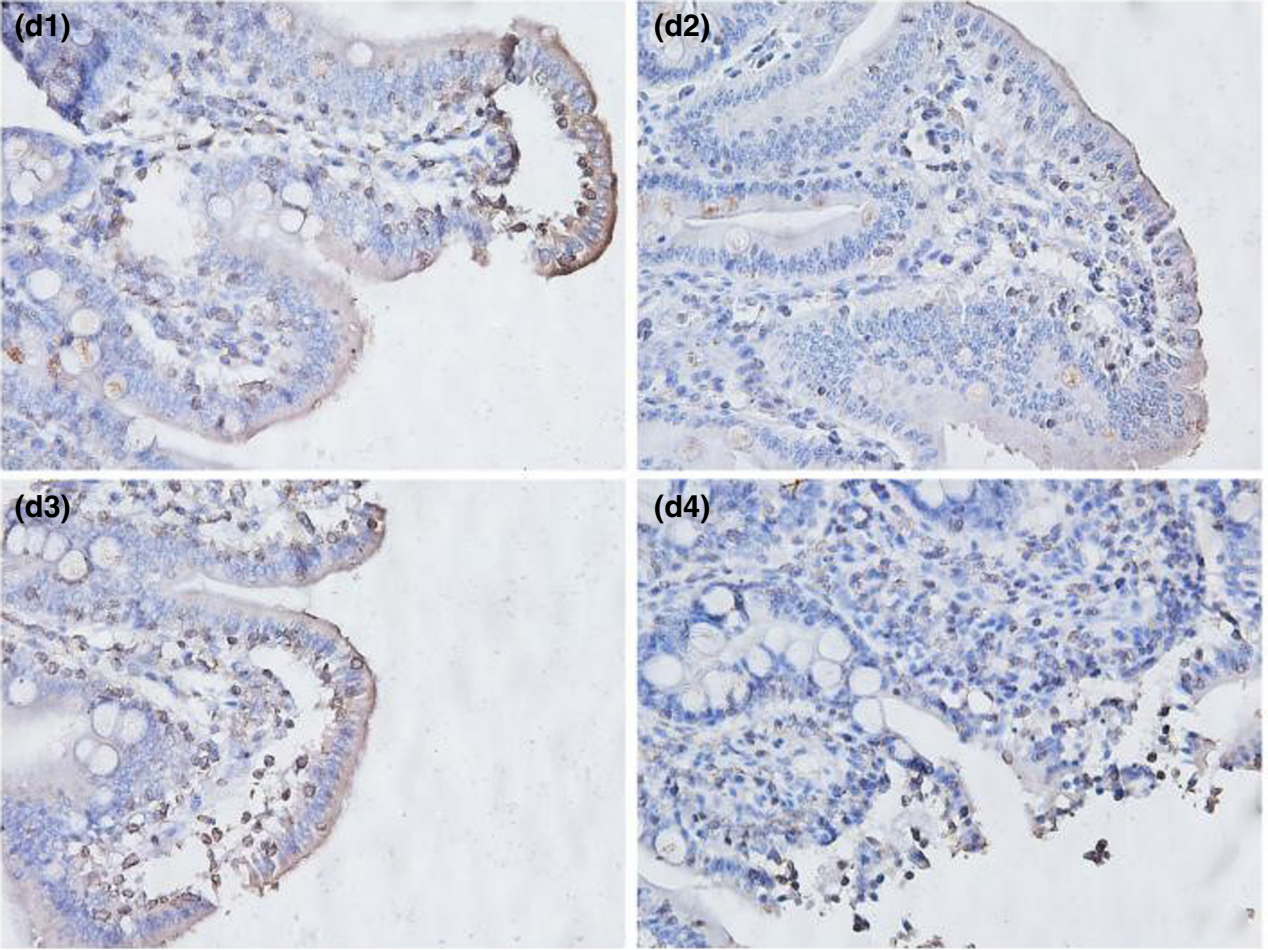
The immunohistochemistry of ileum in each group on 72 h after feeding. (×400). (d1–d4 represents the groups of different dietary protein level 18%, 20%, 22%, 24%, respectively.)

**FIGURE 3‐10:**
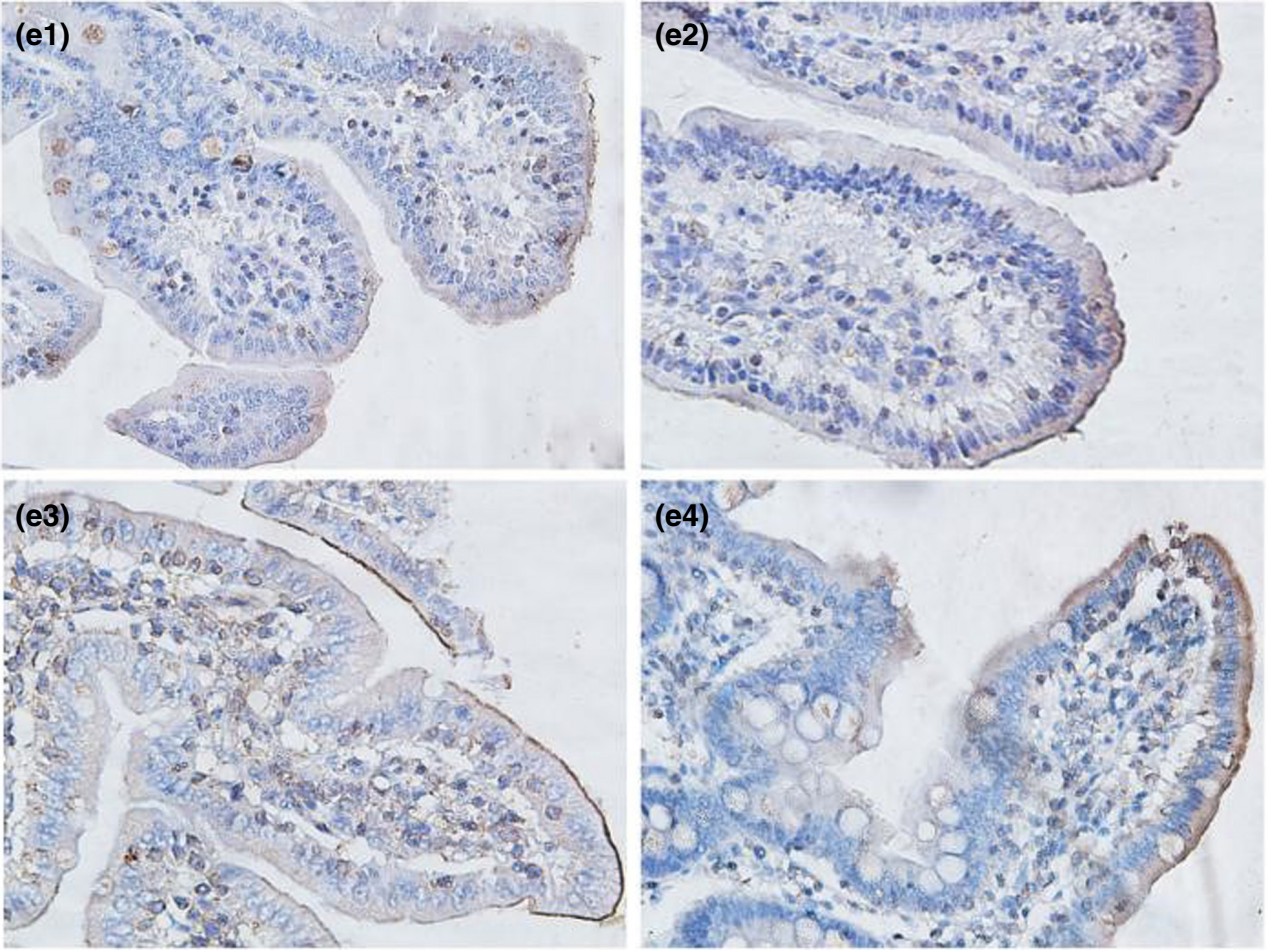
The immunohistochemistry of ileum in each group on 96 h after feeding. (×400). (e1–e4 represents the groups of different dietary protein level 18%, 20%, 22%, 24%, respectively.)

In summary, 20% level of CP can strongly express occludin protein in both jejunal and ileal epithelial tissues.

### 3.4 Effect of CP level on relative occludin mRNA expression in weaned piglets

#### 3.4.1 Effect on occludin mRNA expression in the jejunum

As shown in Table 2‐3, the relative expression of occludin mRNA in the jejunum at 6 and 24 h after feeding was highest in the 20% and 22% CP groups; the expression in those groups was very significantly higher than that in the 18% and 24% groups (*p* < .01), in which the relative expression of occludin mRNA in the jejunum was low. At 48, 72, and 76 h after feeding, the relative expression of occludin mRNA was highest in the 20% CP group; at these times, the relative expression of occludin in this group was very significantly higher or significantly higher than that in the 22% CP group (*p* < .01 or *p* < .05).

**TABLE 2‐3:** Effect of dietary protein level on relative changes of occludin mRNA in jejunum of weaned piglets

Time (h)	Groups			
	18%	20%	22%	24%
6	0.742 ± 0.026^B^_B_	1.826 ± 0.102^A^_A_	1.490 ± 0.123^a^_A_	0.422 ± 0.039^b^_b_
24	0.506 ± 0.057^B^_C_	0.954 ± 0.022^A^_B_	1.062 ± 0.133^A^_b_	0.537 ± 0.060^B^_BC_
48	0.928 ± 0.056^AB^_A_	1.176 ± 0.148^A^_B_	0.673 ± 0.078^aB^_BC_	0.633 ± 0.140^B^_aB_
72	0.812 ± 0.016^a^_aB_	1.167 ± 0.121^A^_B_	0.432 ± 0.102^B^_C_	0.912 ± 0.029^a^_A_
96	0.842 ± 0.013^B^_AB_	1.230 ± 0.161^A^_B_	0.407 ± 0.016^C^_c_	0.520 ± 0.035^bC^_BC_

Note

The same letter of shoulder in peer data indicates no significant difference (*p* > .05), the same letter but different case indicates significant difference (*p* < .05), different letters indicate extremely significant difference (*p* < .01), and the same data footmark is consistent with the shoulder marking method.

#### 3.4.2 Effect on occludin mRNA expression in the ileum

From Table 2‐4, it can be seen that the relative expression of occludin mRNA in the ileum at 6, 24, and 72 h after feeding was highest in the 20% CP group; at those times, it was very significantly higher than that of the 22% CP group (*p* < .01). After 48 and 96 h of feeding, the relative expression of occludin mRNA was highest in the 18% CP group; in that group, it was very significantly higher than in the 22% CP group (*p* < .01). Among all of the experimental groups, occludin mRNA expression was lowest in the 22% CP group.

**TABLE 2‐4:** Effect of Dietary protein level on relative changes of occludin mRNA in ileum of weaned piglets

Time (h)	Groups			
	18%	20%	22%	24%
6	0.769 ± 0.059^ab^_C_	1.089 ± 0.124^A^_Ab_	0.547 ± 0.019^B^_B_	0.852 ± 0.030^ab^_aBC_
24	0.711 ± 0.128^B^_C_	1.431 ± 0.098^A^_AB_	0.499 ± 0.049^B^_B_	0.675 ± 0.091^B^_bCD_
48	1.467 ± 0.128^A^_AB_	1.114 ± 0.105^AB^_A_	0.515 ± 0.075^b^_B_	1.113 ± 0.199^AB^_A_
72	1.121 ± 0.179^A^_bc_	1.280 ± 0.108^A^_A_	0.489 ± 0.006^B^_B_	0.542 ± 0.004^B^_cD_
96	1.742 ± 0.085^A^_A_	1.253 ± 0.173^b^_A_	0.802 ± 0.026^C^_A_	0.998 ± 0.082^BC^_AB_

Note

The same letter of shoulder in peer data indicates no significant difference (*p* > .05), the same letter but different case indicates significant difference (*p* < .05), different letters indicate extremely significant difference (*p* < .01), and the same data footmark is consistent with the shoulder marking method.

Above all, the expression of occludin mRNA in the jejunum and ileum is highest at 20% CP group.

### 3.5 Effect of CP level on occludin protein expression in weaned piglets

#### 3.5.1 Effect on expression of the tight junction protein occludin in the jejunum

As shown in Table 2‐5 and Figure 4‐1, the expression level of the tight junction protein occludin in the jejunum at 6–48 h after feeding was highest in the 20% CP group; in that group, it was very significantly or significantly higher than the levels in the other three groups (*p* < .01 or *p* < .05). At 72 h after feeding, the expression of occludin was highest in the 22% CP group; in that group, the level was very significantly higher than the levels in the other three groups (*p* < .01). However, at 24–72 h after feeding, the expression of occludin was lowest in the 18% CP group; in that group, it was very significantly lower than the levels in the other three groups (*p* < .01).

**TABLE 2‐5:** Effect of dietary protein level on occludin expression in jejunum of weaned piglets

Time (h)	Groups			
	18%	20%	22%	24%
6	0.3497 ± 0.0021_C_^C^	0.7036 ± 0.0048_AB_^A^	0.6313 ± 0.0265_B_^B^	0.3603 ± 0.0194_D_^C^
24	0.2739 ± 0.0046_D_^D^	0.5989 ± 0.0270_bC_^A^	0.5180 ± 0.0257_C_^B^	0.4431 ± 0.0120_C_^C^
48	0.2798 ± 0.0034_D_^C^	0.7351 ± 0.0276_A_^A^	0.6878 ± 0.0158_A_^a^	0.5017 ± 0.0145_B_^B^
72	0.4219 ± 0.0027_B_^C^	0.6433 ± 0.0280_bc_^b^	0.7113 ± 0.0137_A_^A^	0.6019 ± 0.0133_A_^B^
96	0.8729 ± 0.0193_A_^A^	0.5867 ± 0.0270_C_^B^	0.3744 ± 0.0187_D_^D^	0.5201 ± 0.0183_B_^C^

Note

The same letter of shoulder in peer data indicates no significant difference (*p* > .05), the same letter but different case indicates significant difference (*p* < .05), different letters indicate extremely significant difference (*p* < .01), and the same data footmark is consistent with the shoulder marking method.

**FIGURE 4‐1:**
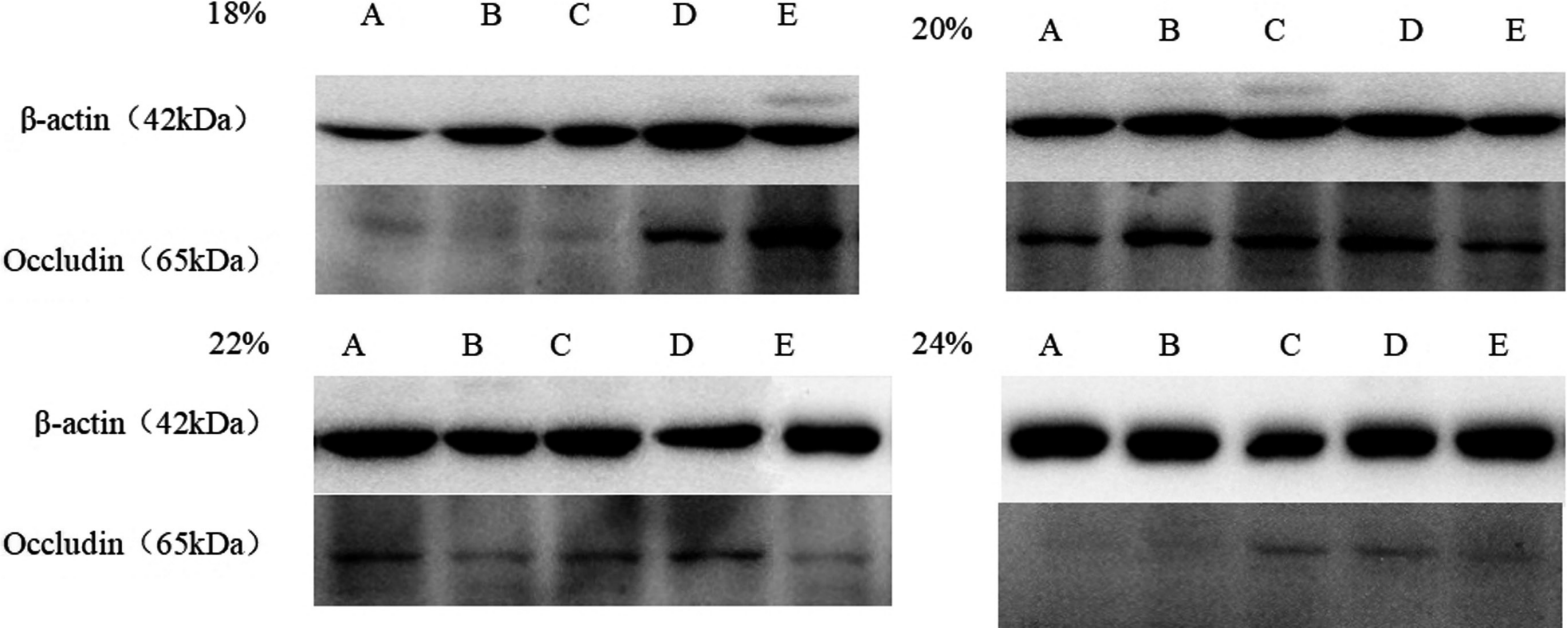
Effects of dietary protein level on jejunum occludin in weaned piglets. A–E represents the time point of 6, 24, 48, 72, 96 h.)

#### 3.5.2 Expression of the tight junction protein occludin in the ileum

As shown in Table 2‐6 and Figure 4‐2, the expression level of the ileal tight junction protein occludin at 6, 24, and 72 h after feeding was highest in the 20% CP group; in that group, the level was very significantly higher than the levels in the other three groups (*p* < .01). Among the 18%, 22%, and 24% CP groups, the expression level in the 24% CP group was the lowest (except at 24 h). After 48 and 96 h of feeding, the expression of occludin was highest in the 18% CP group; in that group, it was very significantly higher than in the 20% and 22% CP groups (*p* < .01).

**TABLE 2‐6:** Effect of dietary protein level on occludin expression in ileum of weaned piglets

Time (h)	Groups			
	18%	20%	22%	24%
6	0.4159 ± 0.0173_D_^B^	0.8860 ± 0.0166_A_^A^	0.4157 ± 0.0215_C_^B^	0.4090 ± 0.0200_c_^B^
24	0.4116 ± 0.0180_D_^C^	0.7549 ± 0.0188_C_^A^	0.4226 ± 0.0207_C_^C^	0.4775 ± 0.0217_B_^B^
48	0.6036 ± 0.0233_C_^A^	0.4248 ± 0.0187_D_^B^	0.4737 ± 0.0130_B_^b^	0.6051 ± 0.0201_a_^A^
72	0.6859 ± 0.0187_B_^B^	0.7396 ± 0.0184_C_^A^	0.4720 ± 0.0187_B_^C^	0.4612 ± 0.0199_C_^C^
96	0.9645 ± 0.0158_A_^A^	0.8322 ± 0.0219_B_^B^	0.5852 ± 0.0179_A_^D^	0.6494 ± 0.0209_A_^C^

Note

The same letter of shoulder in peer data indicates no significant difference (*p* > .05), the same letter but different case indicates significant difference (*p* < .05), different letters indicate extremely significant difference (*p* < .01), and the same data footmark is consistent with the shoulder marking method.

**FIGURE 4‐2:**
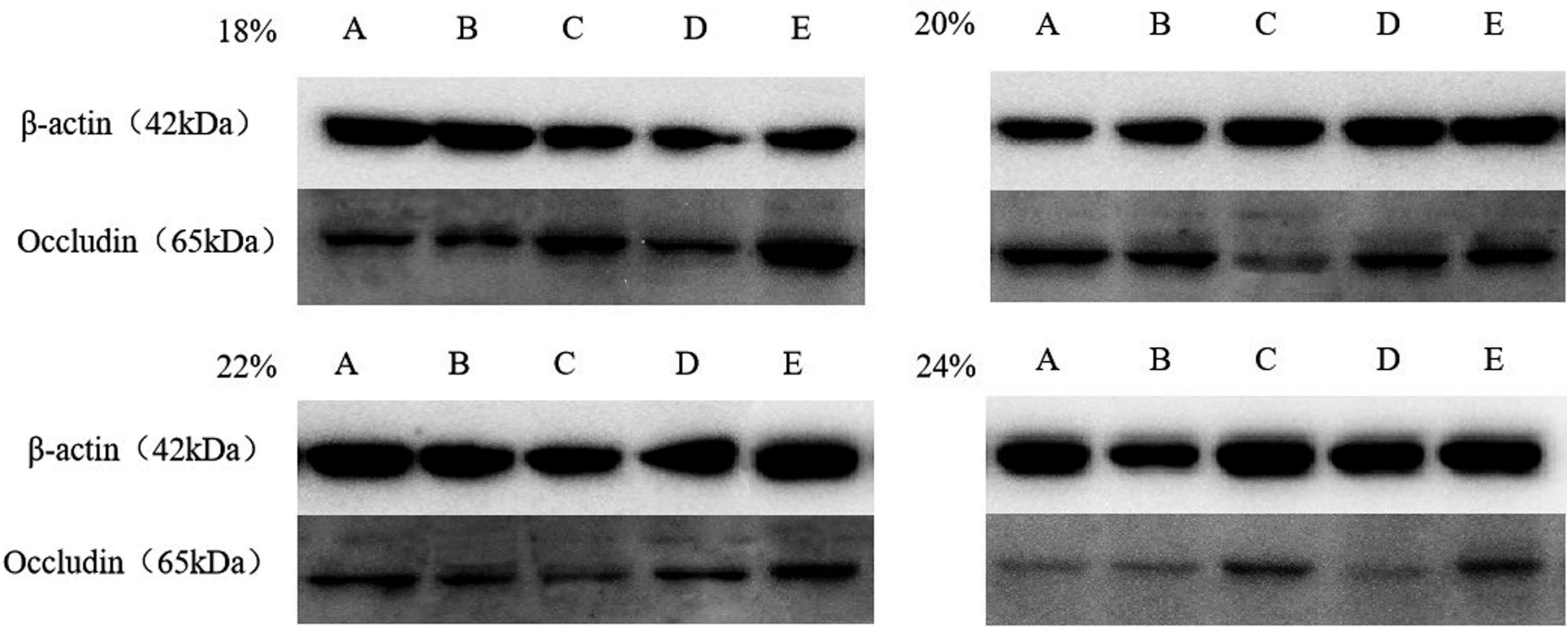
Effects of dietary protein level on ileum occludin in weaned piglets. (A–E represents the time point of 6, 24, 48, 72, 96 h.)

In conclusion, in the jejunum and ileum, the expression of occludin protein was higher in 18% CP after 96 h of feeding and in 20% CP than in the other groups.

### 3.6 Analysis of microbial community diversity and composition

#### 3.6.1 Total DNA extraction of intestinal digesta bacteria and sequencing depth of samples

After extraction of total DNA from intestinal digesta, the OD260/280 of the extracted DNA measured using the Nanodrop 2000 was between 1.8 and 2.0, and the DNA concentration was approximately 100–300 ng/μl.

On the Illumina HiSeq sequencing platform, the 16S rDNA V4 region of the microorganisms present in the intestinal digesta was sequenced at both ends; a total of 5,065,900 high‐quality sequences were obtained, resulting in 30,583 OTUs with a sequence length of approximately 420 bp after clustering with 97% consistency. From Tables 3‐1 and 3‐2, it can be seen that the number of OTUs in the intestines of the animals in the 22% CP group was very significantly higher than the number of OTUs in the intestines of the other three groups (*p* < .01) and that the number of OTUs in the colon at each time point in each group was higher than the corresponding number of ileal OTUs. As shown in Figure 5‐1, the dilution curve of each sample eventually tends to be flat. From Figure 5‐2, it can be seen that the boxplot position tends to be flat and that the confidence interval is decreasing.

**TABLE 3‐1:** Microbial sequencing of ileal chyme

Parameter	Groups			
	18% CP	20% CP	22% CP	24% CP
Sequence number				
6 h	73,744 ± 4678	76,348 ± 1716	79,596 ± 3246	74,657 ± 4873
24 h	81,964 ± 3665	73,473 ± 4864	79,463 ± 3376	66,862 ± 1130
72 h	69,129 ± 2399	70,810 ± 1405	81,384 ± 4071	64,822 ± 3943
OUT				
6 h	357 ± 46^ab^	282 ± 28^BC^	451 ± 42^A^	251 ± 26^C^
24 h	180 ± 17^C^	212 ± 33^C^	337 ± 25^B^	417 ± 24^A^
72 h	228 ± 28^Bb^	192 ± 15^b^	894 ± 33^A^	262 ± 38^B^

Note

Peer data shoulder letter with the same case means significant (*p* < .05), letter with different case means extremely significant (*p* < .01).

**TABLE 3‐2:** Microbial sequencing of colonic chyme

Parameter	Groups			
	18% CP	20% CP	22% CP	24% CP
Sequence number				
6 h	63,759 ± 2071	65,527 ± 3735	79,684 ± 4257	58,106 ± 4027
24 h	68,552 ± 4521	57,736 ± 3723	81,266 ± 4691	58,019 ± 2137
72 h	57,272 ± 4103	62,816 ± 3529	85,381 ± 4320	59,586 ± 3300
OUT				
6 h	424 ± 35^B^	436 ± 4^B^	597 ± 35^A^	388 ± 20^B^
24 h	416 ± 24^b^	478 ± 29^B^	606 ± 14^A^	440 ± 34^Bb^
72 h	434 ± 29^C^	500 ± 19^B^	953 ± 25^A^	459 ± 14^BC^

Note

Peer data shoulder letter with the same case means significant (*p* < .05), letter with different case means extremely significant (*p* < .01).

**FIGURE 5‐1:**
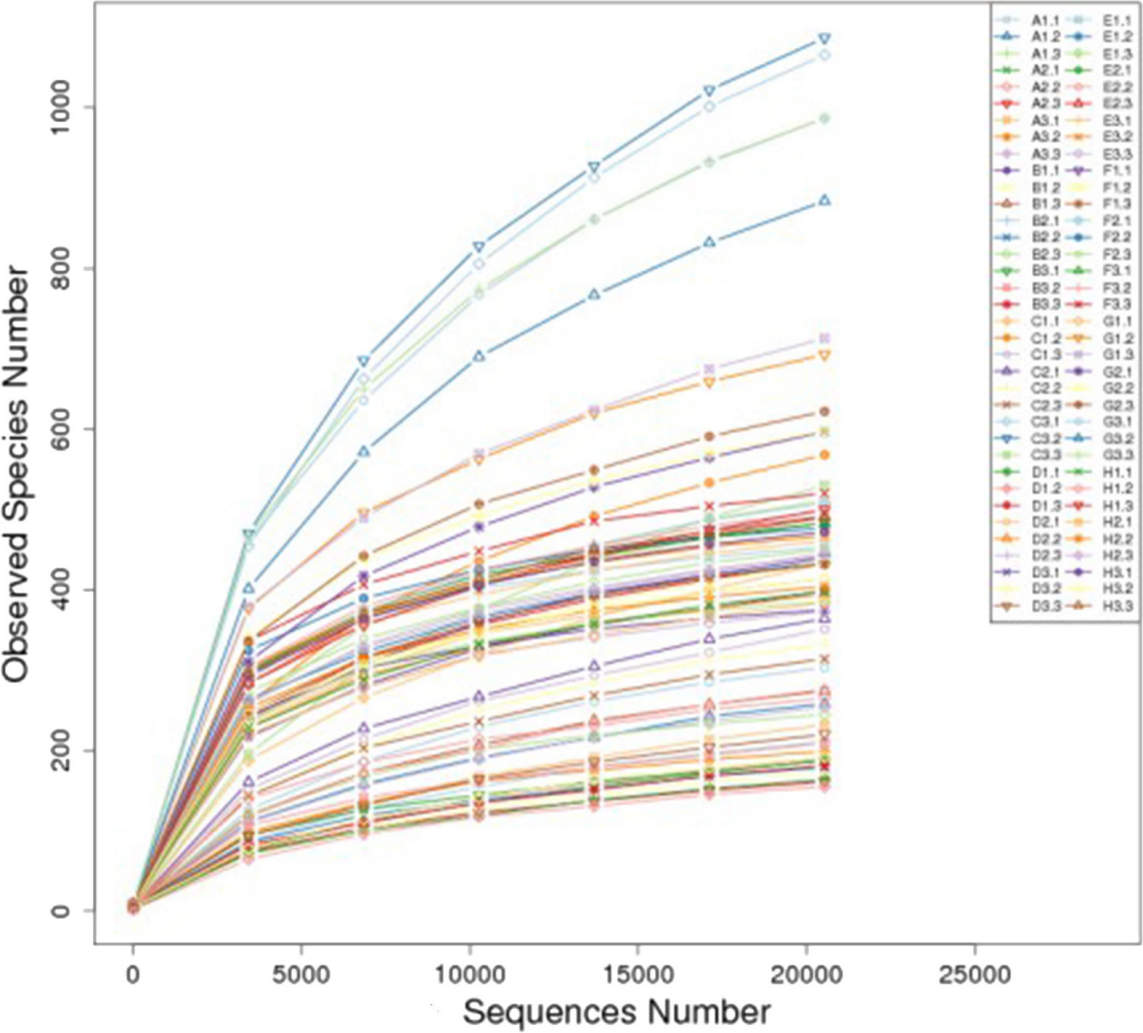
Rarefaction curves of samples

**FIGURE 5‐2:**
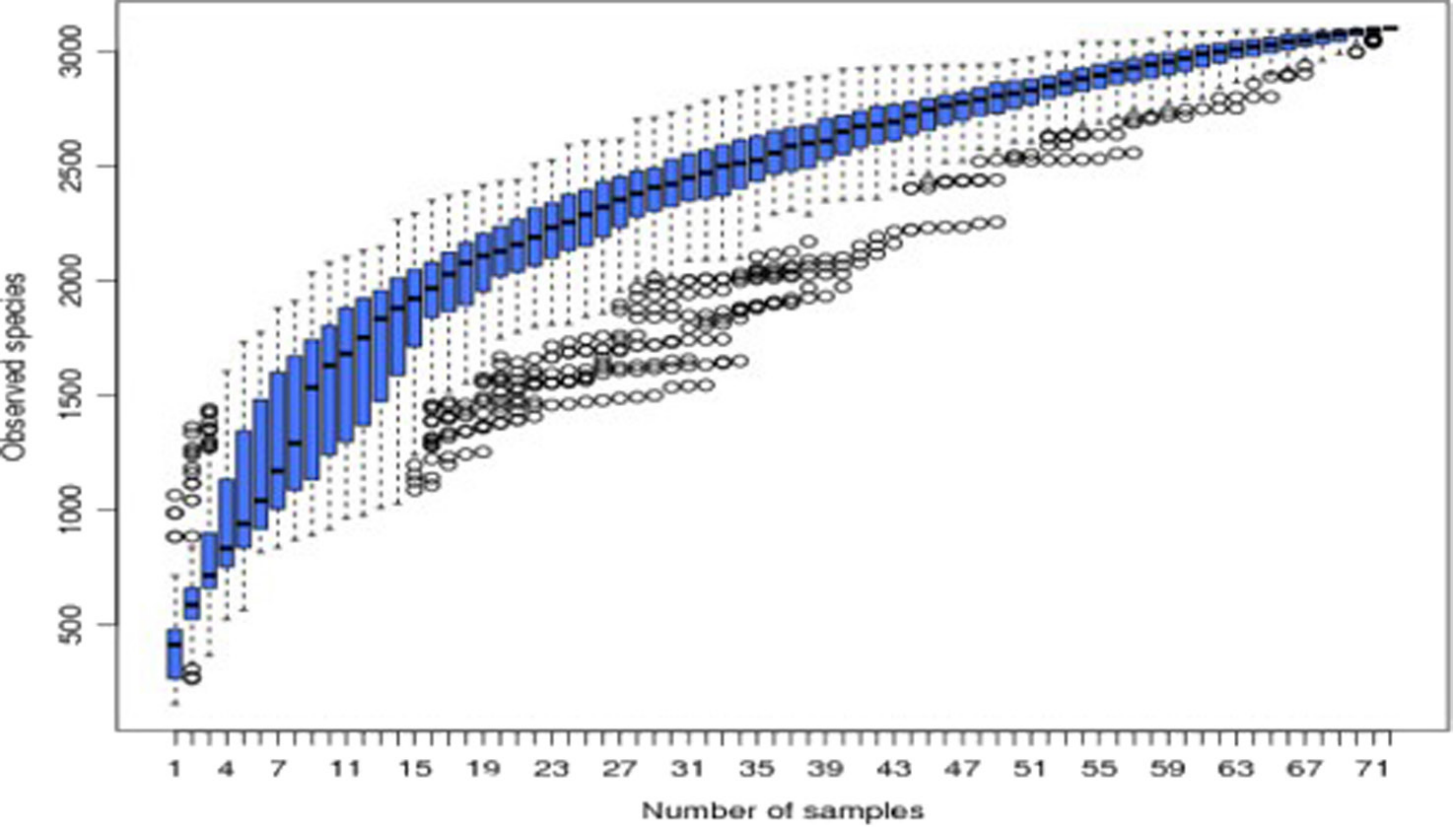
species accumulation boxplot

In summary, rarefaction curves of each sample eventually tend to be flat, indicating that the sequencing data of this test are reasonable and can include most microorganisms in the sample. Among them, 22% CP had the highest gut species richness.

#### 3.6.2 Alpha and beta diversity analysis

Alpha diversity reflects microbial community richness and diversity within a sample. As shown in Tables 3‐3 and 3‐4, there were no significant differences in alpha diversity among the groups (*p* > .05), but the high protein level groups (22% CP and 24% CP) displayed numerically higher alpha diversity than the 20% CP group, and this was more significant at 72 h of feeding. In all groups, the alpha diversity index in the colon at each time point was higher than the corresponding ileal alpha diversity index. This indicates that 22% and 24% levels of CP will increase the abundance of chyme in the intestine.

**TABLE 3‐3:** Microbial alpha diversity index of ileal chyme

Parameter	Groups			
	18% CP	20% CP	22% CP	24% CP
Chao				
6 h	355.67 ± 26.15	332.82 ± 53.34	333.67 ± 17.55	347.36 ± 31.87
24 h	343.76 ± 18.80	352.51 ± 11.85	361.77 ± 29.12	364.84 ± 29.69
72 h	342.94 ± 24.41	348.90 ± 32.57	362.01 ± 48.92	359.23 ± 53.24
ACE				
6 h	327.35 ± 43.77	324.81 ± 36.34	338.74 ± 31.49	335.07 ± 42.62
24 h	314.16 ± 16.69	315.38 ± 41.56	322.08 ± 30.58	329.65 ± 32.70
72 h	333.54 ± 40.59	329.63 ± 33.09	341.88 ± 31.25	333.99 ± 45.89
Simpson				
6 h	0.821 ± 0.067	0.824 ± 0.022	0.812 ± 0.028	0.793 ± 0.034
24 h	0.799 ± 0.039	0.819 ± 0.059	0.797 ± 0.019	0.821 ± 0.074
72 h	0.801 ± 0.045	0.802 ± 0.053	0.817 ± 0.043	0.812 ± 0.055
Shannon				
6 h	3.220 ± 0.210	3.318 ± 0.447	3.168 ± 0.362	3.130 ± 0.144
24 h	2.501 ± 0.277	2.989 ± 0.197	2.646 ± 0.308	3.014 ± 0.345
72 h	3.262 ± 0.356	3.441 ± 0.497	3.630 ± 0.382	3.532 ± 0.307

Note

Peer data shoulder letter with the same case means significant (*p* < .05), letter with different case means extremely significant (*p* < .01).

**TABLE 3‐4:** Microbial alpha diversity index of colonic chyme

Parameter	Groups			
	18% CP	20% CP	22% CP	24% CP
Chao				
6 h	510.64 ± 35.34	526.62 ± 22.63	535.20 ± 22.63	521.78 ± 49.22
24 h	522.13 ± 48.83	532.19 ± 35.30	541.27 ± 41.64	538.02 ± 35.47
72 h	519.13 ± 34.03	525.29 ± 25.34	538.31 ± 30.37	524.64 ± 29.61
ACE				
6 h	499.94 ± 35.87	536.94 ± 30.35	545.49 ± 45.73	495.84 ± 38.48
24 h	507.74 ± 32.15	556.87 ± 56.24	563.03 ± 37.22	502.85 ± 51.56
72 h	521.57 ± 7.56	574.13 ± 6.92	577.53 ± 54.37	534.59 ± 45.21
Simpson				
6 h	0.963 ± 0.021	0.934 ± 0.031	0.944 ± 0.035	0.941 ± 0.027
24 h	0.943 ± 0.037	0.951 ± 0.029	0.950 ± 0.026	0.961 ± 0.008
72 h	0.957 ± 0.023	0.978 ± 0.006	0.953 ± 0.018	0.958 ± 0.017
Shannon				
6 h	6.255 ± 0.490	6.084 ± 0.701	6.116 ± 0.287	6.104 ± 0.225
24 h	6.013 ± 0.132	6.194 ± 0.415	6.182 ± 0.423	6.085 ± 0.267
72 h	6.024 ± 0.406	6.526 ± 0.443	6.468 ± 0.209	6.112 ± 0.413

Note

Peer data shoulder letter with the same case means significant (*p* < .05), letter with different case means extremely significant (*p* < .01).

Beta diversity is a comparative analysis of the microbial community composition of different samples; it mainly includes principal component analysis and principal coordinate analysis. Principal component analysis was able to extract two coordinate axes that maximally reflected the differences between samples; the more similar the community composition of the samples is, the closer they are to each other in the PCA plot. As shown in Figure 5‐3, in the ileal digesta, the difference contribution of principal component I was 26.8%, and the difference contribution of principal component II was 18.4%; most of the samples within each group and the samples taken at each time point were close to each other. Samples C3, D2, D3 were distant from each of the remaining samples. As shown in Figure 5‐4, in the colonic digesta, the difference contribution of principal component I was 14.2%, and the difference contribution of principal component II was 9.4%; except for G1 and G3, the samples within each group were close to each other. The points in the G1 and G3 groups were distant from each other, and they were also distant from the samples obtained from the other groups. It can be seen that both CP and feeding time had large effects on the bacterial community differences in the ileum and colon.

**FIGURE 5‐3:**
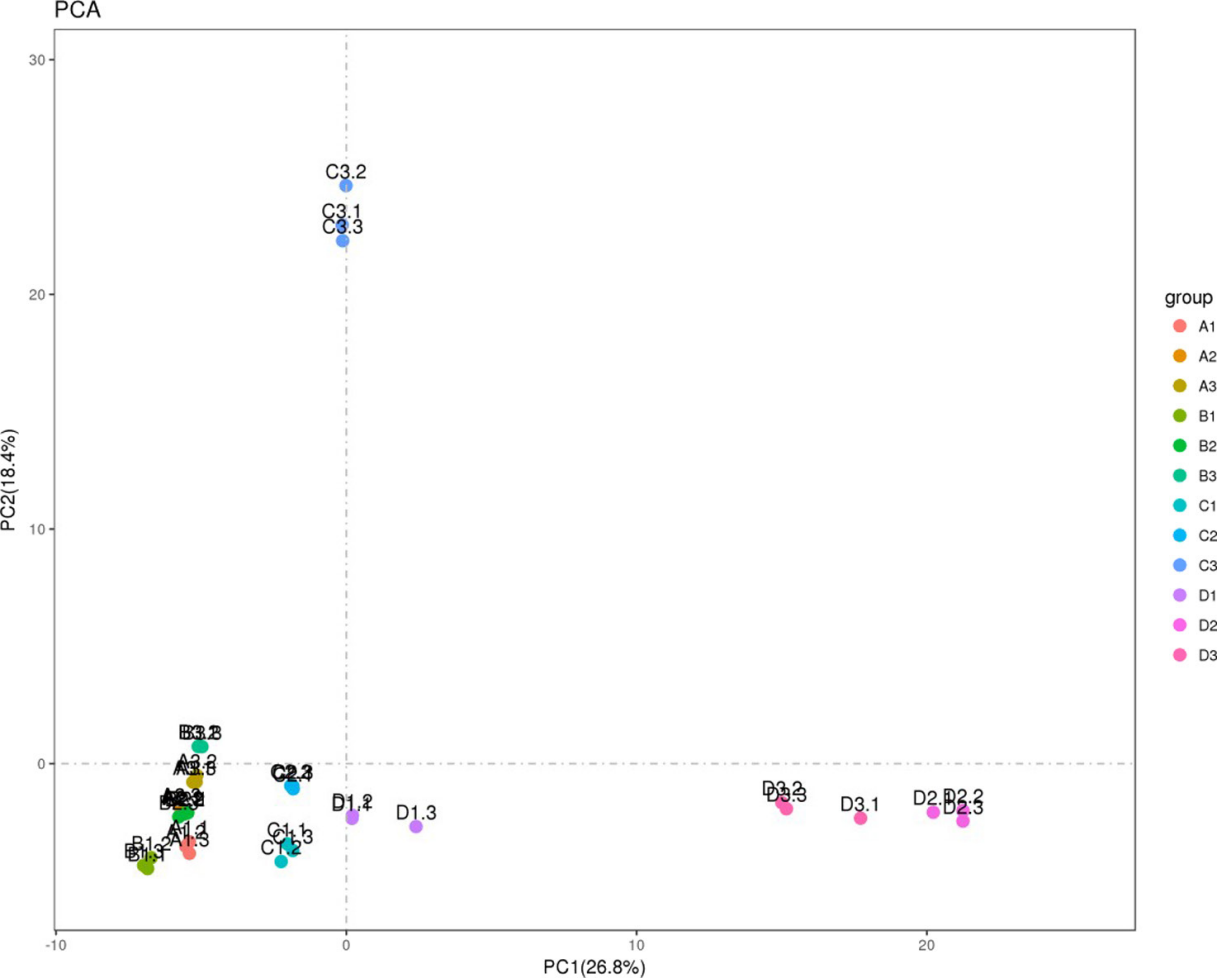
PCA profile of bacteria in ileum

**FIGURE 5‐4:**
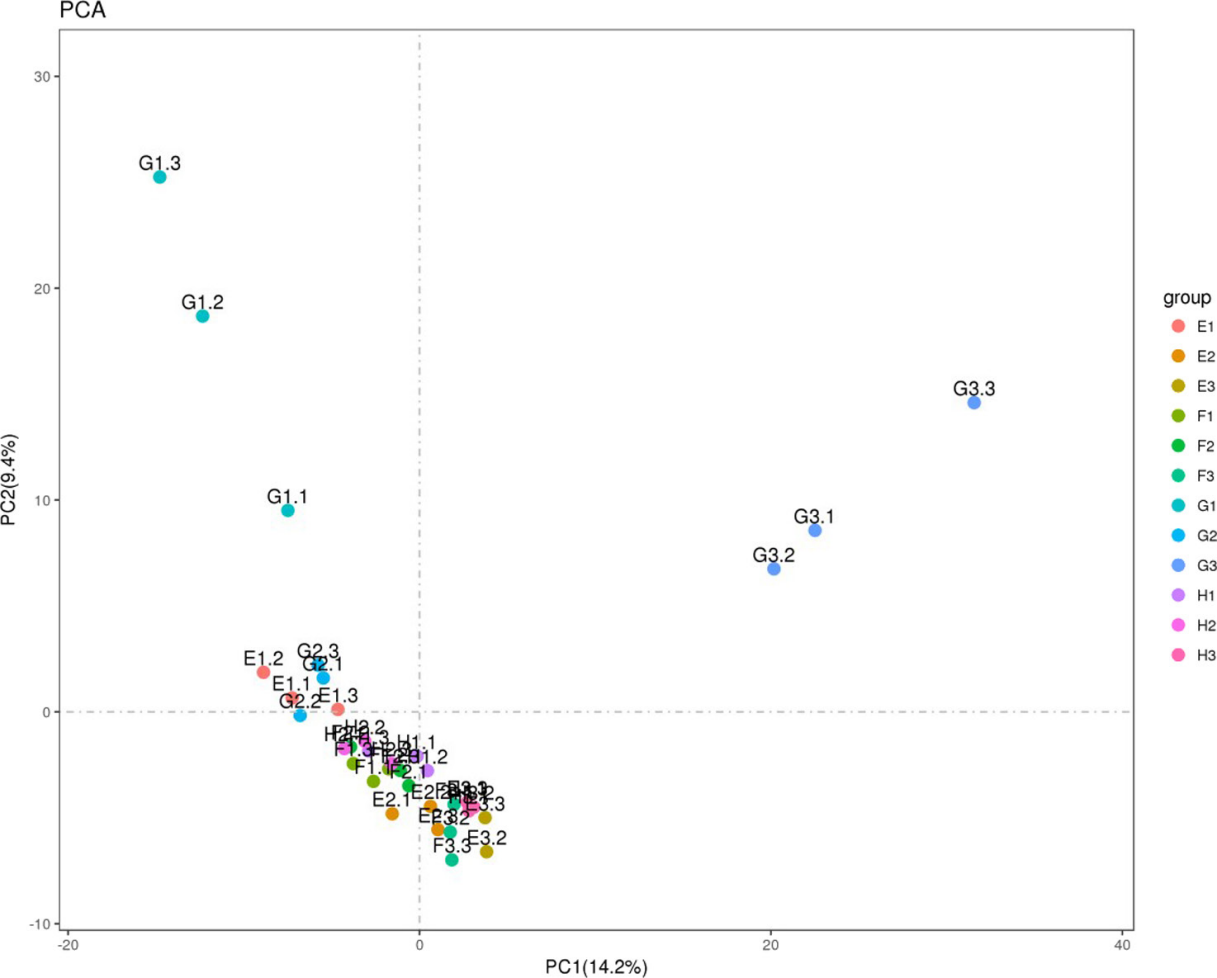
PCA profile of bacteria in colon

#### 3.6.3 Species composition of ileal bacteria

Figure 6‐1 shows the ileal digesta species composition at the phylum level; 18 bacterial phyla and 1 archaeon were obtained for each sample in this assay. Firmicutes, Proteobacteria, and Bacteroidetes were the dominant phyla in all samples except for sample B2 (the relative content of these phyla accounted for 83.22%–97.94% of the total sequences); in sample B2, the relative content of *Aspergillus* was the highest. As shown in Table 4‐1, after 72 h of feeding, the relative content of Firmicutes in the 22% CP and 24% CP groups was very significantly lower than that in the 18% CP and 20% CP groups (*p* < .01). After feeding for 24–72 h, the relative content of Proteobacteria gradually increased as the dietary protein level increased. The relative content of Bacteroidetes in the 24% CP group was significantly higher than that in the other three protein level groups after 24–72 h of feeding (*p* < .01).

**FIGURE 6‐1:**
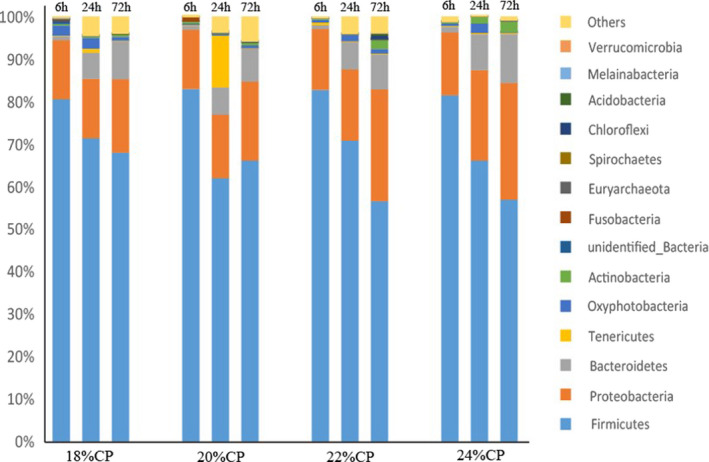
Composition of ileal bacteria at phylum level

**TABLE 4‐1:** Effect of diet on microbial phylum level of ileal chyme

Classification		Groups						
		18% CP		20% CP		22% CP		24% CP
Firmicutes								
6 h	80.50 ± 7.68^A^		82.08 ± 5.89^A^		82.74 ± 2.05^A^		81.44 ± 5.84^A^	
24 h	71.25 ± 6.17^A^		61.89 ± 7.80^A^		70.74 ± 3.32^A^		66.06 ± 3.17^A^	
72 h	67.91 ± 3.52^A^		66.00 ± 3.29^A^		56.55 ± 2.85^B^		56.33 ± 2.85^B^	
Proteobacteria								
6 h	13.91 ± 2.72^A^		13.80 ± 3.29^A^		14.32 ± 3.34^A^		14.81 ± 2.58^A^	
24 h	13.99 ± 4.53^a^		14.94 ± 1.46^a^		16.74 ± 1.80^Aa^		21.31 ± 1.52^A^	
72 h	17.25 ± 3.00^B^		18.59 ± 2.28^B^		26.27 ± 2.72^A^		27.18 ± 3.10^A^	
Bacteroidetes								
6h	0.91 ± 0.16^A^		1.00 ± 0.10^Aa^		0.87 ± 0.01^A^		1.32 ± 0.33^a^	
24h	6.16 ± 1.44^B^		6.39 ± 1.04^B^		6.50 ± 1.06^B^		8.45 ± 1.39^A^	
72h	8.98 ± 0.49^B^		7.76 ± 0.44^B^		8.28 ± 0.83^B^		11.40 ± 0.87^A^	
Actinobacteria								
6 h	0.38 ± 0.09^A^		0.41 ± 0.05^b^		0.20 ± 0.02^C^		0.28 ± 0.02^BC^	
24 h	0.24 ± 0.03^B^		0.21 ± 0.02^B^		0.18 ± 0.01^B^		1.43 ± 0.47^A^	
72 h	0.49 ± 0.03^B^		0.62 ± 0.04^B^		2.10 ± 0.28^a^		2.64 ± 0.29^A^	
Tenericutes								
6 h	0.14 ± 0.02^B^		0.12 ± 0.01^B^		0.64 ± 0.06^A^		0.22 ± 0.03^b^	
24 h	0.89 ± 0.15^B^		12.17 ± 0.94^A^		0.16 ± 0.01^B^		0.24 ± 0.03^B^	
72 h	0.11 ± 0.02^C^		0.10 ± 0.01^BC^		0.15 ± 0.02^Ab^		0.18 ± 0.03^A^	
Oxyphotobacteria								
6 h	2.36 ± 0.34^B^		0.36 ± 0.62^A^		0.63 ± 0.02^C^		0.54 ± 0.02^C^	
24 h	2.54 ± 0.48^A^		0.27 ± 0.04^C^		1.45 ± 0.13^B^		2.28 ± 0.23^A^	
72 h	0.87 ± 0.06^AB^		0.66 ± 0.01^b^		1.07 ± 0.19^A^		0.15 ± 0.02^C^	
Fusobacteria								
6 h	0.30 ± 0.03^b^		1.00 ± 0.21^A^		0.030 ± 0.002^B^		0.020 ± 0.001^B^	
24 h	0.036 ± 0.006^B^		0.058 ± 0.003^A^		0.0065 ± 0.0004^C^		0.0065 ± 0.0003^C^	
72 h	0.033 ± 0.005^A^		0.029 ± 0.001^A^		0.0097 ± 0.0002^B^		0.0032 ± 0.0004^b^	

Note

Peer data shoulder letter with the same case means significant (*p* < .05), letter with different case means extremely significant (*p* < .01).

Figure 6‐2 shows the 20 genera with high relative abundance in the test samples. As shown in Table 4‐2, *Lactobacillus* (13.84%−64.84%) was the dominant genus in all samples, and its relative content showed a decreasing trend as the dietary protein level increased. After feeding for 72 h, the relative content of *Lactobacillus* in the 24% CP group was significantly lower than that in the 18% CP group (*p* < .05). The relative content of unidentified Clostridiales is second only to *Lactobacillus* spp. Except for the 18% CP group, the relative content of *Actinobacillus* showed a decreasing trend with increasing feeding time, and the relative content of *Actinobacillus* in the 20% CP group was significantly lower than that in the 22% CP and 24% CP groups (*p* < .05); however, its relative content in the 18% CP group was very significantly higher than that in the other three groups at 72 h of feeding (*p* < .01).

**FIGURE 6‐2:**
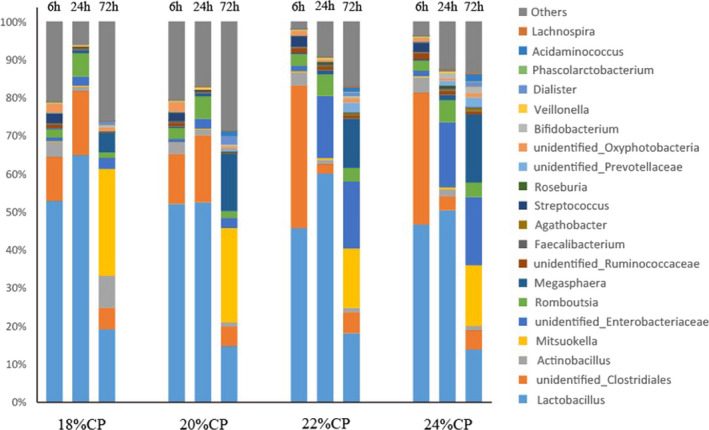
Composition of ileal bacteria at genus level

**TABLE 4‐2:** Effect of diet on microbial genus level of ileal chyme

Classification	Groups			
	18% CP	20% CP	22% CP	24% CP
*Lactobacillus*				
6 h	52.83 ± 2.70^A^	52.07 ± 3.93^A^	45.73 ± 6.46^A^	46.65 ± 4.48^A^
24 h	64.84 ± 8.57^A^	52.49 ± 6.60^A^	60.10 ± 8.92^A^	50.40 ± 6.80^A^
72 h	19.15 ± 3.48^A^	14.71 ± 1.82^Aa^	18.13 ± 2.48^Aa^	13.84 ± 2.28^a^
*Actinobacillus*				
6 h	4.08 ± 0.31^A^	2.95 ± 0.30^A^	3.34 ± 0.46^A^	4.04 ± 0.98^A^
24 h	1.15 ± 0.27^A^	1.49 ± 0.32^Aa^	1.11 ± 0.24^A^	1.69 ± 0.16^a^
72 h	8.38 ± 2.15^B^	1.07 ± 0.40^A^	1.09 ± 0.35^A^	1.17 ± 0.32^A^
*unidentified_Clostridiales*				
6 h	11.55 ± 1.77^A^	13.16 ± 2.59^A^	37.51 ± 5.64^B^	34.62 ± 4.59^B^
24 h	16.92 ± 1.04^A^	17.64 ± 0.93^A^	2.45 ± 0.52^B^	3.74 ± 0.99^B^
72 h	5.65 ± 0.32^A^	5.17 ± 0.63^A^	5.56 ± 0.48^A^	5.01 ± 0.79^A^
*Megasphaera*				
6 h	038 ± 0.06^A^	0.33 ± 0.06^A^	0.31 ± 0.07^A^	0.29 ± 0.05^A^
24 h	1.49 ± 0.40^A^	1.08 ± 0.25^Aa^	0.83 ± 0.20^a^	0.80 ± 0.21^a^
72 h	5.05 ± 1.34^B^	15.08 ± 2.78^Aa^	12.95 ± 1.83^a^	17.86 ± 2.49^A^
*unidentified_Ruminococcaceae*				
6 h	0.81 ± 0.10^a^	0.95 ± 0.11^Aa^	1.21 ± 0.34^Aa^	1.44 ± 0.38^A^
24 h	0.15 ± 0.04^A^	0.17 ± 0.04^A^	0.82 ± 0.16^B^	0.87 ± 0.25^B^
72 h	0.09 ± 0.02^A^	0.07 ± 0.02^A^	0.66 ± 0.10^B^	0.72 ± 0.05^B^
*Bifidobacterium*				
6 h	0.033 ± 0.004^Aa^	0.036 ± 0.003^A^	0.027 ± 0.006^a^	0.032 ± 0.002^Aa^
24 h	0.035 ± 0.005^A^	0.036 ± 0.008^A^	0.028 ± 0.003^A^	0.027 ± 0.004^A^
72 h	0.54 ± 0.05^A^	0.52 ± 0.04^Aa^	0.42 ± 0.06^a^	0.43 ± 0.05^a^
*Roseburia*				
6 h	0.15 ± 0.03^A^	0.14 ± 0.04^A^	0.18 ± 0.05^A^	0.16 ± 0.04^A^
24 h	0.07 ± 0.02^A^	0.06 ± 0.01^A^	0.46 ± 0.05^B^	0.48 ± 0.05^B^
72 h	0.023 ± 0.003^A^	0.054 ± 0.005^B^	0.081 ± 0.012^C^	0.085 ± 0.013^C^
*Mitsuokella*				
6 h	0.10 ± 0.04^A^	0.11 ± 0.02^A^	0.37 ± 0.06^B^	0.31 ± 0.04^B^
24 h	0.27 ± 0.06^A^	0.23 ± 0.04^A^	0.46 ± 0.07^B^	0.59 ± 0.06^b^
72 h	28.12 ± 4.71^a^	24.77 ± 5.45^Aa^	15.60 ± 3.15^A^	15.93 ± 5.14^A^
*Streptococcus*				
6 h	2.55 ± 0.34^A^	2.13 ± 0.24^A^	2.65 ± 0.26^A^	2.31 ± 0.33^A^
24 h	0.22 ± 0.05^a^	0.26 ± 0.06^a^	0.29 ± 0.05^Aa^	0.39 ± 0.08^A^
72 h	0.050 ± 0.011^a^	0.073 ± 0.012^Aa^	0.072 ± 0.014^Aa^	0.077 ± 0.009^A^
*Veillonella*				
6 h	0.21 ± 0.04^A^	0.15 ± 0.03^A^	0.20 ± 0.04^A^	0.19 ± 0.02^A^
24 h	0.34 ± 0.01^a^	0.36 ± 0.05^Aa^	0.36 ± 0.03^Aa^	0.42 ± 0.03^A^
72 h	0.24 ± 0.02^A^	0.06 ± 0.01^C^	0.08 ± 0.01^C^	0.19 ± 0.02^B^

Note

Peer data shoulder letter with the same case means significant (*p* < .05), letter with different case means extremely significant (*p* < .01).

#### 3.6.4 Species composition of colonic bacteria

The species composition of colonic digesta at the phylum level is shown in Figure 6‐3; 20 bacterial phyla and 1 archaeal phylum were obtained from each sample in this experiment. Firmicutes, Proteobacteria, and Bacteroidetes were the dominant phyla in all samples except sample H1 (the relative content of these phyla accounted for 88.44%–97.26% of the total sequences). As shown in Table 4‐3, the content of Firmicutes first increased and then decreased as the feeding time increased. However, the relative contents of both Proteobacteria and Bacteroidetes first decreased and then increased with feeding time. At 24 h of feeding, the relative content of Bacteroidetes was significantly lower in the 22% CP group than in the 20% CP and 24% CP groups (*p* < .05). After 6 h of feeding, the relative content of Proteobacteria increased as the protein level in the diet increased, and the relative content of Proteobacteria in the 22% CP and 24% CP groups was very significantly lower than that in the 18% CP and 20% CP groups (*p* < .01). The relative content of Actinobacteria gradually decreased with increasing feeding time; at 6 h of feeding, the relative Actinobacteria content of the 24% CP group was very significantly higher than that of the other three groups, but after 72 h of feeding, it was very significantly lower than that of the 22% CP group (*p* < .01) and significantly lower than that of the 20% CP group (*p* < .05).

**FIGURE 6‐3:**
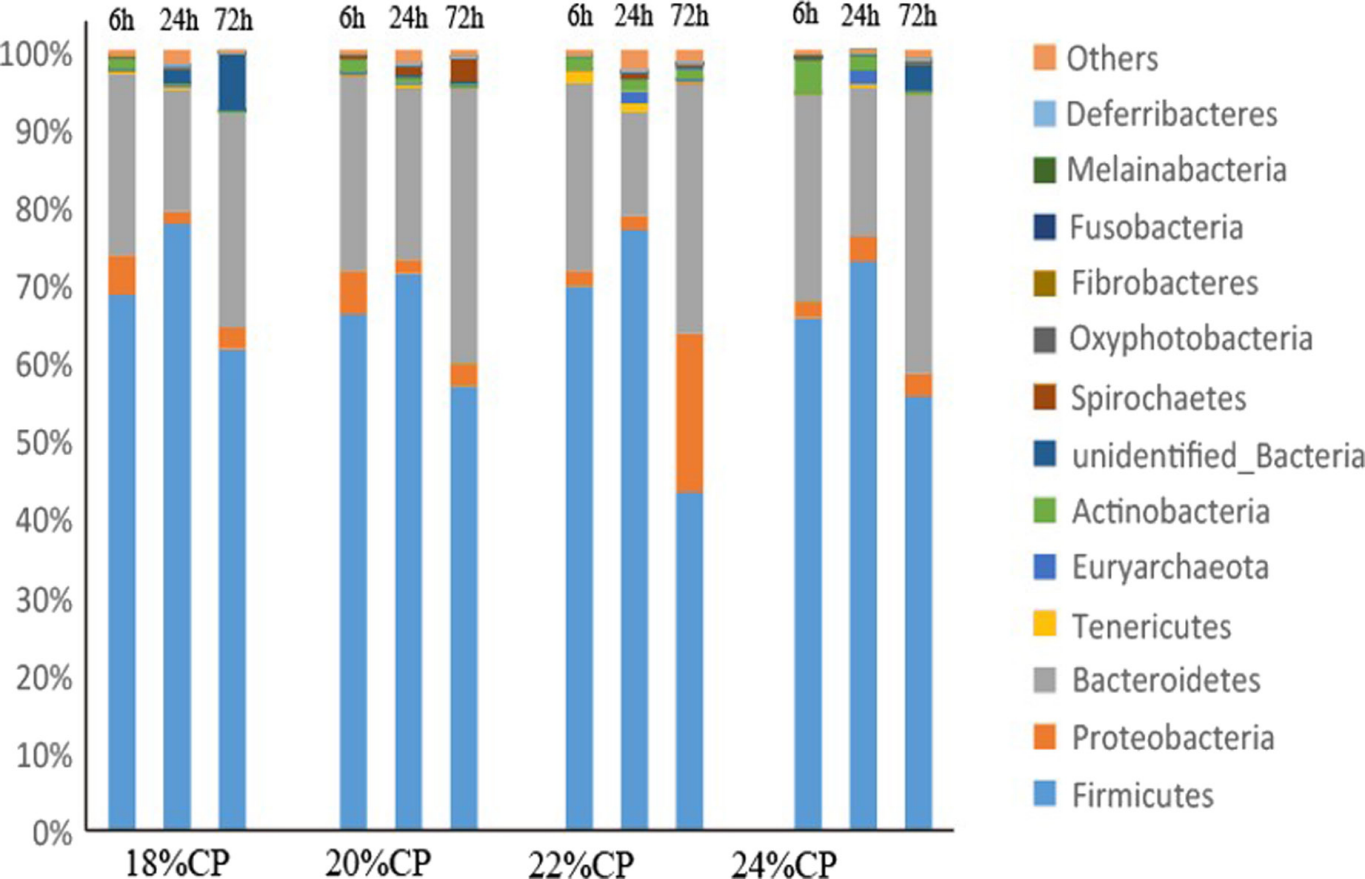
Composition of colonic bacteria at phylum level

**TABLE 4‐3:** Effect of diet on microbial phylum level of colonic chyme

Classification	Groups			
	18% CP	20% CP	22% CP	24% CP
*Firmicutes*				
6 h	68.89 ± 4.60^A^	66.48 ± 4.61^A^	69.99 ± 2.74^A^	65.94 ± 2.66^A^
24 h	78.05 ± 5.04^A^	68.10 ± 2.71^a^	77.30 ± 4.88^A^	73.22 ± 5.50^Aa^
72 h	61.96 ± 3.66^A^	57.16 ± 6.47^a^	43.40 ± 4.00^B^	54.36 ± 2.62^Aa^
*Proteobacteria*				
6 h	5.08 ± 0.32^A^	5.51 ± 0.48^A^	2.06 ± 0.16^B^	2.17 ± 0.33^B^
24 h	1.51 ± 0.27^B^	1.70 ± 0.26^B^	1.77 ± 0.26^B^	3.22 ± 0.46^A^
72 h	2.77 ± 0.09^B^	2.96 ± 0.43^B^	20.26 ± 4.12^A^	2.87 ± 0.19^B^
*Bacteroidetes*				
6 h	23.29 ± 0.84^A^	25.00 ± 0.37^A^	24.13 ± 2.57^A^	26.41 ± 1.88^A^
24 h	15.72 ± 3.04^Aa^	20.96 ± 3.62^A^	13.42 ± 1.38^a^	19.14 ± 2.40^A^
72 h	27.44 ± 1.67^a^	35.31 ± 4.30^Aa^	32.00 ± 3.45^Aa^	35.43 ± 1.78^A^
*Actinobacteria*				
6 h	1.47 ± 0.05^B^	1.70 ± 0.27^B^	1.64 ± 0.06^B^	4.51 ± 0.75^A^
24 h	0.23 ± 0.02^B^	0.68 ± 0.08^B^	1.58 ± 0.18^A^	1.90 ± 0.46^A^
72 h	0.21 ± 0.05^b^	0.33 ± 0.01^B^	1.31 ± 0.13^A^	0.26 ± 0.02^b^
*Tenericutes*				
6 h	0.27 ± 0.03^B^	0.23 ± 0.03^B^	1.60 ± 0.31^A^	0.117 ± 0.002^B^
24 h	0.28 ± 0.05^b^	0.41 ± 0.04^B^	1.03 ± 0.06^A^	0.38 ± 0.05^B^
72 h	0.07 ± 0.01^B^	0.20 ± 0.04^A^	0.29 ± 0.04^a^	0.21 ± 0.03^A^
*Euryarchaeota*				
6 h	0.30 ± 0.02^A^	0.26 ± 0.05^A^	0.018 ± 0.003^B^	0.010 ± 0.001^B^
24 h	0.20 ± 0.04^B^	0.18 ± 0.01^B^	1.49 ± 0.36^A^	1.83 ± 0.25^A^
72 h	0.05 ± 0.01^B^	0.07 ± 0.01^B^	0.24 ± 0.04^A^	0.23 ± 0.02^A^
*Spirochaetes*				
6 h	0.21 ± 0.03^B^	0.44 ± 0.03^A^	0.023 ± 0.002^C^	0.08 ± 0.01^c^
24 h	0.19 ± 0.02^C^	1.08 ± 0.16^A^	0.69 ± 0.08^B^	0.12 ± 0.01^C^
72 h	0.10 ± 0.01^B^	2.76 ± 0.40^A^	0.27 ± 0.05^B^	0.36 ± 0.03^B^

Note

Peer data shoulder letter with the same case means significant (*p* < .05), letter with different case means extremely significant (*p* < .01).

Figure 6‐4 and Table 4‐4 show the 22 genera that were present in high relative abundance; of these, *Lactobacillus* (4.03%–20.91%) was the most dominant genus among all samples, and its relative content was negatively correlated with feeding time. After 72 h of feeding, the relative *Lactobacillus* content of the 20% CP group was significantly higher than that of the 22% CP group (*p* < .05). The relative content of unidentified Ruminococcaceae was second only to *Lactobacillus* spp., and the relative content of unidentified Ruminococcaceae in the 22% CP group was very significantly higher than that of the other three groups at 24 h of feeding (*p* < .01); however, after 72 h of feeding, the relative content of unidentified Ruminococcaceae in the 22% CP and 24% CP groups was very significantly lower than that of the 18% CP and 20% CP groups (*p* < .01). The relative content of *Faecalibacterium* species was not significantly different between the groups at 24 h and 72 h of feeding (*p* > .05), and all groups showed a trend of first increasing and then decreasing with increased feeding time. The relative content of *Roseburia* showed a decreasing trend with increasing feeding time; at 24 h of feeding, it was significantly higher in the 20% CP group than in the 18% CP and 22% CP groups, and at 72 h of feeding it was significantly lower in the 22% CP and 24% CP groups than in the 20% CP group (*p* <.05).

**FIGURE 6‐4:**
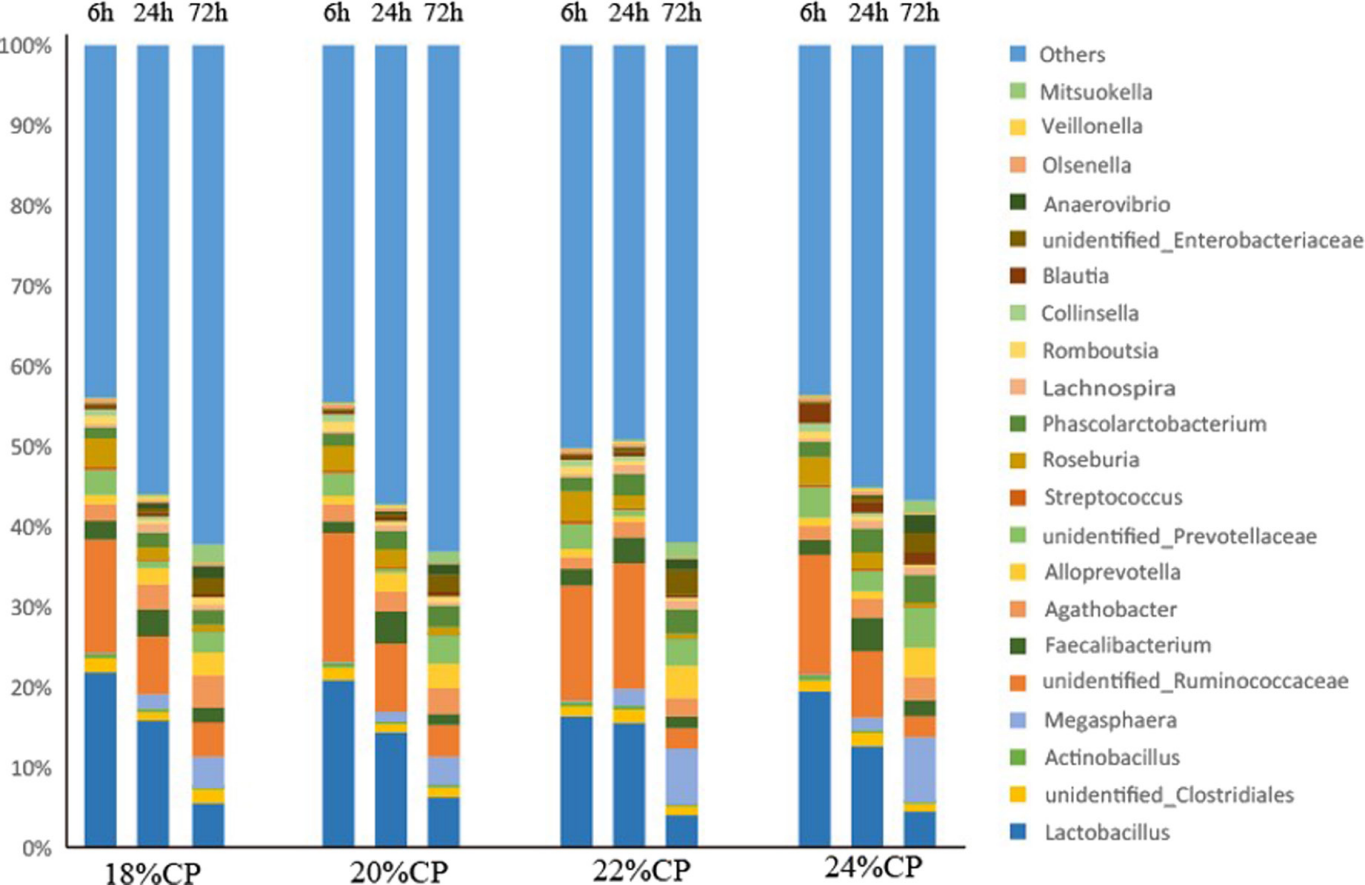
Composition of colonic bacteria at genus level

**TABLE 4‐4:** Effect of diet on microbial genus level of colonic chyme

Classification	Groups			
	18% CP	20% CP	22% CP	24% CP
*Lactobacillus*				
6 h	21.85 ± 4.24^A^	20.91 ± 3.79^A^	16.36 ± 2.22^A^	19.49 ± 2.35^A^
24 h	15.85 ± 2.44^A^	14.39 ± 3.57^A^	15.57 ± 3.56^A^	12.66 ± 2.05^A^
72 h	5.54 ± 0.66^Aa^	6.27 ± 0.74^A^	4.03 ± 1.28^a^	4.48 ± 1.04^Aa^
*Actinobacillus*				
6 h	0.59 ± 0.04^A^	0.63 ± 0.04^A^	0.59 ± 0.09^A^	0.64 ± 0.13^A^
24 h	0.45 ± 0.07^Aa^	0.21 ± 0.03^B^	0.48 ± 0.04^A^	0.36 ± 0.05^a^
72 h	0.18 ± 0.03^B^	0.33 ± 0.04^A^	0.19 ± 0.02^B^	0.15 ± 0.02^B^
*unidentified_Clostridiales*				
6 h	1.77 ± 0.15^A^	1.53 ± 0.17^Aa^	1.22 ± 0.33^a^	1.34 ± 0.33^Aa^
24 h	1.02 ± 0.17^a^	1.08 ± 0.05^a^	1.63 ± 0.41^A^	1.63 ± 0.26^A^
72 h	1.73 ± 0.07^A^	1.21 ± 0.12^B^	1.10 ± 0.13^B^	1.01 ± 0.26^B^
*Megasphaera*				
6 h	0.09 ± 0.02^A^	0.10 ± 0.02^A^	0.13 ± 0.03^A^	0.12 ± 0.02^A^
24 h	1.79 ± 0.17^Aa^	1.28 ± 0.25^a^	2.07 ± 0.34^A^	1.54 ± 0.31^Aa^
72 h	3.89 ± 0.62^B^	3.51 ± 0.39^B^	7.02 ± 0.94^A^	8.12 ± 1.17^A^
*unidentified_Ruminococcaceae*				
6 h	14.12 ± 2.54^A^	16.08 ± 1.83^A^	14.40 ± 1.61^A^	14.91 ± 2.07^A^
24 h	7.23 ± 0.78^B^	8.53 ± 0.82^B^	15.67 ± 1.69^A^	8.28 ± 0.75^B^
72 h	4.25 ± 0.69^A^	3.92 ± 0.90^A^	2.56 ± 0.47^B^	2.62 ± 0.37^B^
*Faecalibacterium*				
6 h	2.33 ± 0.36^A^	1.45 ± 0.29^a^	2.01 ± 0.42^Aa^	1.86 ± 0.28^Aa^
24 h	3.33 ± 0.56^A^	3.99 ± 0.44^A^	3.23 ± 0.52^A^	4.18 ± 0.43^A^
72 h	1.85 ± 0.45^A^	1.43 ± 0.15^A^	1.43 ± 0.27^A^	2.02 ± 0.30^A^
*Roseburia*				
6 h	3.02 ± 0.75^A^	3.37 ± 0.25^A^	3.46 ± 0.47^A^	3.43 ± 0.54^A^
24 h	1.55 ± 0.29^a^	2.23 ± 0.31^A^	1.48 ± 0.43^a^	2.03 ± 0.18^Aa^
72 h	0.96 ± 0.17^A^	0.79 ± 0.23^Aa^	0.54 ± 0.17^a^	0.49 ± 0.02^a^
*Mitsuokella*				
6 h	0.15 ± 0.02^A^	0.17 ± 0.03^A^	0.18 ± 0.02^A^	0.20 ± 0.04^A^
24 h	0.28 ± 0.03^A^	0.19 ± 0.04^a^	0.25 ± 0.05^Aa^	0.22 ± 0.03^Aa^
72 h	2.31 ± 0.19^A^	1.41 ± 0.29^B^	1.86 ± 0.20^AB^	1.58 ± 0.27^B^
*Streptococcus*				
6 h	0.34 ± 0.03^A^	0.37 ± 0.05^A^	0.38 ± 0.02^A^	0.32 ± 0.06^A^
24 h	0.19 ± 0.08^a^	0.26 ± 0.05^Aa^	0.32 ± 0.04^A^	0.28 ± 0.04^Aa^
72 h	0.047 ± 0.003^aB^	0.044 ± 0.009^B^	0.063 ± 0.004^A^	0.062 ± 0.005^A^
*Anaerovibrio*				
6 h	0.055 ± 0.005^A^	0.062 ± 0.008^A^	0.053 ± 0.005^A^	0.060 ± 0.006^A^
24 h	0.68 ± 0.10^A^	0.28 ± 0.06^B^	0.18 ± 0.02^B^	0.26 ± 0.06^B^
72 h	1.53 ± 0.15^B^	1.29 ± 0.39^B^	1.39 ± 0.14^B^	2.32 ± 0.24^A^

Note

Peer data shoulder letter with the same case means significant (*p* < .05), letter with different case means extremely significant (*p* < .01).

### 3.7 Copy number results for *Lactobacillus*, *Bifidobacterium*, *Clostridium difficile*, *E*. *coli*, and Roseburia

In Tables 5‐1 and 5‐2, after 6 h of feeding, the number of *E. coli* in the ileum was significantly lower in the 18% CP group than in the 22% CP and 24% CP groups (*p* < .05). After 24 h of feeding, the number of lactobacilli in the colon was significantly lower in the 22% CP and 24% CP groups than in the 20% CP group (*p* < .05), and the numbers of *Bifidobacterium* and *Roseburia* in the ileum of the 24% CP group were very significantly higher than those in the 18% CP and 20% CP groups (*p* < .01). After 72 h of feeding, the number of *C*. *difficile* in the colon of the 22% CP and 24% CP groups was very significantly higher than that in the 18% CP and 20% CP groups (*p* < .01). Above all, the animals fed the high‐protein diets showed an increased change in the numbers of bacteria in the ileal contents and that the number of lactobacilli, bifidobacteria, and Roseburia in the small intestine showed a decreasing trend with increased feeding time, whereas the number of *C*. *difficile* and *E. coli* showed an increasing trend.

**TABLE 5‐1:** Copies of five bacteria in 200 mg ileal chyme

Species	Groups			
	18% CP	20% CP	22% CP	24% CP
*Lactobacillus* (×10^9^)				
6 h	7.56 ± 0.25	7.49 ± 0.28	7.39 ± 0.21	7.44 ± 0.37
24 h	5.64 ± 0.56	5.61 ± 0.33	5.46 ± 0.35	5.41 ± 0.54
72 h	3.61 ± 0.38^Aa^	3.68 ± 0.32^A^	3.03 ± 0.20^a^	3.17 ± 0.29^Aa^
*Bifidobacterium* (×10^5^)				
6 h	2.68 ± 0.36	2.67 ± 0.26	2.65 ± 0.47	2.60 ± 0.25
24 h	1.84 ± 0.25^B^	1.80 ± 0.16^B^	1.75 ± 0.12^B^	1.44 ± 0.16^A^
72 h	0.96 ± 0.13^B^	0.90 ± 0.12^B^	0.82 ± 0.08^AB^	0.63 ± 0.15^A^
*Roseburia* (×10^6^)				
6 h	28.62 ± 6.86	28.80 ± 4.71	31.25 ± 8.42	32.76 ± 4.30
24 h	4.8 ± 0.98^B^	4.73 ± 0.97^B^	60.86 ± 13.15^A^	61.24 ± 14.73^A^
72 h	3.78 ± 0.45^a^	4.06 ± 0.66^Aa^	5.29 ± 0.55^A^	5.16 ± 0.91^A^
*Clostridium difficile* (×10^3^)				
6 h	5.17 ± 0.81	5.31 ± 0.78	5.02 ± 1.07	5.30 ± 0.74
24 h	5.62 ± 0.49^AB^	4.98 ± 0.32^B^	5.78 ± 0.09^A^	5.21 ± 0.16^B^
72 h	5.66 ± 0.30^B^	5.45 ± 0.30^B^	6.59 ± 0.25^A^	6.84 ± 0.38^A^
*Escherichia coli* (×10^4^)				
6 h	1.12 ± 0.22^a^	1.27 ± 0.11^Aa^	1.57 ± 0.11^A^	1.50 ± 0.20^A^
24 h	1.43 ± 0.16^B^	1.85 ± 0.16^A^	1.89 ± 0.06^A^	2.05 ± 0.12^A^
72 h	2.59 ± 0.18^C^	2.65 ± 0.35^C^	7.69 ± 1.10^B^	10.60 ± 1.06^A^

Note

Peer data shoulder letter with the same case means significant (*p* < .05), letter with different case means extremely significant (*p* < .01).

**TABLE 5‐2:** Copies of five bacteria in 200 mg colonic chyme

Species		Groups						
		18% CP		20% CP		22% CP		24% CP
*Lactobacillus* (×10^8^)								
6 h	7.50 ± 0.77		7.17 ± 0.62		6.39 ± 0.48		6.56 ± 0.30	
24 h	5.06 ± 0.37^Aa^		5.04 ± 0.20^A^		4.32 ± 0.37^a^		4.05 ± 0.39^a^	
72 h	1.87 ± 0.23^Aa^		2.01 ± 0.33^A^		1.37 ± 0.30^a^		1.42 ± 0.29^a^	
*Bifidobacterium* (×10^4^)								
6 h	3.82 ± 0.60		3.71 ± 0.89		3.64 ± 0.82		3.47 ± 0.79	
24 h	3.69 ± 0.91		3.58 ± 0.63		3.50 ± 0.66		3.51 ± 0.83	
72 h	1.04 ± 0.27^A^		0.89 ± 0.11^A^		0.61 ± 0.13^B^		0.54 ± 0.10^B^	
*Roseburia* (×10^6^)								
6 h	23.21 ± 4.23		23.32 ± 3.97		24.36 ± 5.07		24.72 ± 4.18	
24 h	12.38 ± 2.82		12.58 ± 2.12		12.82 ± 1.98		13.44 ± 2.51	
72 h	8.17 ± 0.52^A^		7.88 ± 0.43^A^		6.88 ± 0.41^a^		7.00 ± 0.42^a^	
*Clostridium difficile* (×10^3^)								
6 h	8.02 ± 0.38		8.23 ± 0.24		8.14 ± 0.16		8.37 ± 0.54	
24 h	8.69 ± 0.58		8.37 ± 0.43		8.83 ± 0.37		8.71 ± 0.32	
72 h	8.78 ± 0.48^B^		8.55 ± 0.32^B^		9.78 ± 0.22^A^		10.12 ± 0.65^A^	
6 h	2.57 ± 0.36		2.66 ± 0.37		3.01 ± 0.62		3.21 ± 0.24	
*Escherichia coli* (×10^4^)								
24 h	3.40 ± 0.52^B^		3.68 ± 0.27^AB^		4.51 ± 0.51^Ab^		4.81 ± 0.38^A^	
72 h	140.94 ± 12.51^B^		184.44 ± 10.14^B^		285.30 ± 20.57^A^		288.60 ± 22.77^A^	

Note

Peer data shoulder letter with the same case means significant (*p* < .05), letter with different case means extremely significant (*p* < .01).

## 4 DISCUSSION

For the better development of animal husbandry production in China, it is very important to find CP levels that can solve the problems of postweaning growth retardation and diarrhea in piglets used in pig production. Because the intestine is a place in which the absorption and utilization of nutrients occur, intestinal villus damage and crypt hyperplasia will affect the digestion and utilization of dietary protein (Eid et al., 2003). Instead, the morphology of the small intestinal mucosa will also change when the level of protein in the diet is altered. The results of the current study showed that feeding piglets with 20% CP diet reduced intestinal damage, such as clearly crypt and increased intestinal villi, which in turn was conducive to the absorption and utilization of nutrients by piglets. Tight junctions exist between adjacent cells and anastomose to form a continuous fishnet structure that acts to maintain intercellular polarity and prevent penetration, and occludin protein plays an important role in this process (Roxas et al., 2010; Sappington et al., 2003). In this work, it was found that the tight junction structure was blurred, the organelles were damaged, and the intercellular space was enlarged in the 22%–24% CP groups, while the ultrastructure of the ileal tissue was intact in the 20% protein group, except at 24 h. Experiments performed over the same period also revealed that the diarrhea score was significantly higher in the high‐protein groups than in the 20% protein group (*p* < .05), consistent with the experimental results of Htoo (Htoo et al., 2007) and Opapeju (Opapeju et al., 2009). Therefore, it can be speculated that high‐protein diets disrupt the tight junctions of the small intestine, resulting in dysfunction of the small intestinal mucosal barrier and making enterotoxins and their metabolites more likely to invade the internal environment of the small intestine, causing diarrhea. Lectin is a glycoprotein; Zhao (Zhao et al., 2011) added various concentrations of lectin to piglets’ diets and found by immunohistochemistry that occludin protein expression decreased in weaned piglets as the lectin concentration in the diet increased. In the current study, occludin protein was distributed at the tops of epithelial cell junctions in the small intestine and displayed a brown signal in the 20% CP group, while less occludin‐positive expression area and faded staining were observed in the 24% CP group. This may have been caused by severe small intestinal mucosal injury leading to necrosis and shedding of a large number of epithelial cells in animals fed at the 24% CP level. Zhang (B. Zhang & Guo, 2009) found that upregulating the expression of the intestinal tight junction protein occludin and mRNA by changing CP levels reduced intestinal permeability and enhanced intestinal mucosal barrier function in weaned piglets. This is consistent with the results of our experiments. In terms of protein expression, the expression of occludin was significantly higher in the 20% CP group than in the 22% and 24% CP groups, consistent with the results of Wu (Wu et al., 2015) and Gophna (Gophna et al., 2017). The reason for this may be that high levels of CP increase the expression of proinflammatory factors; upregulation of proinflammatory factors leads to decreased expression of occludin in the jejunum and ileum of piglets (Al‐Sadi et al., 2009; Gao et al., 2020).

16S rDNA amplicon sequencing technology is widely used in the comparative analysis of differences in microbial community structure in the natural environment and in human and animal tissues (Matsuki et al., 2004; Yang et al., 2015). It can be seen from Figure 2‐1 that the dilution curve of each sample in the test finally tends to be flat, indicating that the sequencing depth used in this study covers most of the microorganisms that are present in the intestinal tract and that comprehensive microbial bacteria information can be obtained. Alpha diversity reflects microbial community richness and diversity within a sample; commonly used analytical indices of alpha diversity include the Chao index and the ACE index, both of which focus on reflecting community richness. Various dietary additives have been found by Mercè Roca (Roca et al., 2014) to affect the diversity of gut microbes in animals. In the current study, it was found that the Chao index of the ileum and colon of piglets increased with the increase in CP level at 24–72 h after feeding and that the alpha diversity index of the colon was significantly greater than that of the ileum, indicating that with the increase of CP level, the intestinal richness also increased, and it could be seen that the colon was the main site of microbial fermentation in piglets, consistent with the experimental results of Konstantinov (Konstantinov et al., 2004). High‐throughput sequencing technology was used to study the changes in intestinal microbial bacteria in piglets, and the results were consistent with the results of previous studies (Zhou et al., 2020): Firmicutes, Bacteroidetes, and Proteobacteria were the dominant bacteria in the ileum and colon of piglets. Studies (Jonkers et al., 2012; Willing et al., 2011) have found that these three phyla are involved in nitrogen metabolism, that they secrete proteases and affect protein fermentation in the intestine, and that decreased content of these phyla can increase the likelihood of bacterial pathogen colonization.

Intestinal bacteria play a major role in protecting against pathogens, and an imbalance of bacteria can cause local pathology (Minty et al., 2019). Lactobacilli and bifidobacteria are beneficial bacteria in the intestine and can inhibit invasion of the intestine by pathogenic bacteria by fermenting food residues to produce acidic substances such as lactic acid to reduce intestinal pH or by adhering to intestinal epithelial cells (Kailasapathy & Chin, 2000). In this experiment, the relative contents of *Lactobacillus* and *Bifidobacterium* in the gut of weaned piglets showed a decreasing trend with increasing dietary protein levels, similar to the study of Ma (Peiling, 2019). The reason for this is that high levels of dietary protein disrupt the intestinal mucosa of piglets and affect its colonization by *Lactobacillus* and *Bifidobacterium*. *Roseburia* is a key bacterium in the gut that degrades dietary fiber (Kasahara et al., 2018). The results of this study showed that high dietary protein levels increased the number of *Roseburia* in the ileum and colon and are consistent with the results reported by Hooda (Hooda et al., 2013). This may be because in this experiment the CP level in the diet was adjusted primarily by changing the corn and soybean meal content of the diet; thus, changes in the protein level may have increased the dietary fiber in the diet, resulting in an increase in the intestinal content of *Roseburia*. Protein fermentation is often also accompanied by the growth of bacterial pathogens such as *E*. *coli* and *C*. *difficile*, and once the balance among the intestinal bacteria is broken, the proliferation of pathogens that produce toxins can lead to the occurrence of secretory diarrhea. However, there are very few reports on the effect of dietary protein levels on the number of intestinal *C*. *difficile*. We found that with the extension of feeding time, the number of *C*. *difficile* was very significantly higher in the 22% CP and 24% CP groups than in the 20% CP group (*p* < .01), and related studies (Tan et al., 2019) showed that the abundance of *Fusobacterium* increased in the piglet diarrhea model; therefore, we surmised that an abundance of *C*. *difficile* in the colon is an important factor causing diarrhea in weaned piglets fed high‐protein diets. Studies (Jeaurond et al., 2008; Wellock et al., 2008) found that diets containing high levels of protein contribute to the reproduction of *E. coli* in the intestine and that they also stimulate *E. coli* to produce large amounts of fermentation byproducts that reduce intestinal barrier function integrity, consistent with the results of this experiment. Some studies have found that high‐protein diets can increase the incidence of diarrhea in piglets infected with enterotoxigenic *E. coli*. Thus, the increase in dietary protein levels causes the number of pathogens in the intestine to increase, and the resulting decrease in the number of beneficial bacteria aggravates diarrhea in piglets.

## 5 CONCLUSION

Weaned piglets 25 days of age were fed corn–soybean meal‐based diets with CP levels of 18%, 20%, 22%, and 24% for one week. Elevated dietary protein levels can lead to impaired small intestinal morphology and function, decreased expression of occludin protein, increased diversity of intestinal bacteria, increased numbers of *C*. *difficile* and *E*. *coli* in the ileum and colon, decreased numbers of lactobacilli, bifidobacteria, and *Roseburia*, and increased numbers of harmful bacteria in piglets and thereby aggravate diarrhea in piglets. In contrast, a diet with a protein level of 20% is beneficial in maintaining the morphological structure and function of the small intestine and can upregulate the expression of occludin protein, reduce intercellular permeability, and maintain the barrier function of tight junctions; all of these functions are beneficial in maintaining a balance among intestinal bacteria, reducing the occurrence of diarrhea, and more effectively promoting the growth and development of piglets.

## CONFLICT OF INTEREST

The authors declare that they do not have any conflict of interest.

## ETHICS STATEMENT

This study does not involve any human or animal testing or this study was approved by the Institutional Review Board of Sichuan Agriculture University.

## THE ANIMAL WELFARE STATEMENT

The authors confirm that the ethical policies of the Journal of Animal Physiology and Animal Nutrition have been adhered to and the appropriate ethical review committee approval has been received. The authors confirm that they have followed EU standards for the protection of animals used for scientific purposes. All experimental procedures were performed in compliance with the guidelines of Sichuan Agriculture University Animal Welfare Institute (Sichuan, China).

## Data Availability

The original contributions presented in the study are included in the article/supplementary material, further inquiries can be directed to the corresponding authors.

## References

[fsn32828-bib-0001] Al‐Sadi, R. , Boivin, M. , & Ma, T. (2009). Mechanism of cytokine modulation of epithelial tight junction barrier. Frontiers in Bioscience (Landmark edition), 14, 2765–2778. 10.2741/3413 19273235 PMC3724223

[fsn32828-bib-0002] Bokulich, N. A. , Subramanian, S. , Faith, J. J. , Gevers, D. , Gordon, J. I. , Knight, R. , Mills, D. A. , & Caporaso, J. G. (2013). Quality‐filtering vastly improves diversity estimates from Illumina amplicon sequencing. Nature Methods, 10(1), 57–59. 10.1038/nmeth.2276 23202435 PMC3531572

[fsn32828-bib-0003] Buckley, A. , & Turner, J. R. (2018). Cell biology of tight junction barrier regulation and mucosal disease. Cold Spring Harbor Perspectives in Biology, 10(1), a029314. 10.1101/cshperspect.a029314 28507021 PMC5749156

[fsn32828-bib-0004] Caporaso, J. G. , Kuczynski, J. , Stombaugh, J. , Bittinger, K. , Bushman, F. D. , Costello, E. K. , Fierer, N. , Peña, A. G. , Goodrich, J. K. , Gordon, J. I. , Huttley, G. A. , Kelley, S. T. , Knights, D. , Koenig, J. E. , Ley, R. E. , Lozupone, C. A. , McDonald, D. , Muegge, B. D. , Pirrung, M. , … Knight, R. (2010). QIIME allows analysis of high‐throughput community sequencing data. Nature Methods, 7(5), 335–336. 10.1038/nmeth.f.303 20383131 PMC3156573

[fsn32828-bib-0005] Dong, Y. , Fan, H. , Yao, S. , Wang, Y. , Yao, R. , Kong, X. , & Deng, J. (2019). Effects of dietary crude protein level on digestive capacity, colonic ammonia nitrogen content and inflammatory injury in weaned piglets. Chinese Journal of Animal Nutrition, 31(08), 3561–3570.

[fsn32828-bib-0006] Edgar, R. C. (2013). UPARSE: Highly accurate OTU sequences from microbial amplicon reads. Nature Methods, 10(10), 996–998. 10.1038/nmeth.2604 23955772

[fsn32828-bib-0007] Eid, Y. Z. , Ohtsuka, A. , & Hayashi, K. (2003). Tea polyphenols reduce glucocorticoid‐induced growth inhibition and oxidative stress in broiler chickens. British Poultry Science, 44(1), 127–132. 10.1080/0007166031000085427 12737234

[fsn32828-bib-0008] Gao, J. , Yin, J. , Xu, K. , Han, H. , Liu, Z. M. , Wang, C. Y. , Li, T. J. , & Yin, Y. L. (2020). Protein level and infantile diarrhea in a postweaning piglet model. Mediators of Inflammation, 2020, 1937387. 10.1155/2020/1937387 32565721 PMC7281817

[fsn32828-bib-0009] Gophna, U. , Konikoff, T. , & Nielsen, H. B. (2017). Oscillospira and related bacteria – From metagenomic species to metabolic features. Environmental Microbiology, 19(3), 835–841. 10.1111/1462-2920.13658 28028921

[fsn32828-bib-0010] Hooda, S. , Vester Boler, B. M. , Kerr, K. R. , Dowd, S. E. , & Swanson, K. S. (2013). The gut microbiome of kittens is affected by dietary protein: Carbohydrate ratio and associated with blood metabolite and hormone concentrations. British Journal of Nutrition, 109(9), 1637–1646. 10.1017/S0007114512003479 22935193

[fsn32828-bib-0011] Htoo, J. K. , Araiza, B. A. , Sauer, W. C. , Rademacher, M. , Zhang, Y. , Cervantes, M. , & Zijlstra, R. T. (2007). Effect of dietary protein content on ileal amino acid digestibility, growth performance, and formation of microbial metabolites in ileal and cecal digesta of early‐weaned pigs. Journal of Animal Science, 85(12), 3303–3312. 10.2527/jas.2007-0105 17785591

[fsn32828-bib-0012] Jeaurond, E. A. , Rademacher, M. , Pluske, J. R. , Zhu, C. H. , & de Lange, C. F. M. (2008). Impact of feeding fermentable proteins and carbohydrates on growth performance, gut health and gastrointestinal function of newly weaned pigs. Canadian Journal of Animal Science, 88(2), 271–281. 10.4141/cjas07062

[fsn32828-bib-0013] Jonkers, D. , Penders, J. , Masclee, A. , & Pierik, M. (2012). Probiotics in the management of inflammatory bowel disease: A systematic review of intervention studies in adult patients. Drugs, 72(6), 803–823. 10.2165/11632710-000000000-00000 22512365

[fsn32828-bib-0014] Kailasapathy, K. , & Chin, J. (2000). Survival and therapeutic potential of probiotic organisms with reference to *Lactobacillus acidophilus* and *Bifidobacterium* spp. Immunology and Cell Biology, 78(1), 80–88. 10.1046/j.1440-1711.2000.00886.x 10651933

[fsn32828-bib-0015] Kasahara, K. , Krautkramer, K. A. , Org, E. , Romano, K. A. , Kerby, R. L. , Vivas, E. I. , Mehrabian, M. , Denu, J. M. , Bäckhed, F. , Lusis, A. J. , & Rey, F. E. (2018). Interactions between *Roseburia intestinalis* and diet modulate atherogenesis in a murine model. Nature Microbiology, 3(12), 1461–1471. 10.1038/s41564-018-0272-x PMC6280189 30397344

[fsn32828-bib-0016] Khounlotham, M. , Kim, W. , Peatman, E. , Nava, P. , Medina‐Contreras, O. , Addis, C. , Koch, S. , Fournier, B. , Nusrat, A. , Denning, T. L. , & Parkos, C. A. (2012). Compromised intestinal epithelial barrier induces adaptive immune compensation that protects from colitis. Immunity, 37(3), 563–573. 10.1016/j.immuni.2012.06.017 22981539 PMC3564580

[fsn32828-bib-0017] Konstantinov, S. R. , Awati, A. , Smidt, H. , Williams, B. A. , Akkermans, A. D. , & de Vos, W. M. (2004). Specific response of a novel and abundant *Lactobacillus amylovorus*‐like phylotype to dietary prebiotics in the guts of weaning piglets. Applied and Environmental Microbiology, 70(7), 3821–3830. 10.1128/AEM.70.7.3821-3830.2004 15240251 PMC444839

[fsn32828-bib-0018] Kwon, C. H. , Lee, C. Y. , Han, S. J. , Kim, S. J. , Park, B. C. , Jang, I. , & Han, J. H. (2014). Effects of dietary supplementation of lipid‐encapsulated zinc oxide on colibacillosis, growth and intestinal morphology in weaned piglets challenged with enterotoxigenic *Escherichia coli* . Animal Science Journal, 85(8), 805–813. 10.1111/asj.12215 24799095

[fsn32828-bib-0019] Liu, H. , Guo, M. , Jiang, Y. , Cao, Y. , Qian, Q. , He, X. , Huang, K. , Zhang, J. , & Xu, W. (2019). Diagnosing and tracing the pathogens of infantile infectious diarrhea by amplicon sequencing. Gut Pathogens, 11, 12. 10.1186/s13099-019-0292-y 30992716 PMC6451272

[fsn32828-bib-0020] Lochhead, J. J. , McCaffrey, G. , Quigley, C. E. , Finch, J. , DeMarco, K. M. , Nametz, N. , & Davis, T. P. (2010). Oxidative stress increases blood‐brain barrier permeability and induces alterations in occludin during hypoxia‐reoxygenation. Journal of Cerebral Blood Flow and Metabolism, 30(9), 1625–1636. 10.1038/jcbfm.2010.29 20234382 PMC2949263

[fsn32828-bib-0021] Luo, Z. , Li, C. , Cheng, Y. , Hang, S. , & Zhu, W. (2015). Effects of low dietary protein on the metabolites and microbial communities in the caecal digesta of piglets. Archives of Animal Nutrition, 69(3), 212–226. 10.1080/1745039X.2015.1034521 25908009

[fsn32828-bib-0022] Matsuki, T. , Watanabe, K. , Fujimoto, J. , Takada, T. , & Tanaka, R. (2004). Use of 16S rRNA gene‐targeted group‐specific primers for real‐time PCR analysis of predominant bacteria in human feces. Applied and Environmental Microbiology, 70(12), 7220–7228. 10.1128/AEM.70.12.7220-7228.2004 15574920 PMC535136

[fsn32828-bib-0023] Minty, M. , Canceil, T. , Serino, M. , Burcelin, R. , Terce, F. , & Blasco‐Baque, V. (2019). Oral microbiota‐induced periodontitis: A new risk factor of metabolic diseases. Reviews in Endocrine & Metabolic Disorders, 20(4), 449–459. 10.1007/s11154-019-09526-8 31741266

[fsn32828-bib-0024] Opapeju, F. O. , Krause, D. O. , Payne, R. L. , Rademacher, M. , & Nyachoti, C. M. (2009). Effect of dietary protein level on growth performance, indicators of enteric health, and gastrointestinal microbial ecology of weaned pigs induced with postweaning colibacillosis. Journal of Animal Science, 87(8), 2635–2643. 10.2527/jas.2008-1310 19395520

[fsn32828-bib-0025] Peiling, M. (2019). Effects of Low Protein on Performance and Intestinal Microflora of Piglets. Proceedings of 2019 1st International Biology and Medicine Conference (pp. 177–182). Francis Academic Press, UK.

[fsn32828-bib-0026] Peng, X. , Hu, L. , Liu, Y. , Yan, C. , Fang, Z. F. , Lin, Y. , Xu, S. Y. , Li, J. , Wu, C. M. , Chen, D. W. , Sun, H. , Wu, D. , & Che, L. Q. (2016). Effects of low‐protein diets supplemented with indispensable amino acids on growth performance, intestinal morphology and immunological parameters in 13 to 35 kg pigs. Animal: An International Journal of Animal Bioscience, 10(11), 1812–1820. 10.1017/S1751731116000999 27210003

[fsn32828-bib-0027] Peng, Y. , Yu, K. , Mu, C. , Hang, S. , Che, L. , & Zhu, W. (2017). Progressive response of large intestinal bacterial community and fermentation to the stepwise decrease of dietary crude protein level in growing pigs. Applied Microbiology and Biotechnology, 101(13), 5415–5426. 10.1007/s00253-017-8285-6 28455617

[fsn32828-bib-0028] Roca, M. , Nofrarías, M. , Majó, N. , Pérez de Rozas, A. M. , Segalés, J. , Castillo, M. , Martín‐Orúe, S. M. , Espinal, A. , Pujols, J. , & Badiola, I. (2014). Changes in bacterial population of gastrointestinal tract of weaned pigs fed with different additives. BioMed Research International, 2014, 269402. 10.1155/2014/269402 24575403 PMC3915759

[fsn32828-bib-0029] Roxas, J. L. , Koutsouris, A. , Bellmeyer, A. , Tesfay, S. , Royan, S. , Falzari, K. , Harris, A. , Cheng, H. , Rhee, K. J. , & Hecht, G. (2010). Enterohemorrhagic *E. coli* alters murine intestinal epithelial tight junction protein expression and barrier function in a Shiga toxin independent manner. Laboratory Investigation; A Journal of Technical Methods and Pathology, 90(8), 1152–1168. 10.1038/labinvest.2010.91 20479715 PMC2912457

[fsn32828-bib-0030] Santiago, A. , Pozuelo, M. , Poca, M. , Gely, C. , Nieto, J. C. , Torras, X. , Román, E. , Campos, D. , Sarrabayrouse, G. , Vidal, S. , Alvarado‐Tapias, E. , Guarner, F. , Soriano, G. , Manichanh, C. , & Guarner, C. (2016). Alteration of the serum microbiome composition in cirrhotic patients with ascites. Scientific Reports, 6, 25001. 10.1038/srep25001 27112233 PMC4845009

[fsn32828-bib-0031] Sappington, P. L. , Han, X. , Yang, R. , Delude, R. L. , & Fink, M. P. (2003). Ethyl pyruvate ameliorates intestinal epithelial barrier dysfunction in endotoxemic mice and immunostimulated caco‐2 enterocytic monolayers. The Journal of Pharmacology and Experimental Therapeutics, 304(1), 464–476. 10.1124/jpet.102.043182 12490623

[fsn32828-bib-0032] Shil, A. , Olusanya, O. , Ghufoor, Z. , Forson, B. , Marks, J. , & Chichger, H. (2020). Artificial sweeteners disrupt tight junctions and barrier function in the intestinal epithelium through activation of the sweet taste receptor, T1R3. Nutrients, 12(6), 1862. 10.3390/nu12061862 PMC7353258 32580504

[fsn32828-bib-0033] Taborda, R. L. M. , Silva, L. A. D. , Orlandi, P. P. , Batista, F. S. , Rodrigues, R. S. , & Matos, N. B. (2018). Characterization of enteroaggregative *Escherichia coli* among diarrheal children in Western Brazilian Amazon. Arquivos De Gastroenterologia, 55(4), 390–396. 10.1590/S0004-2803.201800000-84 30785524

[fsn32828-bib-0034] Tan, Z. , Dong, W. , Ding, Y. , Ding, X. , Zhang, Q. , & Jiang, L. (2019). Changes in cecal microbiota community of suckling piglets infected with porcine epidemic diarrhea virus. PLoS One, 14(7), e0219868. 10.1371/journal.pone.0219868 31310635 PMC6634403

[fsn32828-bib-0035] Teng, P. Y. , Yadav, S. , Castro, F. L. S. , Tompkins, Y. H. , Fuller, A. L. , & Kim, W. K. (2020). Graded Eimeria challenge linearly regulated growth performance, dynamic change of gastrointestinal permeability, apparent ileal digestibility, intestinal morphology, and tight junctions of broiler chickens. Poultry Science, 99(9), 4203–4216. 10.1016/j.psj.2020.04.031 PMC7598010 32867964

[fsn32828-bib-0036] Turkez, H. , Geyikoglu, F. , Yousef, M. I. , Celik, K. , & Bakir, T. O. (2012). Ameliorative effect of supplementation with L‐glutamine on oxidative stress, DNA damage, cell viability and hepatotoxicity induced by 2,3,7,8‐tetrachlorodibenzo‐p‐dioxin in rat hepatocyte cultures. Cytotechnology, 64(6), 687–699. 10.1007/s10616-012-9449-y 22453904 PMC3488374

[fsn32828-bib-0037] Wellock, I. J. , Fortomaris, P. D. , Houdijk, J. G. , & Kyriazakis, I. (2008). Effects of dietary protein supply, weaning age and experimental enterotoxigenic *Escherichia coli* infection on newly weaned pigs: Health. Animal: An International Journal of Animal Bioscience, 2(6), 834–842. 10.1017/S1751731108002048 22443662

[fsn32828-bib-0038] Wijtten, P. J. , van der Meulen, J. , & Verstegen, M. W. (2011). Intestinal barrier function and absorption in pigs after weaning: A review. British Journal of Nutrition, 105(7), 967–981. 10.1017/S0007114510005660 21303573

[fsn32828-bib-0039] Willing, B. P. , Russell, S. L. , & Finlay, B. B. (2011). Shifting the balance: Antibiotic effects on host‐microbiota mutualism. Nature Reviews Microbiology, 9(4), 233–243. 10.1038/nrmicro2536 21358670

[fsn32828-bib-0040] Wu, Y. , Jiang, Z. , Zheng, C. , Wang, L. I. , Zhu, C. , Yang, X. , Wen, X. , & Ma, X. (2015). Effects of protein sources and levels in antibiotic‐free diets on diarrhea, intestinal morphology, and expression of tight junctions in weaned piglets. Anim Nutr, 1(3), 170–176. 10.1016/j.aninu.2015.08.013 29767171 PMC5945934

[fsn32828-bib-0041] Xie, C. , Duan, X. , Long, C. , & Wu, X. (2020). Hepatic lipid metabolism is affected by a daily 3‐meal pattern with varying dietary crude protein with a pig model. Animal Nutrition, 6(1), 16–23. 10.1016/j.aninu.2019.11.001 32211524 PMC7082684

[fsn32828-bib-0042] Yang, Y.‐W. , Chen, M.‐K. , Yang, B.‐Y. , Huang, X.‐J. , Zhang, X.‐R. , He, L.‐Q. , Zhang, J. , & Hua, Z.‐C. (2015). Use of 16S rRNA gene‐targeted group‐specific primers for real‐time PCR analysis of predominant bacteria in mouse feces. Applied and Environmental Microbiology, 81(19), 6749–6756. 10.1128/AEM.01906-15 26187967 PMC4561689

[fsn32828-bib-0043] Zhang, B. , & Guo, Y. (2009). Supplemental zinc reduced intestinal permeability by enhancing occludin and zonula occludens protein‐1 (ZO‐1) expression in weaning piglets. British Journal of Nutrition, 102(5), 687–693. 10.1017/S0007114509289033 19267955

[fsn32828-bib-0044] Zhang, H. , Wielen, N. V. , Hee, B. V. , Wang, J. , Hendriks, W. , & Gilbert, M. (2020). Impact of fermentable protein, by feeding high protein diets, on microbial composition, microbial catabolic activity, gut health and beyond in pigs. Microorganisms, 8(11), 1735. 10.3390/microorganisms8111735 PMC7694525 33167470

[fsn32828-bib-0045] Zhao, Y. , Qin, G. , Sun, Z. , Che, D. , Bao, N. , & Zhang, X. (2011). Effects of soybean agglutinin on intestinal barrier permeability and tight junction protein expression in weaned piglets. International Journal of Molecular Sciences, 12(12), 8502–8512. 10.3390/ijms12128502 22272087 PMC3257084

[fsn32828-bib-0046] Zhou, J. , Wang, Y. , Zeng, X. , Zhang, T. , Li, P. , Yao, B. , Wang, L. U. , Qiao, S. , & Zeng, X. (2020). Effect of antibiotic‐free, low‐protein diets with specific amino acid compositions on growth and intestinal flora in weaned pigs. Food & Function, 11(1), 493–507. 10.1039/c9fo02724f 31833513

[fsn32828-bib-0047] Zihni, C. , Mills, C. , Matter, K. , & Balda, M. S. (2016). Tight junctions: From simple barriers to multifunctional molecular gates. Nature Reviews Molecular Cell Biology, 17(9), 564–580. 10.1038/nrm.2016.80 27353478

